# Regioselective C–H Thiocyanation of Arenes by Iron(III) Chloride Catalysis

**DOI:** 10.1021/acs.joc.3c00454

**Published:** 2023-05-09

**Authors:** Lachlan J. N. Waddell, Maisie R. Senkans, Andrew Sutherland

**Affiliations:** School of Chemistry, The Joseph Black Building, University of Glasgow, Glasgow G12 8QQ, U.K.

## Abstract

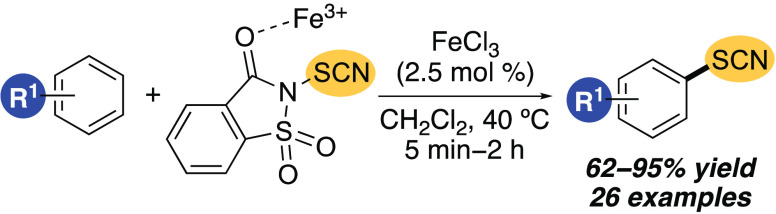

Aryl thiocyanates are flexible synthetic intermediates that can be used in the preparation of a diverse range of arene building blocks for medicinal chemistry. Here, we report a fast and efficient Lewis acid-catalyzed method for the regioselective thiocyanation of arenes. Iron(III) chloride was found to be an effective Lewis acid for the activation of *N*-thiocyanatosaccharin and the subsequent thiocyanation of a wide range of activated arenes. The procedure was applicable for the thiocyanation of biologically active compounds such as metaxalone and an estradiol derivative and was used as part of a one-pot tandem iron-catalytic process for the regioselective, dual functionalization of an arene building block.

## Introduction

**Scheme 1:**
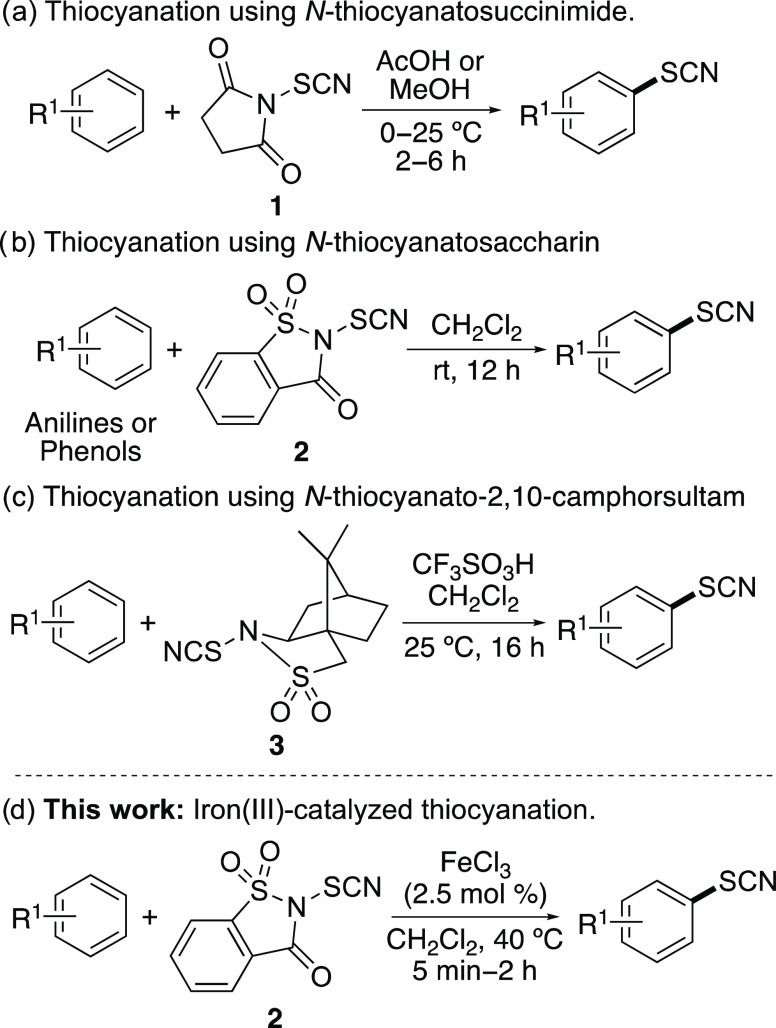
Thiocyanation of Arenes Using Electrophilic *N*-Thiocyanato Reagents

Organothiocyanates are important compounds, found as key components of biologically active molecules and natural products.^1^ These are also used as synthetic intermediates for the preparation of various sulfur-containing organic compounds.^2^ In this regard, aryl thiocyanates are versatile building blocks for access to functionalized aromatic compounds, including medicinally important aryl trifluoromethyl thioethers.^2,3^ A key approach for the synthesis of aryl thiocyanates is the cyanation of sulfur-containing arenes.^4^ This includes methods such as the copper-catalyzed cyanation of disulfides with azobisisobutyronitile^4b^ and the direct photocatalytic cyanation of aryl thiols by cleavage of the C–S bond of ammonium thiocyanate.^4d^ The other main approach is the direct thiocyanation of arenes using electrophilic thiocyanating reagents.^2,5^ Historically, electrophilic reagents, such as thiocyanogen chloride prepared from thiocyanogen and chlorine gas, were used for the functionalization of arenes.^6^ More recently, bench-stable, electrophilic *N*-thiocyanating reagents have been developed for the functionalization of aromatic compounds. In 1995, Still and co-workers reported *N*-thiocyanatosuccinimide (NTS, **1**) prepared from *N*-bromosuccinimide and sodium thiocyanate for the thiocyanation of (hetero)arenes (Scheme 1a).^7^ Using three equivalents of NTS **1** under mild conditions gave the thiocyanated products in good to excellent yields. In 2018, Chen and co-workers reported the synthesis and application of *N*-thiocyanatosaccharin (**2**).^8^ As well as demonstrating this as an effective reagent for the thiocyanation of β-keto carbonyl compounds and oxindoles, *N*-thiocyanatosaccharin (**2**) was also shown to functionalize activated aromatic compounds (Scheme 1b). Phenols and electron-rich anilines yielded mainly *p*-thiocyanated products under mild conditions with reaction times of 12 h, while anilines bearing electron-deficient substituents gave *N*-thiocyanated products. In 2019, the Chen group reported *N*-thiocyanato-dibenzenesulfonimide as a thiocyanating reagent with enhanced reactivity.^9^ Thiocyanation of activated arenes using this reagent was complete in 10 min at 40 °C and gave the products in high yields, while less activated aromatic compounds such as *m*-xylene were thiocyanated using triflic acid as an additive. In 2021, Besset and co-workers reported various *N*-thiocyanato-2,10-camphorsultam derivatives for the thiocyanation of organic compounds.^10^ Although these reagents were designed for asymmetric thiocyanation of sp^3^ centers, these were shown to functionalize (hetero)arenes in moderate to high yields, using triflic acid activation (Scheme 1c).

We have shown that iron(III) salts can act as Lewis acids for the activation of *N*-halosuccinimides and the subsequent regioselective halogenation of arenes.^11^ More recently, we have shown that iron(III) triflimide can activate *N*-thioaryl succinimides for the preparation of unsymmetrical biaryl sulfides.^12^ Based on this previous work, we proposed that an iron(III) salt may function as an activator of an *N*-thiocyanating reagent, allowing efficient thiocyanation of a wide range of arenes, without the requirement of acidic conditions or long reaction times. Here, we report the use of iron(III) chloride as a Lewis acid catalyst for the activation of *N*-thiocyanatosaccharin (**2**) and the subsequent thiocyanation of various arenes. We also demonstrate the application of this method for the late-stage thiocyanation of biologically active compounds and as the key step in a tandem iron-catalytic process for the one-pot dual functionalization of an arene building block.

## Results and Discussion

*N*-Thiocyanatosaccharin (**2**) was chosen as the electrophilic reagent for this study due to its straightforward synthesis and higher reactivity compared to succinimide and phthalimide reagents.^5d,8^ Initially, **2** was screened for the thiocyanation of anisole (**4a**) (Table 1). Using 1.2 equiv of **2** in the presence of iron(III) triflimide (2.5 mol %) at a reaction temperature of 20 °C required a reaction time of 96 h and gave 4-thiocyanatoanisole (**5a**) in 74% yield (entry 1).^13^ On increasing the temperature to 40 °C, the reaction was complete after 2 h and gave **5a** in an improved yield of 95% (entry 2). Other Lewis acids known to effect electrophilic aromatic substitution reactions were also screened (entries 3–6). The fastest reaction was observed using iron(III) chloride (entry 3). With a catalyst loading of 2.5 mol %, the transformation was complete after 0.5 h and gave **5a** in 93% yield. Interestingly, at the same catalyst loading, aluminum(III) chloride gave no product (entry 4), while both silver(I) triflimide and indium(III) triflate gave excellent yields of **5a**, although after much longer reaction times (entries 5 and 6).^3a,14^ It should be noted that in the absence of any Lewis acid, minimal conversion was observed even after an extended reaction time (48 h, entry 7).

**Table 1:**
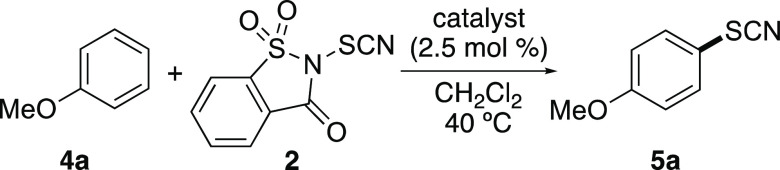
Optimization Studies for Thiocyanation of Anisole (**4a**)

entry	catalyst	temperature (°C)	time (h)	yield (%)^a^
1^b^	Fe(NTf_2_)_3_	20	96	74
2^b^	Fe(NTf_2_)_3_	40	2	95
3	FeCl_3_	40	0.5	93
4	AlCl_3_	40	24	0
5	AgNTf_2_	40	24	93
6	In(OTf)_3_	40	24	82
7		40	48	<5

a Isolated yields.

b Fe(NTf_2_)_3_ was prepared *in situ* from FeCl_3_ (2.5 mol %) and [BMIM]NTf_2_ (7.5 mol %).

**Scheme 2:**
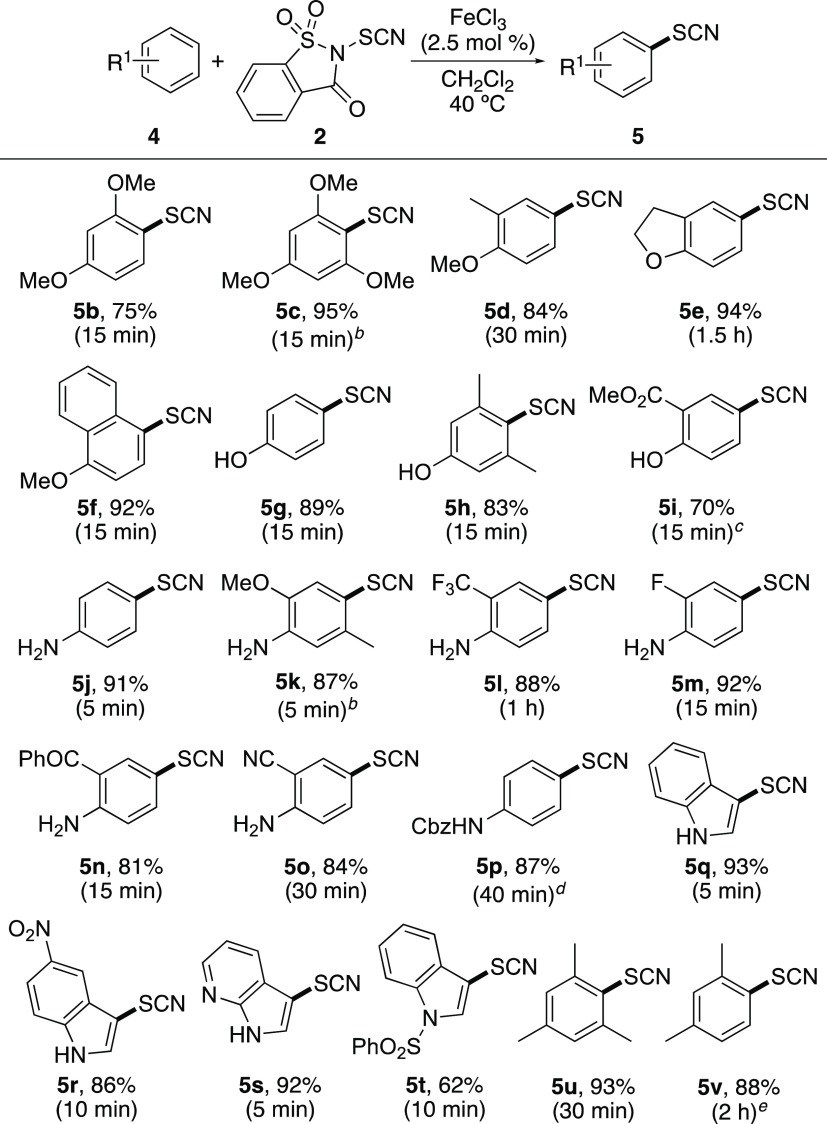
Reaction Scope of Iron(III) Chloride-Catalyzed Thiocyanation of Arenes Isolated yields. Reaction done at 0 °C. Reaction done using FeCl_3_ (10 mol %) and Ph_2_Se (10 mol %). Reaction done using FeCl_3_ (2.5 mol %) and Ph_2_Se (2.5 mol %). Reaction done using FeCl_3_ (10 mol %).

**Scheme 3:**
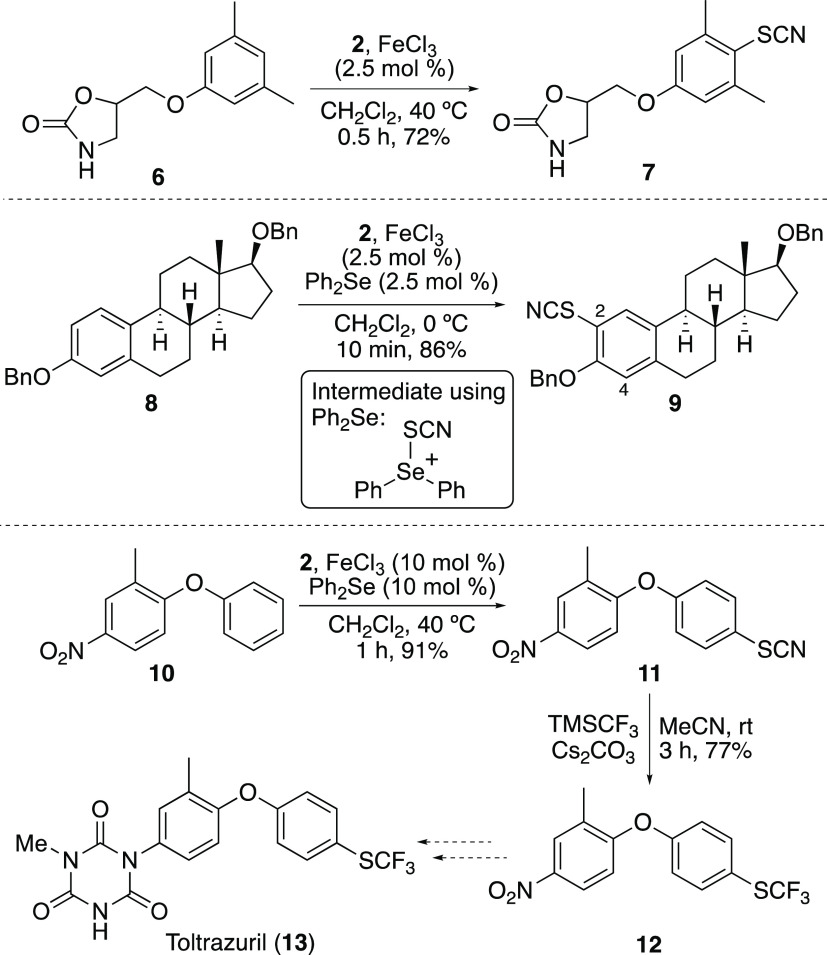
Biological Applications of Iron(III) Chloride-Catalyzed Thiocyanation Isolated yields.

Having identified iron(III) chloride as the most effective Lewis acid in performing both rapid and efficient thiocyanation reactions, this was used to examine the scope of the transformation (Scheme 2).^15^ Standard electron-rich arenes, such as anisoles, phenols, anilines, and indoles, were found to undergo fast transformations with typical reaction times of 5–30 min. In all cases, only the *para*-substituted products were observed in high yields. Deactivated arenes such as bromobenzene were not tolerated; however, aromatic compounds with electron-deficient substituents but containing at least one activating group were good substrates for the thiocyanation reaction. For example, thiocyanation of anilines with deactivating groups such as 2-fluoroaniline (**4m**) and 2-aminobenzophenone (**4n**) was complete in 15 min, while 2-trifluoromethylaniline (**4l**) and 2-aminobenzonitrile (**4o**) required marginally longer reaction times (1 h and 0.5 h, respectively). Under the standard conditions, two compounds, methyl salicylate (**4i**) and *N*-Cbz-protected aniline **4p**, were shown to require longer reaction times of 20 h to achieve complete conversion. Lewis bases such as diaryl selenides have been shown to accelerate challenging Lewis acid-catalyzed thioarylations by forming a more reactive cationic intermediate (see Scheme 3).^12c,16^ Employing diphenyl selenide as a Lewis base catalyst during the iron(III)-catalyzed thiocyanation of **4i** and **4p** significantly improved the reaction times. The thiocyanated salicylate **5i** was formed in 70% yield after a 15 min reaction time, while **5p** was isolated in 87% yield following a 40 min reaction. It should be noted that for all anilines bearing electron-deficient substituents in this study, *N*-thiocyanated products were never observed. The extent of the scope of this transformation was demonstrated by the thiocyanation of minimally activated arenes. Under standard conditions, the reaction with mesitylene (**4u**) was complete after 0.5 h to give mono-thiocyanated product **5u** in 93% yield. With the same catalyst loading, *m*-xylene (**4v**) reached 62% conversion after 24 h. In this case, an increase of catalyst loading to 10 mol % allowed complete conversion after 2 h and an 88% yield of **5v**. While effective reactions were possible with mesitylene and *m*-xylene, no reaction was observed with toluene. Using a catalyst loading of 10 mol % and standard reaction conditions, no conversion was observed after 72 h. During the substrate screening process, the scalability of the transformation was also investigated. Thiocyanation of anisole (**4a**) on a one-gram scale using the optimized conditions proceeded in a similar manner, with completion after 0.5 h and a 95% yield of 4-thiocyanatoanisole (**5a**).

The study then focused on demonstrating the use of iron(III) chloride-catalyzed thiocyanations for the preparation or derivatization of biologically active compounds (Scheme 3). Initial investigations focused on the thiocyanation of metaxalone (**6**), a drug used for pain relief and as a muscle relaxant.^17^ Using iron(III) chloride (2.5 mol %) and standard reaction conditions, thiocyanation was complete after 0.5 h. Analysis of the reaction mixture by ^1^H NMR spectroscopy revealed a 6:1 ratio of the *para*- and *ortho*-products. Separation by column chromatography gave the major *para*-product **7** in 72% yield.

Iron(III)-catalyzed thiocyanation of estradiol was next investigated. During screening of the reaction scope, a limitation was observed during attempted *ortho*-thiocyanation of phenols. Thiocyanation of *para*-cresol gave a mixture of inseparable products. We propose that as well as the *ortho*-thiocyanated product, 5-membered oxathioimines, generated by intramolecular cyclization of the hydroxyl group with the thiocyanate moiety and observed by Bhat and co-workers during their oxidative *ortho*-thiocyanation of phenols, are also formed.^18^ To prevent similar side reactions with estradiol, studies focused on the thiocyanation of dibenzyl derivative **8** (Scheme 3). An initial reaction with iron(III) chloride (2.5 mol %) at 0 °C and a reaction time of 1 h resulted in clean thiocyanation but at both *ortho*-positions. Analysis by ^1^H NMR spectroscopy showed a 3:2 ratio of 2- and 4-thiocyanated regioisomers, respectively. In an attempt to accelerate the reaction and improve the regioselectivity, the reaction was repeated using diphenyl selenide as a Lewis base catalyst (2.5 mol %). In this case, the reaction was complete after 10 min and gave an improved 10:1 ratio of regioisomers in favor of 2-thiocyanated product **9**. Purification by column chromatography gave **9** in 86% yield. Here, the in situ-generated thiocyanated-diphenyl selenide cation (see insert) is more sterically hindered than the iron-activated *N*-thiocyanatosaccharin and thus leads to selective thiocyanation at the most accessible *ortho*-position.

The iron(III)-catalyzed thiocyanation reaction was also investigated for the efficient synthesis of biaryl ether **12**, which is a key intermediate for the synthesis of toltrazuril (**13**), used to treat coccidiosis in livestock and poultry.^19^ We proposed that an iron(III)-catalyzed thiocyanation could be used for a two-step synthesis of **12** from 2-methyl-4-nitro-1-phenoxybenzene (**10**) (Scheme 3). Initial attempts at thiocyanation of **10** demonstrated that a 10 mol % catalyst loading of iron(III) chloride was required for good conversion. Although this resulted in selective *para*-thiocyanation of the electron-rich phenyl ether, a reaction time of 18 h was required and gave **11** in moderate yield (43%). To improve the reaction rate and yield, the transformation was repeated using diphenyl selenide as a Lewis base catalyst. This gave **11** after a 1 h reaction time in 91% yield. Conversion of the thiocyanate group to the trifluomethyl thioether was achieved using a Langlois-type nucleophilic substitution, reported by Goossen and co-workers.^3a,20^ Reaction of **11** with the Ruppert-Prakash reagent^21,22^ TMS-CF_3_ under basic conditions gave trifluoromethyl thioether **12** in 77% yield, thereby completing the formal synthesis of toltrazuril.

**Scheme 4:**
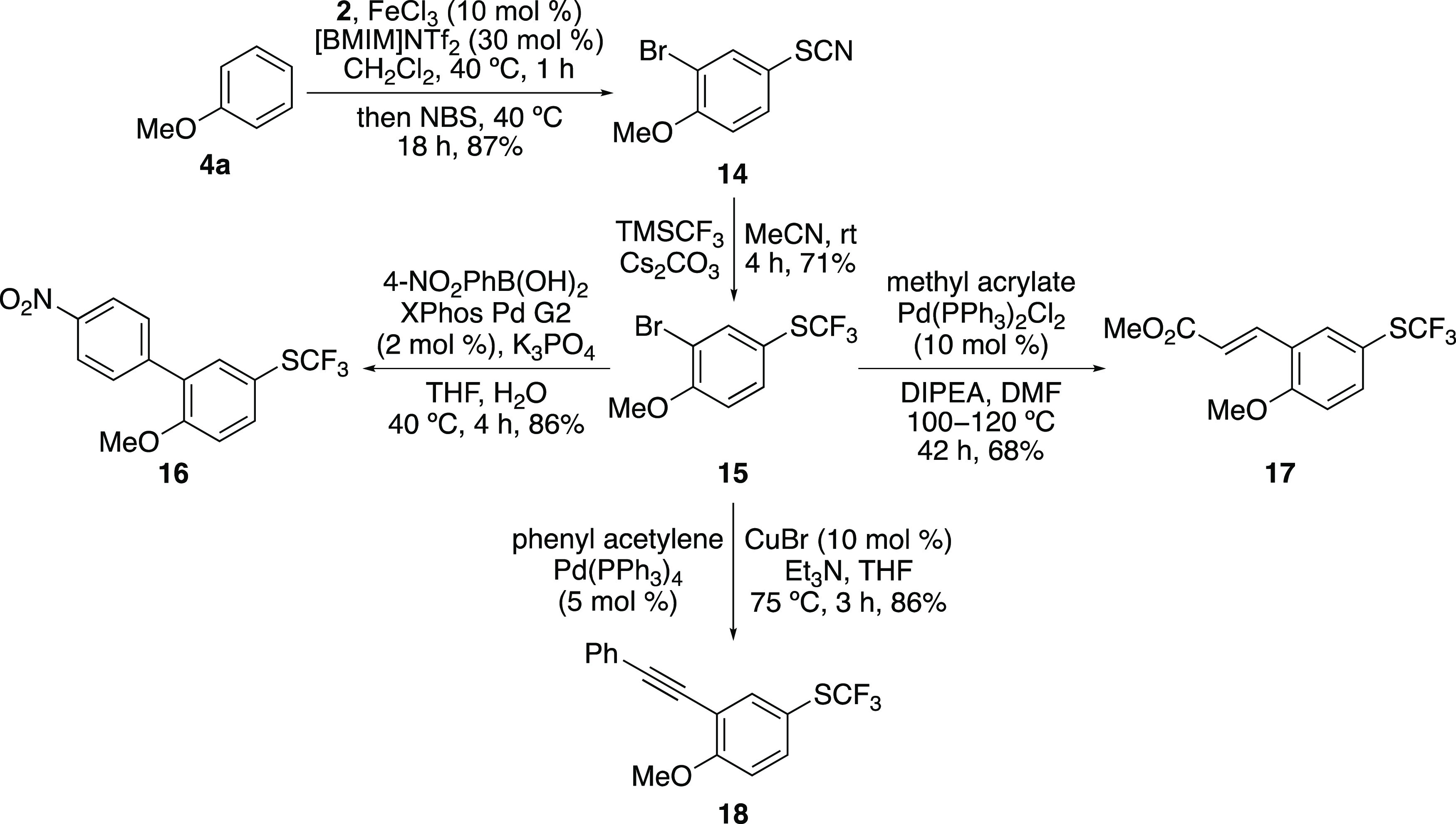
Synthetic Application of Iron(III)-Catalyzed Thiocyanation Isolated yields.

The project next investigated the use of iron(III)-catalyzed thiocyanation for the rapid preparation of multifunctional synthetic building blocks for medicinal chemistry. Due to the mild nature of iron(III)-catalyzed arene substitution reactions, we proposed that one-pot tandem iron-catalyzed processes involving dual functionalization could be used to prepare arene building blocks for diversification. Using anisole (**4a**), a one-pot, dual iron(III)-catalyzed *para*-thiocyanation and *ortho*-bromination was attempted (Scheme 4). Initial reactions demonstrated that while *para*-thiocyanation proceeded rapidly using iron(III) chloride (2.5 mol %) and *N*-thiocyanatosaccharin (**2**), the more challenging second-stage *ortho*-bromination with *N*-bromosuccinimide (NBS) required the use of the stronger Lewis acid, iron(III) triflimide, and an increased catalyst loading.^11b^ Therefore, iron(III) triflimide (10 mol %) was added at the start of the one-pot process and resulted in fast *para*-thiocyanation (1 h), followed by slower *ortho*-bromination (18 h). This gave 2-bromo-4-thiocyanatoanisole (**14**) as the sole product in 87% yield. Scale-up (3.3 mmol) demonstrated the compatibility of both transformations as part of a one-pot process, with the isolation of the dual functionalized product **14** in similarly high yields. The use of **14** as a synthetic building block was then established. A Langlois-type reaction with the Ruppert-Prakash reagent was used to introduce the medicinally relevant trifluoromethyl thioether group and gave **15** in 71% yield.^3a,23^ Then, standard palladium-catalyzed cross-coupling reactions were used to diversify the *ortho*-position, with the introduction of aryl, alkenyl, and alkynyl substituents in good to high yields. Thus, diverse aryl synthetic building blocks can be readily accessed using the iron(III)-catalyzed thiocyanation reaction as part of a one-pot dual functionalization process, followed by selective transformation of each functional group.

**Figure 1:**
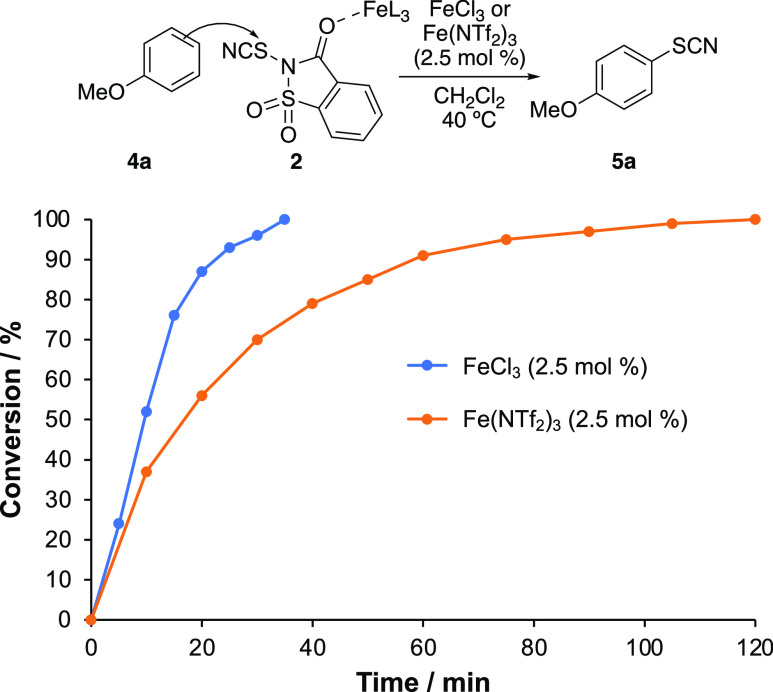
Conversion graphs for the reaction of anisole (**4a**) with *N*-thiocyanatosaccharin (**2**), catalyzed by either FeCl_3_ (2.5 mol %) or Fe(NTf_2_)_3_ (2.5 mol %) (measured by ^1^H NMR spectroscopy, using dimethyl terephthalate as an internal standard).

Previous work in our group has shown that iron(III) triflimide activation of *N*-halo or *N*-thioaryl succinimides results in faster arene substitution reactions than with iron(III) chloride.^11a,12a^ This is in agreement with other applications of metal triflimide salts in organic reactions, in which the high electronegativity, large volume, and low charge density of the triflimide counterion allows the metal cation to act as a super Lewis acid.^24^ In contrast, this study has shown that iron(III) chloride catalyzed faster thiocyanation of anisole (**4a**) compared to iron(III) triflimide (Table 1, 0.5 versus 2 h). Conversion graphs clearly show the difference in rates between the two catalysts (Figure 1). Similar differences in reaction rates were observed during the iron-catalyzed thiocyanation of methyl salicylate (**4i**) and *m*-xylene (**4v**).^15^ Previous studies using *N*-thiosaccharin reagents have suggested that activation with Bronsted or Lewis acids occurs via coordination with the amide oxygen atom.^25^ We propose that the iron salts bind in a similar manner during the thiocyanation of arenes using *N*-thiocyanatosaccharin (**2**) (Figure 1). Although iron(III) triflimide is a stronger Lewis acid, we believe that the larger steric hindrance of this metal salt accounts for the slightly slower activation of the relatively bulky *N*-thiocyanatosaccharin (**2**) than with the smaller, weaker Lewis acid, iron(III) chloride. This rational also explains why faster electrophilic substitution reactions are observed using iron(III) triflimide with smaller succinimide-based reagents.^11a,12a^

## Conclusions

In summary, iron(III) chloride has been shown as an effective Lewis acid catalyst for the regioselective *para*-thiocyanation of activated arenes with *N*-thiocyanatosaccharin (**2**). Excellent scope and fast reaction times of 5–30 min were observed for anisoles, phenols, anilines, and indoles. Arenes with weak activating groups, such as mesitylene and *m*-xylene, were also found to undergo a fast thiocyanation reaction. Many activated arenes bearing electron-deficient substituents were also substrates for this transformation, with diphenyl selenide used as a Lewis base catalyst to maintain short reaction times for some of the compounds. The synthetic utility of the transformation for the thiocyanation of more complex arenes was demonstrated with the high-yielding and selective functionalization of biologically active compounds such as metaxalone and a protected estradiol derivative. The thiocyanation reaction was also used as part of a one-pot iron-catalyzed process for the dual functionalization of anisole. Application of the resulting product as a synthetic building block was realized with the selective introduction of a trifluoromethyl thioether substituent using a Langlois-type reaction of the thiocyanate group, followed by various palladium-catalyzed cross-coupling reactions at the *ortho*-bromide position. Investigation of other arene functionalization reactions using iron(III) salts are currently underway.

## Experimental Section

All reagents and starting materials were obtained from commercial sources and used as received. *N*-Thiocyanatosaccharin (**2**),^8^ β-estradiol dibenzyl ether (**8**),^26^ and 2-methyl-4-nitro-1-phenoxybenzene (**10**)^27^ were prepared according to the literature. Reactions were performed open to air unless otherwise mentioned. All reactions performed at elevated temperatures were heated using an oil bath. Brine refers to a saturated aqueous solution of sodium chloride. Flash column chromatography was performed using silica gel 60 (40–63 μm). Aluminum-backed plates precoated with silica gel 60F_254_ were used for thin-layer chromatography and were visualized with a UV lamp or by staining with potassium permanganate. ^1^H NMR spectra were recorded on a NMR spectrometer at either 400 or 500 MHz, and data are reported as follows: chemical shift in ppm relative to tetramethylsilane or the solvent as an internal standard (CHCl_3_, δ 7.26 ppm; DMSO, δ 2.50 ppm), multiplicity (s = singlet, d = doublet, t = triplet, q = quartet, m = multiplet or overlap of nonequivalent resonances, integration). The abbreviation br s refers to broad singlet. ^13^C NMR spectra were recorded on a NMR spectrometer at either 101 or 126 MHz, and data are reported as follows: chemical shift in ppm relative to tetramethylsilane or the solvent as an internal standard (CDCl_3_, δ 77.0 ppm; DMSO-d_6_, δ 39.5 ppm), multiplicity with respect to hydrogen (deduced from DEPT experiments, C, CH, CH_2_, or CH_3_). Infrared spectra were recorded on a FTIR spectrometer; wavenumbers are indicated in cm^–1^. Mass spectra were recorded using an electrospray technique. HRMS spectra were recorded using a dual-focusing magnetic analyzer mass spectrometer. Melting points are uncorrected. Optical rotations were determined as solutions irradiating with the sodium D line (λ = 589 nm) using a polarimeter. [α]_D_ values are given in units of 10^–1^ deg cm^–1^ g^–1^.

### General Procedure: 4-Thiocyanatoanisole (**5a**)^4e^

To a solution of *N*-thiocyanatosaccharin (**2**) (0.0960 g, 0.400 mmol) and iron(III) chloride (0.00135 g, 0.00832 mmol, 2.5 mol %) in dry dichloromethane (2 mL) under argon was added anisole (**4a**) (0.0362 mL, 0.333 mmol). The reaction mixture was stirred at 40 °C in the absence of light for 0.5 h. After cooling to room temperature, the reaction mixture was diluted with dichloromethane (10 mL) and washed with water (10 mL). The aqueous layer was extracted with dichloromethane (2 × 10 mL), and the combined organic layers were washed with brine (20 mL). The organic phase was dried (MgSO_4_), filtered, and concentrated *in vacuo*. Purification by flash column chromatography (15% diethyl ether in hexane) gave 4-thiocyanatoanisole (**5a**) (0.0514 g, 93%) as a colorless oil. Spectroscopic data were consistent with the literature.^4e^^1^H NMR (CDCl_3_, 400 MHz): δ 7.53–7.48 (m, 2H), 6.97–6.93 (m, 2H), 3.83 (s, 3H); ^13^C{^1^H} NMR (CDCl_3_, 101 MHz): δ 161.5 (C), 134.0 (2 × CH), 116.0 (2 × CH), 114.0 (C), 111.8 (C), 55.7 (CH_3_); MS (ESI) *m*/*z* 166 (M + H^+^, 100).

### 4-Thiocyanatoanisole (**5a**)—Large-Scale Reaction^4e^

To a solution of *N*-thiocyanatosaccharin (**2**) (2.67 g, 11.1 mmol) and iron(III) chloride (0.0375 g, 0.231 mmol, 2.5 mol %) in dry dichloromethane (60 mL) under argon was added anisole (**4a**) (1.01 mL, 9.25 mmol). The reaction mixture was stirred at 40 °C in the absence of light for 0.5 h. After cooling to room temperature, the reaction mixture was diluted with dichloromethane (50 mL) and washed with water (50 mL). The aqueous layer was extracted with dichloromethane (2 × 50 mL), and the combined organic layers were washed with brine (100 mL). The organic phase was dried (MgSO_4_), filtered, and concentrated *in vacuo*. Purification by flash column chromatography (15% diethyl ether in hexane) gave 4-thiocyanatoanisole (**5a**) (1.45 g, 95%) as a colorless oil. Spectroscopic data as described above.

### 2,4-Dimethoxy-1-thiocyanatobenzene (**5b**)^28^

The reaction was performed according to the general procedure using 1,3-dimethoxybenzene (**4b**) (0.0655 mL, 0.500 mmol) and *N*-thiocyanatosaccharin (**2**) (0.144 g, 0.600 mmol). The reaction mixture was stirred at 40 °C for 0.25 h. Purification by flash column chromatography (30% ethyl acetate in hexane) gave 2,4-dimethoxy-1-thiocyanatobenzene (**5b**) (0.0733 g, 75%) as a colorless oil. Spectroscopic data were consistent with the literature.^28^^1^H NMR (CDCl_3_, 500 MHz): δ 7.46 (d, 1H, *J* = 8.6 Hz), 6.54 (dd, 1H, *J* = 8.6, 2.5 Hz), 6.51 (d, 1H, d, *J* = 2.5 Hz), 3.91 (s, 3H), 3.83 (s, 3H); ^13^C{^1^H} NMR (CDCl_3_, 101 MHz): δ 163.2 (C), 159.2 (C), 134.0 (CH), 111.4 (C), 106.3 (CH), 102.7 (C), 99.8 (CH), 56.3 (CH_3_), 55.8 (CH_3_); MS (ESI) *m*/*z* 218 (M + Na^+^, 100).

### 1,3,5-Trimethoxy-2-thiocyanatobenzene (**5c**)^29^

The reaction was performed according to the general procedure using 1,3,5-trimethoxybenzene (**4c**) (0.0841 g, 0.500 mmol) and *N*-thiocyanatosaccharin (**2**) (0.144 g, 0.600 mmol). The reaction mixture was stirred at 0 °C for 0.25 h. Purification by flash column chromatography (30% ethyl acetate in hexane) gave 1,3,5-trimethoxy-2-thiocyanatobenzene (**5c**) (0.107 g, 95%) as a white solid. Mp 151–153 °C (lit.^29^ 151–152 °C); ^1^H NMR (CDCl_3_, 400 MHz): δ 6.15 (s, 2H), 3.91 (s, 6H), 3.84 (s, 3H); ^13^C{^1^H} NMR (CDCl_3_, 101 MHz): δ 164.4 (C), 161.5 (2 × C), 112.0 (C), 91.5 (2 × CH), 89.9 (C), 56.5 (2 × CH_3_), 55.7 (CH_3_); MS (ESI) *m*/*z* 226 (M + H^+^, 100).

### 2-Methyl-4-thiocyanatoanisole (**5d**)^8^

The reaction was performed according to the general procedure using 2-methylanisole (**4d**) (0.0620 mL, 0.500 mmol) and *N*-thiocyanatosaccharin (**2**) (0.144 g, 0.600 mmol). The reaction mixture was stirred at 40 °C for 0.5 h. Purification by flash column chromatography (10% ethyl acetate in hexane) gave 2-methyl-4-thiocyanatoanisole (**5d**) (0.0753 g, 84%) as a colorless oil. Spectroscopic data were consistent with the literature.^8^^1^H NMR (CDCl_3_, 400 MHz): δ 7.37 (dd, 1H, *J* = 8.7, 2.5 Hz), 7.35–7.32 (m, 1H), 6.84 (d, 1H, *J* = 8.7 Hz), 3.84 (s, 3H), 2.22 (s, 3H); ^13^C{^1^H} NMR (CDCl_3_, 101 MHz): δ 159.6 (C), 134.3 (CH), 131.4 (CH), 129.5 (C), 113.1 (C), 112.0 (C), 111.4 (CH), 55.6 (CH_3_), 16.2 (CH_3_); MS (ESI) *m*/*z* 180 (M + H^+^, 100).

### 2,3-Dihydro-5-thiocyanatobenzofuran (**5e**)^30^

The reaction was performed according to the general procedure using 2,3-dihydrobenzofuran (**4e**) (0.0564 mL, 0.500 mmol) and *N*-thiocyanatosaccharin (**2**) (0.144 g, 0.600 mmol). The reaction mixture was stirred at 40 °C for 1.5 h. Purification by flash column chromatography (30% diethyl ether in hexane) gave 2,3-dihydro-5-thiocyanatobenzofuran (**5e**) (0.0831 g, 94%) as a colorless oil. Spectroscopic data were consistent with the literature.^30^^1^H NMR (CDCl_3_, 400 MHz): δ 7.43–7.40 (m, 1H), 7.32 (dd, 1H, *J* = 8.2, 1.8 Hz), 6.80 (d, 1H, *J* = 8.2 Hz), 4.63 (t, 2H, *J* = 8.8 Hz), 3.24 (t, 2H, *J* = 8.8 Hz); ^13^C{^1^H} NMR (CDCl_3_, 101 MHz): δ 162.4 (C), 133.3 (CH), 130.1 (C), 129.6 (CH), 113.1 (C), 112.1 (C), 111.1 (CH), 72.1 (CH_2_), 29.5 (CH_2_); MS (ESI) *m*/*z* 178 (M + H^+^, 100).

### 1-Methoxy-4-thiocyanatonaphthalene (**5f**)^31^

The reaction was performed according to the general procedure using 1-methoxynaphthalene (**4f**) (0.0726 mL, 0.500 mmol) and *N*-thiocyanatosaccharin (**2**) (0.144 g, 0.600 mmol). The reaction mixture was stirred at 40 °C for 0.25 h. Purification by flash column chromatography (10% ethyl acetate in hexane) gave 1-methoxy-4-thiocyanatonaphthalene (**5f**) (0.0987 g, 92%) as a white solid. Mp 97–100 °C (lit.^31^ 104–106 °C); ^1^H NMR (CDCl_3_, 400 MHz): δ 8.35 (d, 1H, *J* = 8.4 Hz), 8.29 (d, 1H, *J* = 8.4 Hz), 7.85 (d, 1H, *J* = 8.2 Hz), 7.75–7.70 (m, 1H), 7.63–7.57 (m, 1H), 6.81 (d, 1H, *J* = 8.2 Hz), 4.03 (s, 3H); ^13^C{^1^H} NMR (CDCl_3_, 101 MHz): δ 158.8 (C), 135.4 (CH), 133.8 (C), 128.8 (CH), 126.9 (C), 126.5 (CH), 124.7 (CH), 123.2 (CH), 111.6 (C), 110.5 (C), 104.2 (CH), 56.0 (CH_3_); MS (ESI) *m*/*z* 216 (M + H^+^, 100).

### 4-Thiocyanatophenol (**5g**)^18^

The reaction was performed according to the general procedure using phenol (**4g**) (0.0461 mg, 0.500 mmol) and *N*-thiocyanatosaccharin (**2**) (0.144 g, 0.600 mmol). The reaction mixture was stirred at 40 °C for 0.25 h. Purification by flash column chromatography (40% diethyl ether in hexane) gave 4-thiocyanatophenol (**5g**) (0.0669 g, 89%) as a yellow solid. Mp 53–55 °C (lit.^18^ 51–53 °C); ^1^H NMR (CDCl_3_, 400 MHz): δ 7.46–7.41 (m, 2H), 6.90–6.86 (m, 2H), 4.94 (br s, 1H); ^13^C{^1^H} NMR (CDCl_3_, 101 MHz): δ 158.2 (C), 134.4 (2 × CH), 117.6 (2 × CH), 113.3 (C), 112.4 (C); MS (ESI) *m*/*z* 152 (M + H^+^, 100).

### 3,5-Dimethyl-4-thiocyanatophenol (**5h**)^18^

The reaction was performed according to the general procedure using 3,5-dimethylphenol (**4h**) (0.0611 g, 0.500 mmol) and *N*-thiocyanatosaccharin (**2**) (0.144 g, 0.600 mmol). The reaction mixture was stirred at 40 °C for 0.25 h. Purification by flash column chromatography (30% ethyl acetate in hexane) gave 3,5-dimethyl-4-thiocyanatophenol (**5h**) (0.0741 g, 83%) as a yellow solid. Mp 115–117 °C. Spectroscopic data were consistent with the literature.^18^^1^H NMR (CDCl_3_, 400 MHz): δ 6.66 (s, 2H), 5.25 (s, 1H), 2.54 (s, 6H); ^13^C{^1^H} NMR (CDCl_3_, 101 MHz): δ 158.0 (C), 145.3 (2 × C), 116.3 (2 × CH), 113.1 (C), 111.5 (C), 22.3 (2 × CH_3_); MS (ESI) *m*/*z* 180 (M + H^+^, 100).

### Methyl 2-Hydroxy-5-thiocyanatobenzoate (**5i**)

The reaction was performed according to the general procedure using methyl salicylate (**4i**) (0.0432 mL, 0.333 mmol), iron(III) chloride (0.00540 g, 0.0333 mmol, 10 mol %), diphenyl selenide (0.00580 mL, 0.0333 mmol, 10 mol %), and *N*-thiocyanatosaccharin (**2**) (0.160 g, 0.666 mmol). The reaction mixture was stirred at 40 °C for 0.25 h. Purification by flash column chromatography (10% ethyl acetate in hexane) gave methyl 2-hydroxy-5-thiocyanatobenzoate (**5i**) (0.0490 g, 70%) as a white solid. Mp 75–77 °C; IR (neat) 3166, 2955, 2154, 1674, 1571, 1388, 1333, 1185, 750 cm^–1^; ^1^H NMR (CDCl_3_, 400 MHz): δ 11.02 (s, 1H), 8.10 (d, 1H, *J* = 2.5 Hz), 7.65 (d, 1H, *J* = 8.8, 2.5 Hz), 7.07 (d, 1H, *J* = 8.8 Hz), 4.00 (s, 3H); ^13^C{^1^H} NMR (CDCl_3_, 101 MHz): δ 169.4 (C), 163.3 (C), 139.3 (CH), 134.6 (CH), 120.3 (CH), 114.1 (C), 113.0 (C), 111.1 (C), 53.0 (CH_3_); MS (ESI) *m*/*z* 210 (M + H^+^, 100); HRMS (ESI) *m*/*z*: [M + H]^+^ calcd for C_9_H_7_NO_3_SH 210.0219; found 210.0220.

### 4-Thiocyanatoaniline (**5j**)^32^

The reaction was performed according to the general procedure using aniline (**4j**) (0.0456 mL, 0.500 mmol) and *N*-thiocyanatosaccharin (**2**) (0.144 g, 0.600 mmol). The reaction mixture was stirred at 40 °C for 5 min. Purification by flash column chromatography (30% ethyl acetate in hexane) gave 4-thiocyanatoaniline (**5j**) (0.0686 g, 91%) as a yellow solid. Mp 54–55 °C (lit.^32^ 50–52 °C); ^1^H NMR (CDCl_3_, 400 MHz): δ 7.37–7.33 (m, 2H), 6.68–6.65 (m, 2H), 3.97 (br s, 2H); ^13^C{^1^H} NMR (CDCl_3_, 101 MHz): δ 148.9 (C), 134.6 (2 × CH), 116.2 (2 × CH), 112.5 (C), 109.6 (C); MS (ESI) *m*/*z* 151 (M + H^+^, 100).

### 2-Methoxy-4-thiocyanato-5-methylaniline (**5k**)

The reaction was performed according to the general procedure using 2-methoxy-5-methylaniline (**4k**) (0.0457 g, 0.333 mmol) and *N*-thiocyanatosaccharin (**2**) (0.0961 g, 0.400 mmol). The reaction mixture was stirred at 0 °C for 5 min. Purification by flash column chromatography (25% ethyl acetate in hexane) gave 2-methoxy-4-thiocyanato-5-methylaniline (**5k**) (0.0564 g, 87%) as a brown solid. Mp 40–42 °C; IR (neat) 3297, 2916, 2148, 1618, 1575, 1503, 1260, 1217, 1031, 883 cm^–1^; ^1^H NMR (CDCl_3_, 400 MHz): δ 6.96 (s, 1H), 6.60 (s, 1H), 4.03 (br s, 2H), 3.85 (s, 3H), 2.40 (s, 3H); ^13^C{^1^H} NMR (CDCl_3_, 101 MHz): δ 145.9 (C), 139.4 (C), 135.2 (C), 116.7 (CH), 116.2 (CH), 112.1 (C), 108.0 (C), 56.0 (CH_3_), 20.2 (CH_3_); MS (ESI) *m*/*z* 195 (M + H^+^, 100); HRMS (ESI) *m*/*z*: [M + Na]^+^ calcd for C_9_H_10_N_2_OSH 195.0587; found 195.0587.

### 4-Thiocyanato-2-(trifluoromethyl)aniline (**5l**)^33^

The reaction was performed according to the general procedure using 2-(trifluoromethyl)aniline (**4l**) (0.0806 g, 0.500 mmol) and *N*-thiocyanatosaccharin (**2**) (0.144 g, 0.600 mmol). The reaction mixture was stirred at 40 °C for 1 h. Purification by flash column chromatography (25% ethyl acetate in hexane) gave 4-thiocyanato-2-(trifluoromethyl)aniline (**5l**) (0.0955 g, 88%) as an orange oil. Spectroscopic data were consistent with the literature.^33^^1^H NMR (CDCl_3_, 400 MHz): δ 7.66 (d, 1H, *J* = 2.3 Hz), 7.51 (dd, 1H, *J* = 8.7, 2.3 Hz), 6.78 (d, 1H, *J* = 8.7 Hz), 4.51 (br s, 2H); ^13^C{^1^H} NMR (CDCl_3_, 101 MHz): δ 146.7 (C), 137.6 (CH), 132.0 (CH, q, ^3^*J*_CF_ = 5.2 Hz), 124.0 (C, q, ^1^*J*_CF_ = 272.5 Hz), 118.7 (CH), 114.8 (C, q, ^2^*J*_CF_ = 31.3 Hz), 111.6 (C), 109.6 (C); MS (ESI) *m*/*z* 219 (M + H^+^, 100).

### 2-Fluoro-4-thiocyanatoaniline (**5m**)^34^

The reaction was performed according to the general procedure using 2-fluoroaniline (**4m**) (0.0321 mL, 0.333 mmol) and *N*-thiocyanatosaccharin (**2**) (0.0961 g, 0.400 mmol). The reaction mixture was stirred at 40 °C for 0.25 h. Purification by flash column chromatography (30% ethyl acetate in hexane) gave 2-fluoro-4-thiocyanatoaniline (**5m**) (0.0518 g, 92%) as a brown solid. Mp 32–34 °C (lit.^34^ 33–34 °C); ^1^H NMR (CDCl_3_, 400 MHz): δ 7.24 (dd, 1H, *J* = 10.4, 2.1 Hz), 7.17 (ddd, 1H, *J* = 8.4, 2.1, 1.0 Hz), 6.78 (dd, 1H, *J* = 9.0, 8.4 Hz), 4.04 (br s, 2H); ^13^C{^1^H} NMR (CDCl_3_, 101 MHz): δ 151.1 (C, d, ^1^*J*_CF_ = 244.4 Hz), 137.5 (C, d, ^2^*J*_CF_ = 12.5 Hz), 129.9 (CH, d, ^4^*J*_CF_ = 3.3 Hz), 119.9 (CH, d, ^2^*J*_CF_ = 20.8 Hz), 117.4 (CH, d, ^3^*J*_CF_ = 4.3 Hz), 111.7 (C), 109.7 (C, d, ^3^*J*_CF_ = 7.5 Hz); MS (ESI) *m*/*z* 169 (M + H^+^, 100).

### 2-Amino-5-thiocyanatobenzophenone (**5n**)

The reaction was performed according to the general procedure using 2-aminobenzophenone (**4n**) (0.0961 g, 0.500 mmol) and *N*-thiocyanatosaccharin (**2**) (0.144 g, 0.600 mmol). The reaction mixture was stirred at 40 °C for 0.25 h. Purification by flash column chromatography (10–25% ethyl acetate in hexane) gave 2-amino-5-thiocyanatobenzophenone (**5n**) (0.103 g, 81%) as a yellow solid. Mp 84–86 °C. IR (neat) 3332, 3029, 2152, 1635, 1572, 1249, 943, 829 cm^–1^; ^1^H NMR (CDCl_3_, 400 MHz): δ 7.71 (d, 1H, *J* = 2.3 Hz), 7.65–7.56 (m, 3H), 7.53–7.47 (m, 3H), 6.79 (d, 1H, *J* = 8.8 Hz), 6.45 (br s, 2H); ^13^C{^1^H} NMR (CDCl_3_, 101 MHz): δ 197.9 (C), 152.6 (C), 140.1 (CH), 139.0 (C), 138.5 (CH), 132.0 (CH), 129.2 (2 × CH), 128.6 (2 × CH), 119.1 (CH), 118.8 (C), 112.0 (C), 107.0 (C); MS (ESI) *m*/*z* 253 ([M – H]^–^, 100); HRMS (ESI) *m*/*z*: [M – H]^−^ calcd for C_14_H_9_N_2_OS 253.0440; found 253.0441.

### 2-Cyano-4-thiocyanatoaniline (**5o**)^34^

The reaction was performed according to the general procedure using 2-cyanoaniline (**4o**) (0.0393 g, 0.333 mmol) and *N*-thiocyanatosaccharin (**2**) (0.0961 g, 0.400 mmol). The reaction mixture was stirred at 40 °C for 0.5 h. Purification by flash column chromatography (30% ethyl acetate in hexane) gave 2-cyano-4-thiocyanatoaniline (**5o**) (0.0492 g, 84%) as a white solid. Mp 119–121 °C (lit.^34^ 126–127 °C); ^1^H NMR (CDCl_3_, 400 MHz): δ 7.63 (d, 1H, *J* = 2.3 Hz), 7.53 (dd, 1H, *J* = 8.8, 2.3 Hz), 6.80 (d, 1H, *J* = 8.8 Hz), 4.78 (br s, 2H); ^13^C{^1^H} NMR (CDCl_3_, 101 MHz): δ 151.3 (C), 138.4 (CH), 137.2 (CH), 116.9 (CH), 115.9 (C), 111.0 (C), 110.5 (C), 97.5 (C); MS (ESI) *m*/*z* 174 ([M – H]^−^, 100).

### Benzyl (4-Thiocyanatobenzene)carbamate (**5p**)^35^

The reaction was performed according to the general procedure using benzyl benzenecarbamate (**4p**) (0.114 g, 0.500 mmol), diphenyl selenide (0.00218 mL, 0.0125 mmol, 2.5 mol %), and *N*-thiocyanatosaccharin (**2**) (0.144 g, 0.600 mmol). The reaction mixture was stirred at 40 °C for 0.75 h. Purification by flash column chromatography (20% ethyl acetate in hexane) gave benzyl (4-thiocyanatobenzene)carbamate (**5p**) (0.123 g, 87%) as a white solid. Mp 75–77 °C (lit.^35^ 83–85 °C); ^1^H NMR (CDCl_3_, 400 MHz): δ 7.48 (br s, 4H), 7.43–7.32 (m, 5H), 6.85 (br s, 1H), 5.21 (s, 2H); ^13^C{^1^H} NMR (CDCl_3_, 101 MHz): δ 153.0 (C), 139.9 (C), 135.7 (C), 132.6 (2 × CH), 128.8 (2 × CH), 128.7 (CH), 128.5 (2 × CH), 120.0 (2 × CH), 117.3 (C), 111.2 (C), 67.6 (CH_2_); MS (ESI) *m*/*z* 307 (M + Na^+^, 100).

### 3-Thiocyanatoindole (**5q**)^8^

The reaction was performed according to the general procedure using indole (**4q**) (0.0390 g, 0.333 mmol) and *N*-thiocyanatosaccharin (**2**) (0.0961 g, 0.400 mmol). The reaction mixture was stirred at 40 °C for 5 min. Purification by flash column chromatography (30% ethyl acetate in hexane) gave 3-thiocyanatoindole (**5q**) (0.0540 g, 93%) as a brown solid. Mp 65–67 °C (lit.^8^ 72–74 °C); ^1^H NMR (CDCl_3_, 400 MHz): δ 8.65 (br s, 1H), 7.84–7.79 (m, 1H), 7.51 (d, 1H, *J* = 2.8 Hz), 7.47–7.40 (m, 1H), 7.36–7.28 (m, 2H); ^13^C{^1^H} NMR (CDCl_3_, 101 MHz): δ 136.1 (C), 131.1 (CH), 127.8 (C), 124.1 (CH), 122.1 (CH), 118.9 (CH), 112.2 (CH), 112.0 (C), 92.5 (C); MS (ESI) *m*/*z* 173 ([M – H]^−^, 100).

### 3-Thiocyanato-5-nitroindole (**5r**)^29^

The reaction was performed according to the general procedure using 5-nitroindole (**4r**) (0.0540 g, 0.333 mmol) and *N*-thiocyanatosaccharin (**2**) (0.0961 g, 0.400 mmol). The reaction mixture was stirred at 40 °C for 10 min. Purification by flash column chromatography (40% ethyl acetate in hexane) gave 3-thiocyanato-5-nitroindole (**5r**) (0.0626 g, 86%) as a pale yellow solid. Mp 210–212 °C (lit.^29^ 207–209 °C); ^1^H NMR (DMSO-*d*_6_, 400 MHz): δ 8.55 (d, 1H, *J* = 2.3 Hz), 8.29 (s, 1H), 8.15 (dd, 1H, *J* = 9.0, 2.3 Hz), 7.73 (d, 1H, *J* = 9.0 Hz); ^13^C{^1^H} NMR (DMSO-*d*_6_, 101 MHz): δ 142.2 (C), 139.5 (C), 137.1 (CH), 126.9 (C), 118.2 (CH), 114.4 (CH), 113.7 (CH), 111.9 (C), 93.2 (C); MS (ESI) *m*/*z* 218 ([M – H]^−^, 100).

### 3-Thiocyanato-7-azaindole (**5s**)^8^

The reaction was performed according to the general procedure using 7-azaindole (**4s**) (0.0393 g, 0.333 mmol) and *N*-thiocyanatosaccharin (**2**) (0.0961 g, 0.400 mmol). The reaction mixture was stirred at 40 °C for 5 min. Purification by flash column chromatography (40% ethyl acetate in hexane) gave 3-thiocyanato-7-azaindole (**5s**) (0.0537 g, 92%) as a white solid. Mp 203–206 °C (lit.^8^ 197–199 °C); ^1^H NMR (DMSO-*d*_6_, 400 MHz): δ 12.60 (br s, 1H), 8.39 (d, 1H, *J* = 4.7, 1.6 Hz), 8.17 (d, 1H, *J* = 2.3 Hz), 8.12 (dd, 1H, *J* = 7.9, 1.6 Hz), 7.30 (dd, 1H, *J* = 7.9, 4.7 Hz); ^13^C{^1^H} NMR (DMSO-*d*_6_, 101 MHz): δ 148.4 (C), 144.5 (CH), 134.0 (CH), 126.5 (CH), 119.8 (C), 117.4 (CH), 112.1 (C), 89.0 (C); MS (ESI) *m*/*z* 176 (M + H^+^, 100).

### *N*-Phenylsulfonyl-3-thiocyanatoindole (**5t**)

The reaction was performed according to the general procedure using 1-(phenylsulfonyl)indole (**4t**) (0.129 g, 0.500 mmol) and *N*-thiocyanatosaccharin (**2**) (0.144 g, 0.600 mmol). The reaction mixture was at 40 °C for 10 min. Purification by flash column chromatography (10% ethyl acetate in petroleum ether) gave *N*-phenylsulfonyl-3-thiocyanatoindole (**5t**) (0.0973 g, 62%) as a white solid. Mp 133–135 °C; IR (neat) 3032, 2150, 1582, 1447, 1370, 1269, 1173, 1130, 999 cm^–1^; ^1^H NMR (CDCl_3_, 400 MHz): δ 8.02 (d, 1H, *J* = 8.4 Hz), 7.97–7.93 (m, 3H), 7.74 (d, 1H, *J* = 7.6 Hz), 7.64–7.58 (m, 1H), 7.54–7.38 (m, 4H); ^13^C{^1^H} NMR (CDCl_3_, 101 MHz): δ 137.5 (C), 134.9 (CH), 134.8 (C), 131.2 (CH), 129.8 (2 × CH), 129.3 (C), 127.2 (2 × CH), 126.5 (CH), 124.7 (CH), 119.8 (CH), 113.9 (CH), 109.6 (C), 101.2 (C); MS (ESI) *m*/*z* 315 (M + H^+^, 100); HRMS (ESI) *m*/*z*: [M + H]^+^ calcd for C_15_H_10_N_2_O_2_S_2_H 315.0256; found 315.0259.

### 2,4,6-Trimethylthiocyanatobenzene (**5u**)^5c^

The reaction was performed according to the general procedure using mesitylene (**4u**) (0.0601 g, 0.500 mmol) and *N*-thiocyanatosaccharin (**2**) (0.144 g, 0.600 mmol). The reaction mixture was stirred for at 40 °C for 0.5 h. Purification by flash column chromatography (10% ethyl acetate in petroleum ether) gave 2,4,6-trimethylthiocyanatobenzene (**5u**) (0.0822 g, 93%) as a white solid. Mp 58–62 °C (lit.^5c^ 62 °C); ^1^H NMR (CDCl_3_, 400 MHz): δ 7.01 (s, 2H), 2.56 (s, 6H), 2.30 (s, 3H); ^13^C{^1^H} NMR (CDCl_3_, 101 MHz): δ 142.8 (2 × C), 141.6 (C), 130.2 (2 × CH), 119.2 (C), 111.0 (C), 22.0 (2 × CH_3_), 21.2 (CH_3_); MS (ESI) *m*/*z* 178 (M + H^+^, 100).

### 1,3-Dimethyl-4-thiocyanatobenzene (**5v**)^4e^

The reaction was performed according to the general procedure using *m*-xylene (**4v**) (0.0612 mL, 0.500 mmol), iron(III) chloride (0.00811 g, 0.0500 mmol, 10 mol %), and *N*-thiocyanatosaccharin (**2**) (0.144 g, 0.600 mmol). The reaction mixture was stirred at 40 °C for 2 h. Purification by flash column chromatography (30% dichloromethane in hexane) gave 1,3-dimethyl-4-thiocyanatobenzene (**5v**) (0.0715 g, 88%) as a colorless oil. Spectroscopic data were consistent with the literature.^4e^^1^H NMR (CDCl_3_, 400 MHz): δ 7.50 (d, 1H, *J* = 8.0 Hz), 7.14–7.12 (m, 1H), 7.09–7.04 (m, 1H), 2.46 (s, 3H), 2.34 (s, 3H); ^13^C{^1^H} NMR (CDCl_3_, 101 MHz): δ 141.1 (C), 139.9 (C), 132.9 (CH), 132.4 (CH), 128.6 (CH), 119.9 (C), 111.1 (C), 21.2 (CH_3_), 20.6 (CH_3_); MS (ESI) *m*/*z* 164 (M + H^+^, 100).

### 5-[(3′,5′-Dimethyl-4′-thiocyanatophenoxy)methyl]-1,3-oxazolidin-2-one (**7**)

The reaction was performed according to the general procedure using metaxalone (**6**) (0.0737 g, 0.333 mmol) and *N*-thiocyanatosaccharin (**2**) (0.112 g, 0.466 mmol). The reaction mixture was stirred at 40 °C for 0.5 h. Purification by flash column chromatography (80–90% ethyl acetate in hexane) gave 5-[(3′,5′-dimethyl-4′-thiocyanatophenoxy)methyl]-1,3-oxazolidin-2-one (**7**) (0.0668 g, 72%) as a white solid. Mp 174–176 °C; IR (neat) 3246, 2959, 2148, 1747, 1583, 1309, 1244, 1168, 1074, 856 cm^–1^; ^1^H NMR (CDCl_3_, 400 MHz): δ 6.74 (s, 2H), 5.67 (br s, 1H), 5.01–4.93 (m, 1H), 4.15 (d, 2H, *J* = 4.7 Hz), 3.78 (t, 1H, *J* = 8.7 Hz), 3.60 (dd, 1H, *J* = 8.7, 6.1 Hz), 2.57 (s, 6H); ^13^C{^1^H} NMR (CDCl_3_, 101 MHz): δ 160.0 (C), 159.2 (C), 145.1 (2 × C), 115.4 (2 × CH), 114.4 (C), 111.0 (C), 74.0 (CH), 68.1 (CH_2_), 42.7 (CH_2_), 22.5 (2 × CH_3_); MS (ESI) *m*/*z* 279 (M + H^+^, 100); HRMS (ESI) *m*/*z*: [M + H]^+^ calcd for C_13_H_14_N_2_O_3_SH 279.0798; found 279.0802.

### 2-Thiocyanato-β-estradiol dibenzyl ether (**9**)

The reaction was performed according to the general procedure using β-estradiol dibenzyl ether (**8**) (0.0453 g, 0.100 mmol), diphenyl selenide (0.440 mL, 0.00250 mmol, 2.5 mol %), and *N*-thiocyanatosaccharin (**2**) (0.0288 g, 0.120 mmol). The reaction mixture was stirred at 0 °C for 10 min. Purification by flash column chromatography (60% dichloromethane in hexane) gave 2-thiocyanato-β-estradiol dibenzyl ether (**9**) (0.0439 g, 86%) as a white solid. Mp 123–125 °C; IR (neat) 2926, 2153, 1596, 1496, 1307, 1258, 1078, 738 cm^–1^; [α]_D_^23^ +63.3 (*c* 0.1, CHCl_3_); ^1^H NMR (CDCl_3_, 400 MHz): δ 7.49–7.25 (m, 11H), 6.71 (s, 1H), 5.13 (s, 2H), 4.58 (s, 2H), 3.51 (t, 1H, *J* = 8.2 Hz), 2.90–2.78 (m, 2H), 2.35–2.26 (m, 1H), 2.22–2.00 (m, 3H), 1.92–1.84 (m, 1H), 1.73–1.14 (m, 8H), 0.88 (s, 3H); ^13^C{^1^H} NMR (CDCl_3_, 101 MHz): δ 154.0 (C), 140.4 (C), 139.4 (C), 136.2 (C), 135.1 (C), 128.8 (2 × CH), 128.4 (2 × CH), 128.33 (CH), 128.32 (CH), 127.5 (3 × CH), 127.3 (2 × CH), 113.5 (CH), 111.3 (C), 109.9 (C), 88.3 (CH), 71.9 (CH_2_), 71.1 (CH_2_), 50.3 (CH), 44.1 (CH), 43.5 (C), 38.4 (CH), 37.9 (CH_2_), 29.9 (CH_2_), 28.2 (CH_2_), 27.0 (CH_2_), 26.5 (CH_2_), 23.3 (CH_2_), 11.9 (CH_3_); MS (ESI) *m*/*z* 510 (M + H^+^, 100); HRMS (ESI) *m*/*z*: [M + H]^+^ calcd for C_33_H_35_NO_2_SH 510.2461; found 510.2464.

### 1-(2′-Methyl-4′-nitrophenoxy)-4-thiocyanatobenzene (**11**)

The reaction was performed according to the general procedure using 2-methyl-4-nitro-1-phenoxybenzene (**10**) (0.0763 g, 0.333 mmol), iron(III) chloride (0.00540 g, 0.0333 mmol, 10 mol%), diphenyl selenide (0.00580 mL, 0.0333 mmol, 10 mol%), and *N*-thiocyanatosaccharin (**2**) (0.176 g, 0.733 mmol). The reaction mixture was stirred at 40 °C for 0.25 h. Purification by flash column chromatography (5% diethyl ether in hexane) gave 1-(2′-methyl-4′-nitrophenoxy)-4-thiocyanatobenzene (**11**) (0.0863 g, 91%) as a white solid. Mp 62–64 °C; IR (neat) 2925, 2156, 1581, 1486, 1340, 1244, 1091, 843 cm^–1^; ^1^H NMR (CDCl_3_, 400 MHz): δ 8.18 (d, 1H, *J* = 2.7 Hz), 8.05 (dd, 1H, *J* = 9.0, 2.7 Hz), 7.61–7.56 (m, 2H), 7.09–7.04 (m, 2H), 6.90 (d, 1H, *J* = 9.0 Hz), 2.37 (s, 3H); ^13^C{^1^H} NMR (CDCl_3_, 101 MHz): δ 159.6 (C), 157.6 (C), 143.8 (C), 133.4 (2 × CH), 130.7 (C), 127.2 (CH), 123.4 (CH), 120.7 (2 × CH), 119.1 (C), 117.9 (CH), 110.8 (C), 16.5 (CH_3_); MS (ESI) *m*/*z* 287 (M + H^+^, 100); HRMS (ESI) *m*/*z*: [M + H]^+^ calcd for C_14_H_10_N_2_O_3_SH 287.0485; found 287.0484.

### 1-(2′-Methyl-4′-nitrophenoxy)-4-(trifluoromethylsulfanyl)benzene (**12**)^37^

To a suspension of 1-(2′-methyl-4′-nitrophenoxy)-4-thiocyanatobenzene (**11**) (0.106 g, 0.370 mmol) and cesium carbonate (0.241 g, 0.740 mmol) in dry acetonitrile (0.7 mL) under argon was added trimethyl(trifluoromethyl)silane (0.109 mL, 0.740 mmol), and the reaction mixture was stirred at room temperature for 3 h. The reaction mixture was diluted with ethyl acetate (10 mL) and washed with water (10 mL). The aqueous layer was extracted with ethyl acetate (2 × 10 mL), and the combined organic layers were washed with brine (30 ml). The organic phase was dried (MgSO_4_), filtered, and concentrated *in vacuo*. Purification by flash column chromatography (3% ethyl acetate in hexane) gave 1-(2′-methyl-4′-nitrophenoxy)-4-(trifluoromethylsulfanyl)benzene (**12**) (0.0942 g, 77%) as a colorless oil. Spectroscopic data were consistent with the literature.^37^^1^H NMR (CDCl_3_, 400 MHz): δ 8.19 (d, 1H, *J* = 2.8 Hz), 8.06 (dd, 1H, *J* = 8.9, 2.8 Hz), 7.70–7.65 (m, 2H), 7.06–7.01 (m, 2H), 6.93 (d, 1H, *J* = 8.9 Hz), 2.38 (s, 3H); ^13^C{^1^H} NMR (CDCl_3_, 101 MHz): δ 159.6 (C), 158.5 (C), 143.9 (C), 138.8 (2 × CH), 130.9 (C), 129.6 (C, q, ^1^*J*_CF_ = 308.4 Hz), 127.2 (CH), 123.4 (CH), 119.7 (2 × CH), 119.6 (C, q, ^3^*J*_CF_ = 2.5 Hz), 118.2 (CH), 16.5 (CH_3_); MS (ESI) *m*/*z* 330 (M + H^+^, 100).

### 2-Bromo-4-thiocyanatoanisole (**14**)

Iron(III) chloride (0.0535 g, 0.330 mmol) was dissolved in 1-butyl-3-methylimidazolium bis(trifluoromethanesulfonyl)imide (0.290 mL, 0.990 mmol) and stirred for 0.5 h at room temperature and then added to a solution of *N*-thiocyanatosaccharin (**2**) (0.951 g, 3.96 mmol) in dry dichloromethane (20 mL) under argon. Anisole (**4a**) (0.357 mL, 3.30 mmol) was added, and the reaction mixture was stirred in the dark at 40 °C for 1 h. The reaction mixture was then cooled to room temperature, and *N*-bromosuccinimide (0.704 g, 3.96 mmol) was added. The reaction mixture was stirred at 40 °C for 18 h. After cooling to room temperature, the reaction mixture was diluted with dichloromethane (20 mL) and washed with water (20 mL). The aqueous layer was extracted with dichloromethane (2 × 20 mL), and the combined organic layers were washed with brine (50 mL). The organic phase was dried (MgSO_4_), filtered, and concentrated *in vacuo*. Purification by flash column chromatography (7.5–10% ethyl acetate in hexane) gave 2-bromo-4-thiocyanatoanisole (**14**) (0.701 g, 87%) as a white solid. Mp 61–62 °C; IR (neat) 2944, 2152, 1577, 1483, 1438, 1256, 1010, 810 cm^–1^; ^1^H NMR (CDCl_3_, 400 MHz): δ 7.77 (d, 1H, *J* = 2.4 Hz), 7.51 (dd, 1H, *J* = 8.7, 2.4 Hz), 6.94 (d, 1H, *J* = 8.7 Hz), 3.93 (s, 3H); ^13^C{^1^H} NMR (CDCl_3_, 101 MHz): δ 157.9 (C), 136.6 (CH), 132.7 (CH), 115.2 (C), 113.3 (C), 113.1 (CH), 111.0 (C), 56.7 (CH_3_); MS (ESI) *m*/*z* 244 (M + H^+^, 100); HRMS (ESI) *m*/*z*: [M + H]^+^ calcd for C_8_H_6_^79^BrNOSH 243.9426; found 243.9427.

### 2-Bromo-4-(trifluoromethylsulfanyl)anisole (**15**)^38^

To a suspension of 2-bromo-4-thiocyanatoanisole (**14**) (0.610 g, 2.50 mmol) and cesium carbonate (1.63 g, 5.00 mmol) in dry acetonitrile (5 mL) under argon was added trimethyl(trifluoromethyl)silane (0.739 mL, 5.00 mmol), and the reaction mixture was stirred at room temperature for 4 h. The reaction mixture was diluted with ethyl acetate (30 mL) and washed with water (30 mL). The aqueous layer was extracted with ethyl acetate (2 × 30 mL), and the combined organic layers were washed with brine (50 mL). The organic phase was dried (MgSO_4_), filtered, and concentrated *in vacuo*. Purification by flash column chromatography (7.5% ethyl acetate in hexane) gave 2-bromo-4-(trifluoromethylsulfanyl)anisole (**15**) (0.513 g, 71%) as a colorless oil. Spectroscopic data were consistent with the literature.^38^^1^H NMR (CDCl_3_, 400 MHz): δ 7.84 (d, 1H, *J* = 2.2 Hz), 7.58 (dd, 1H, *J* = 8.6, 2.2 Hz), 6.92 (d, 1H, *J* = 8.6 Hz), 3.93 (s, 3H); ^13^C{^1^H} NMR (CDCl_3_, 101 MHz): δ 158.4 (C), 141.1 (CH), 137.4 (CH), 129.5 (C, q, ^1^*J*_CF_ = 308.5 Hz), 116.2 (C, q, ^3^*J*_CF_ = 2.4 Hz), 112.39 (C), 112.36 (C), 56.6 (CH_3_); MS (ESI) *m*/*z* 286 (M^+^, 100).

### 2-(4′-Nitrophenyl)-4-(trifluoromethylsulfanyl)anisole (**16**)

To a solution of 2-bromo-4-(trifluoromethylsulfanyl)anisole (**15**) (0.0851 g, 0.296 mmol), 4-nitrophenylboronic acid (0.0743 g, 0.445 mmol) and potassium phosphate tribasic (0.126 g, 0.593 mmol) in degassed tetrahydrofuran (0.7 mL) and water (1.3 mL) was added XPhos Pd G2 (0.00467 g, 0.00593 mmol), and the reaction mixture was stirred at 40 °C for 4 h. After cooling to ambient temperature, the reaction mixture was filtered through a short pad of Celite and washed with ethyl acetate (10 mL). The filtrate was washed with water (10 mL), and the aqueous layer was extracted with ethyl acetate (2 × 10 mL). The combined organic layers were washed with brine (20 mL), dried (MgSO_4_), filtered, and concentrated *in vacuo*. Purification by flash column chromatography (5% ethyl acetate in hexane) gave 2-(4′-nitrophenyl)-4-(trifluoromethylsulfanyl)anisole (**16**) as a brown oil (0.0841 g, 86%). IR (neat) 2950, 1595, 1509, 1345, 1267, 1099, 857 cm^–1^; ^1^H NMR (CDCl_3_, 400 MHz): δ 8.30–8.26 (m, 2H), 7.72–7.65 (m, 3H), 7.62 (d, 1H, *J* = 2.4 Hz), 7.06 (d, 1H, *J* = 8.6 Hz), 3.88 (s, 3H); ^13^C{^1^H} NMR (CDCl_3_, 101 MHz): *δ* 158.8 (C), 147.2 (C), 143.8 (C), 138.9 (CH), 138.8 (CH), 130.5 (2 × CH), 129.8 (C), 129.6 (C, q, ^1^*J*_CF_ = 308.1 Hz), 123.5 (2 × CH), 115.8 (C, q, ^3^*J*_CF_ = 2.3 Hz), 112.4 (CH), 56.0 (CH_3_); MS (ESI) *m*/*z* 330 (M + H^+^, 100); HRMS (ESI) *m*/*z*: [M + H]^+^ calcd for C_14_H_10_F_3_NO_3_SH 330.0406; found 330.0405.

### Methyl (2*E*)-3-(2′-Methoxy-5′-trifluoromethylsulfanylbenzene)acrylate (17)

To a solution of 2-bromo-4-(trifluoromethylsulfanyl)anisole (**15**) (0.0956 g, 0.333 mmol) in degassed dimethylformamide (4 mL) was added methyl acrylate (0.0750 mL, 0.833 mmol) and *N,N*-diisopropylethylamine (0.174 mL, 1.00 mmol), followed by bis(triphenylphosphine)palladium(II) dichloride (0.00234 g, 0.0333 mmol), and the reaction mixture was stirred under argon at 100 °C for 18 h and at then 120 °C for 24 h. After cooling to ambient temperature, the reaction mixture was diluted with ethyl acetate (25 mL) and washed with water (3 × 25 mL) and brine (25 mL). The organic layer was dried (MgSO_4_), filtered, and concentrated *in vacuo*. Purification by flash column chromatography (10% ethyl acetate in hexane) gave methyl (2*E*)-3-(2′-methoxy-5′-trifluoromethylsulfanylbenzene)acrylate (**17**) as a white solid (0.0657 g, 68%). Mp 67–68 °C; IR (neat) 2945, 1711, 1586, 1484, 1438, 1253, 1093, 814 cm^–1^; ^1^H NMR (CDCl_3_, 400 MHz): δ 7.92 (d, 1H, *J* = 16.2 Hz), 7.77 (d, 1H, *J* = 2.3 Hz), 7.62 (dd, 1H, *J* = 8.7, 2.3 Hz), 6.95 (d, 1H, *J* = 8.7 Hz), 6.55 (d, 1H, *J* = 16.2 Hz), 3.93 (s, 3H), 3.81 (s, 3H); ^13^C{^1^H} NMR (CDCl_3_, 101 MHz): δ 167.6 (C), 160.3 (C), 139.7 (CH), 138.8 (CH), 137.3 (CH), 129.6 (C, q, ^1^*J*_CF_ = 308.4 Hz), 125.0 (C), 120.2 (CH), 115.6 (C, q, ^3^*J*_CF_ = 2.3 Hz), 112.3 (CH), 56.0 (CH_3_), 51.9 (CH_3_); MS (ESI) *m*/*z* 293 (M + H^+^, 100); HRMS (ESI) *m*/*z*: [M + H]^+^ calcd for C_12_H_11_F_3_O_3_SH 293.0454; found 293.0459.

### 2-(Phenylethynyl)-4-(trifluoromethylsulfanyl)anisole (**18**)

A reaction vial was charged with 2-bromo-4-(trifluoromethylsulfanyl)anisole (**15**) (0.0956 g, 0.333 mmol), tetrakis(triphenylphosphine)palladium(0) (0.0192 g, 0.0167 mmol), and copper(I) bromide (0.00478 g, 0.0333 mmol). Degassed tetrahydrofuran (0.7 mL) was added, and the solution was stirred for 0.1 h. Triethylamine (0.560 mL, 4.00 mmol) and phenylacetylene (0.0439 mL, 0.400 mmol) were added, and the reaction mixture was stirred at 75 °C under argon for 3 h. The reaction mixture was cooled to ambient temperature, diluted with water (10 mL), and extracted with ethyl acetate (3 × 10 mL). The combined organic layers were washed with brine (20 mL), dried (MgSO_4_), filtered, and concentrated *in vacuo*. Purification by flash column chromatography (5% ethyl acetate in hexane) gave 2-(phenylethynyl)-4-(trifluoromethylsulfanyl)anisole (**18**) as a white solid (0.0880 g, 86%). Mp 69–71 °C; IR (neat) 2919, 1585, 1479, 1250, 1091, 1015, 750 cm^–1^; ^1^H NMR (CDCl_3_, 400 MHz): δ 7.79 (d, 1H, *J* = 2.3 Hz), 7.61–7.54 (m, 3H), 7.39–7.34 (m, 3H), 6.93 (d, 1H, *J* = 8.7 Hz), 3.95 (s, 3H); ^13^C{^1^H} NMR (CDCl_3_, 101 MHz): δ 162.1 (C), 141.7 (CH), 138.4 (CH), 131.9 (2 × CH), 129.6 (C, q, ^1^*J*_CF_ = 308.1 Hz), 128.7 (CH), 128.5 (2 × CH), 123.1 (C), 115.1 (C, q, ^3^*J*_CF_ = 2.3 Hz), 114.4 (C), 111.7 (CH), 94.9 (C), 84.3 (C), 56.3 (CH_3_); MS (ESI) *m/z* 309 (M + H^+^, 100); HRMS (ESI) *m*/*z*: [M + H]^+^ calcd for C_16_H_11_F_3_OSH 309.0555; found 309.0556.

## Data Availability

The data underlying this study are available in the published article and its online Supporting Information.

## Supporting Information Available

^1^H and ^13^C NMR spectra of all compounds (PDF)
