# Supplementary material for: Regioselective C–H Thiocyanation of Arenes by Iron(III) Chloride Catalysis
Source: J Org Chem. 2023 May 9;88(11):7208–18. doi: 10.1021/acs.joc.3c00454 (PMC10242756; doi:10.1021/acs.joc.3c00454)

Supporting Information for:

Regioselective C–H Thiocyanation of Arenes by Iron(III) Chloride Catalysis

Lachlan J. N. Waddell, Maisie R. Senkans and Andrew Sutherland*

School of Chemistry, The Joseph Black Building, University of Glasgow, Glasgow G12 8QQ, UK. Email: Andrew.Sutherland@glasgow.ac.uk.

Table of Contents

1. ^{1}H and ^{13}C NMR Spectra for all Compounds S2–S63

1. ^{1}H and ^{13}C NMR Spectra for all compounds

^{1}H NMR (400 MHz, $CDCl_3$)

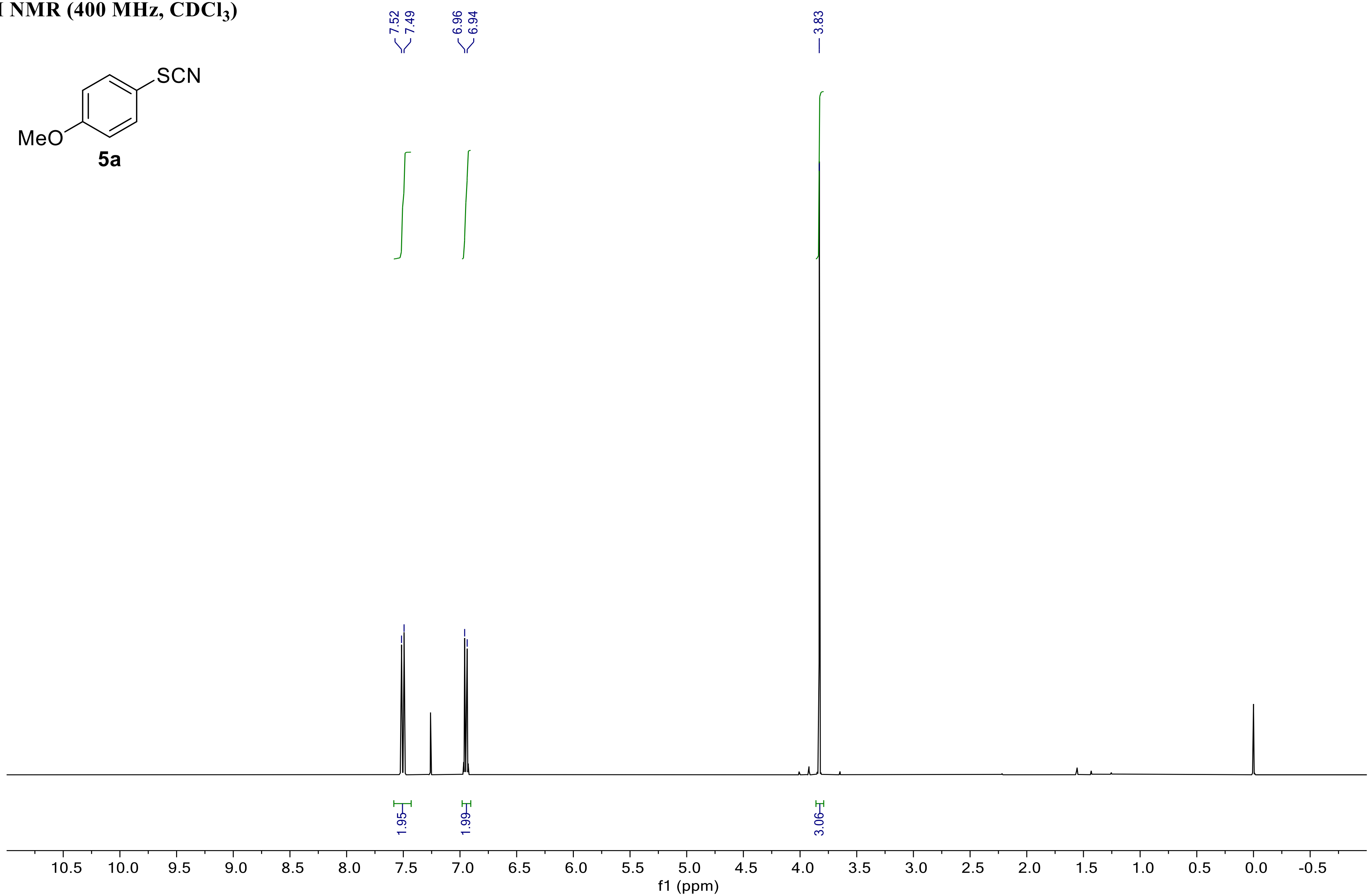

$^{13}C\{^{1}H\}$ NMR (101 MHz, $CDCl_3$)

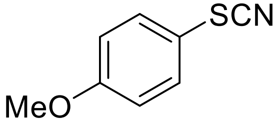

5a

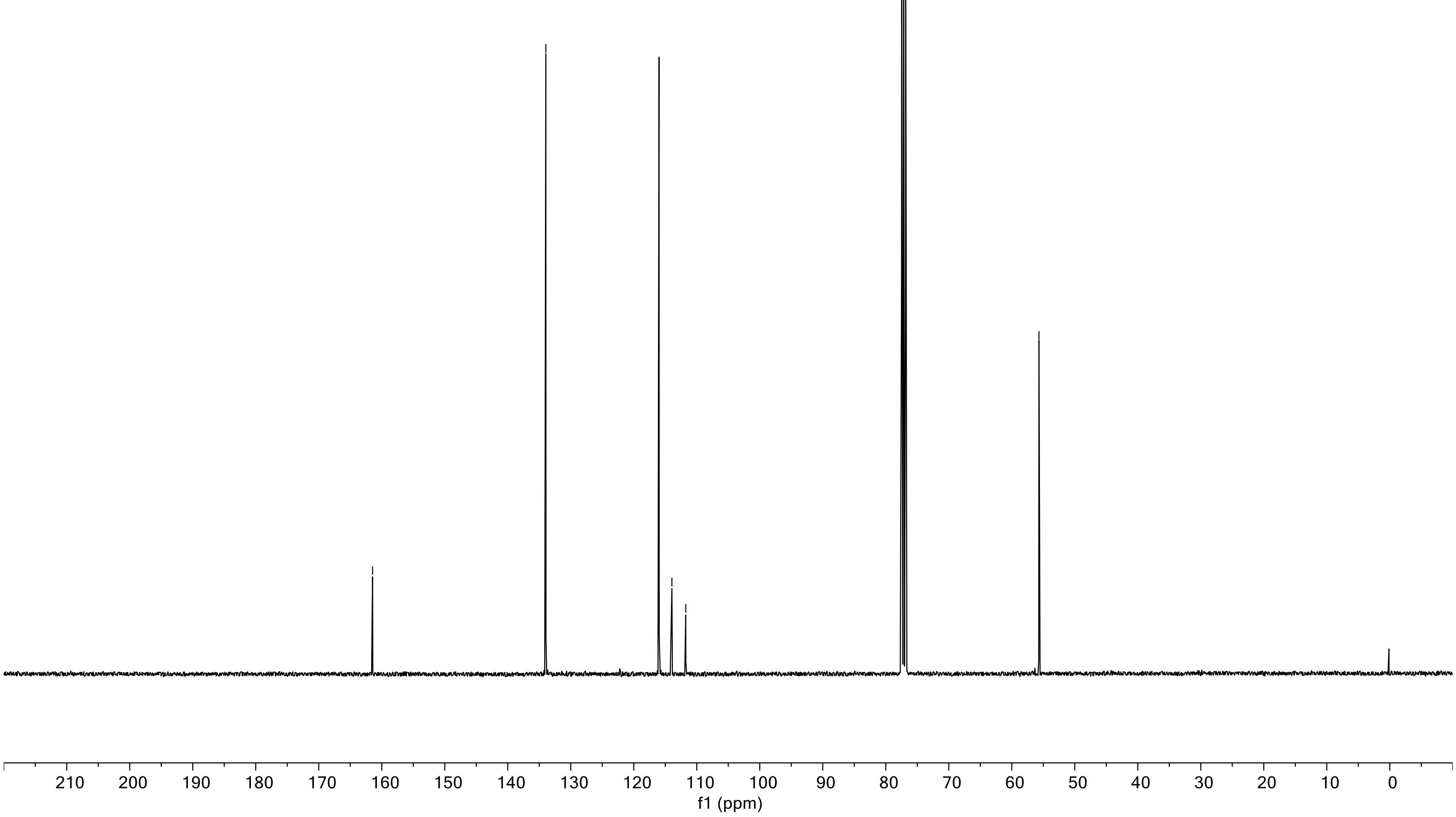

^{1}H NMR (500 MHz, $CDCl_3$)

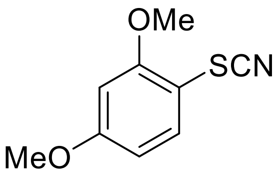

5b

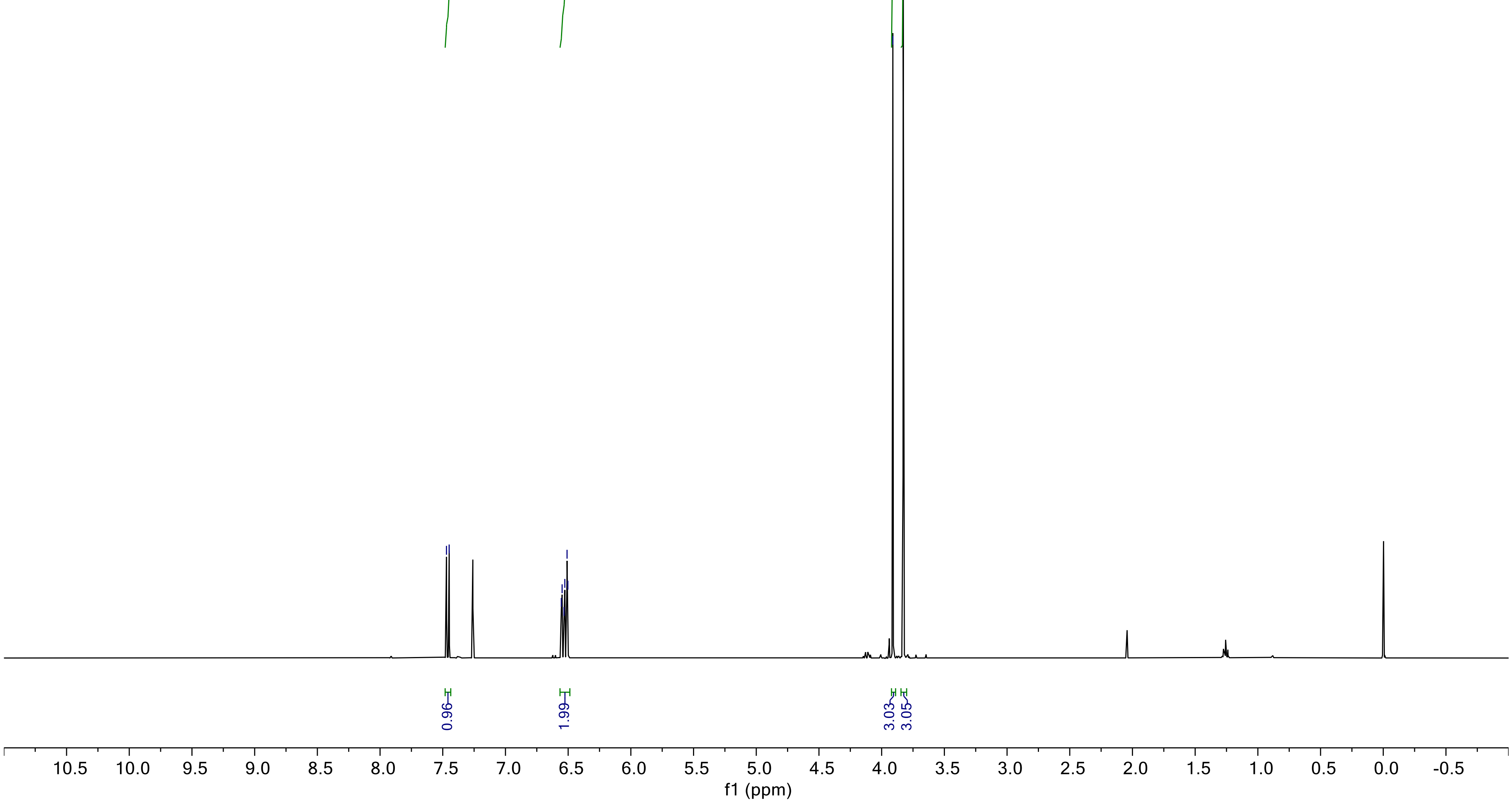

$^{13}C\{^{1}H\}$ NMR (126 MHz, $CDCl_3$)

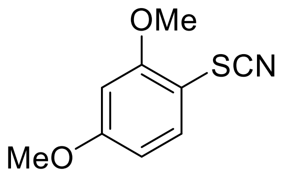

5b

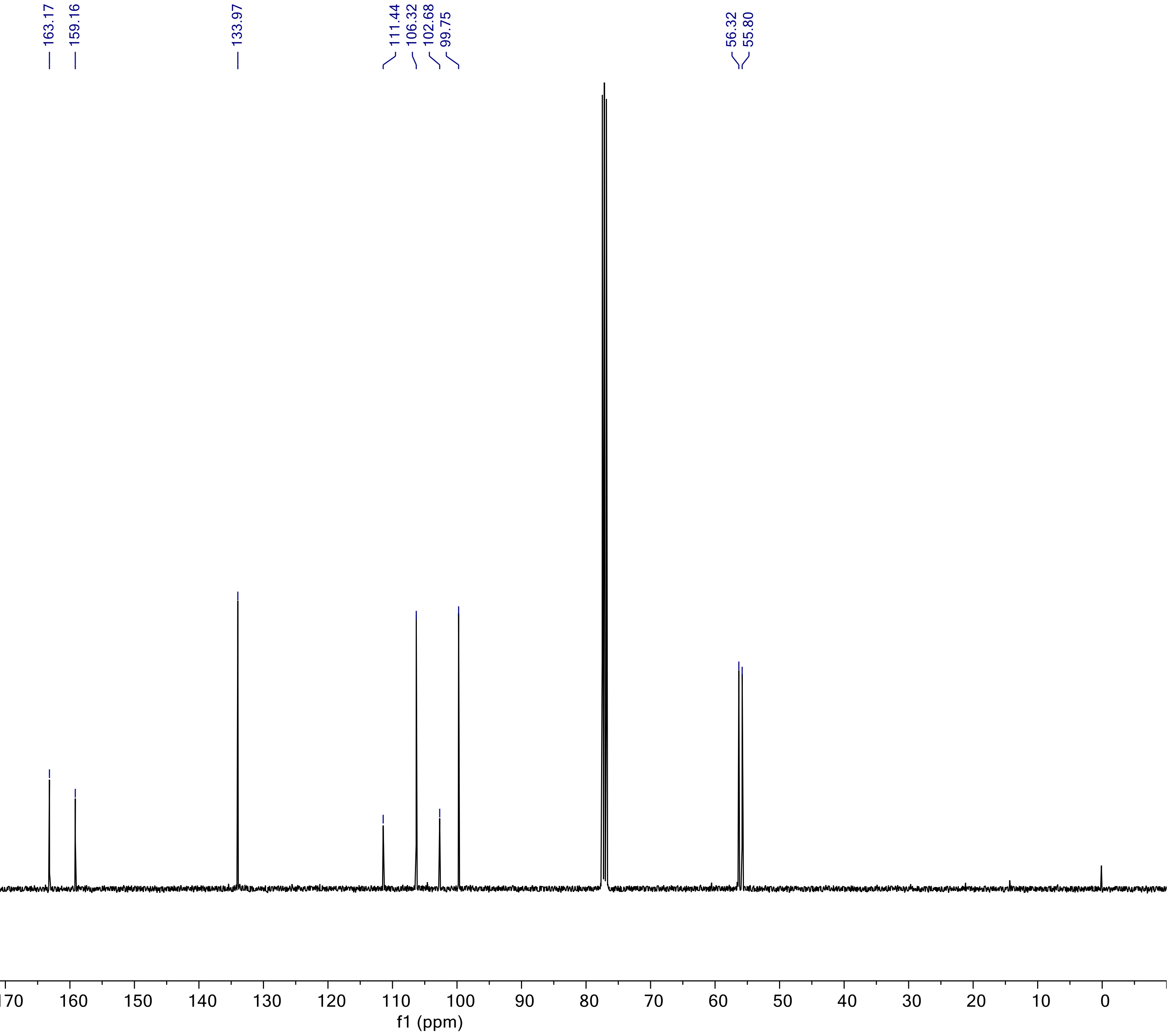

^{1}H NMR (400 MHz, $CDCl_3$)

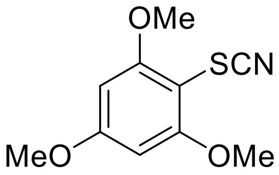

5c

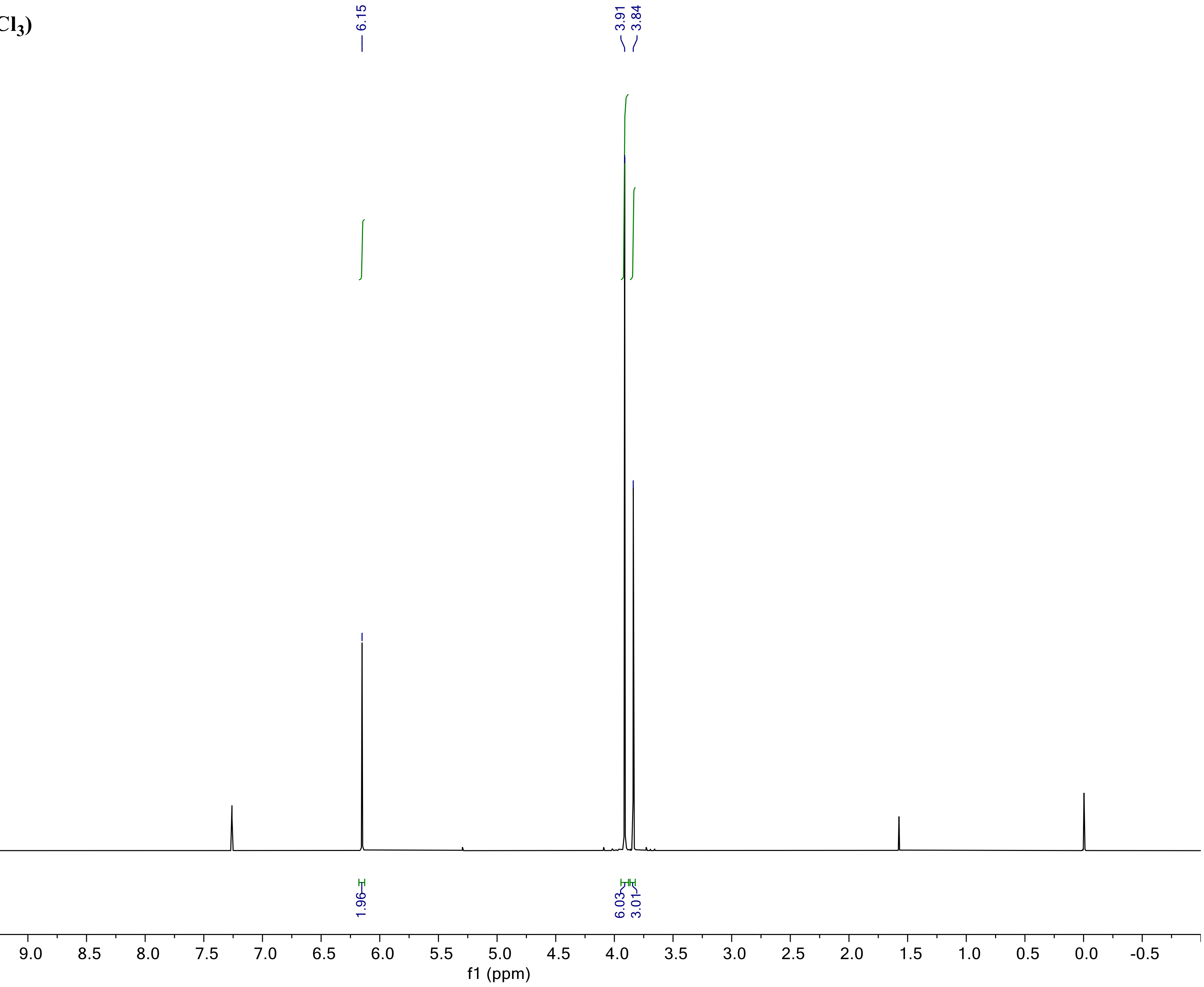

$^{13}C\{^{1}H\}$ NMR (101 MHz, $CDCl_3$)

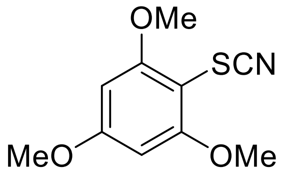

5c

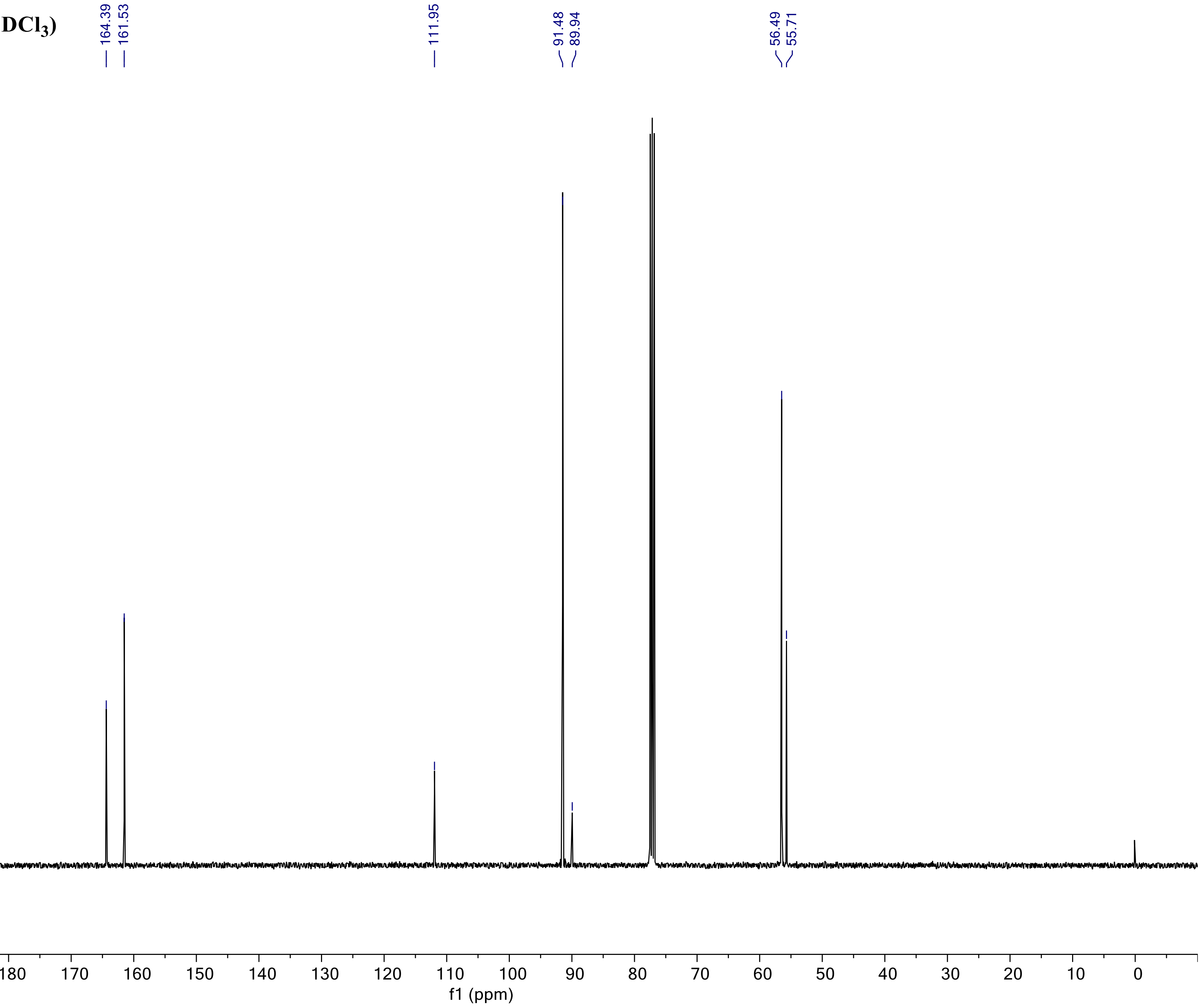

^{1}H NMR (400 MHz, $CDCl_3$)

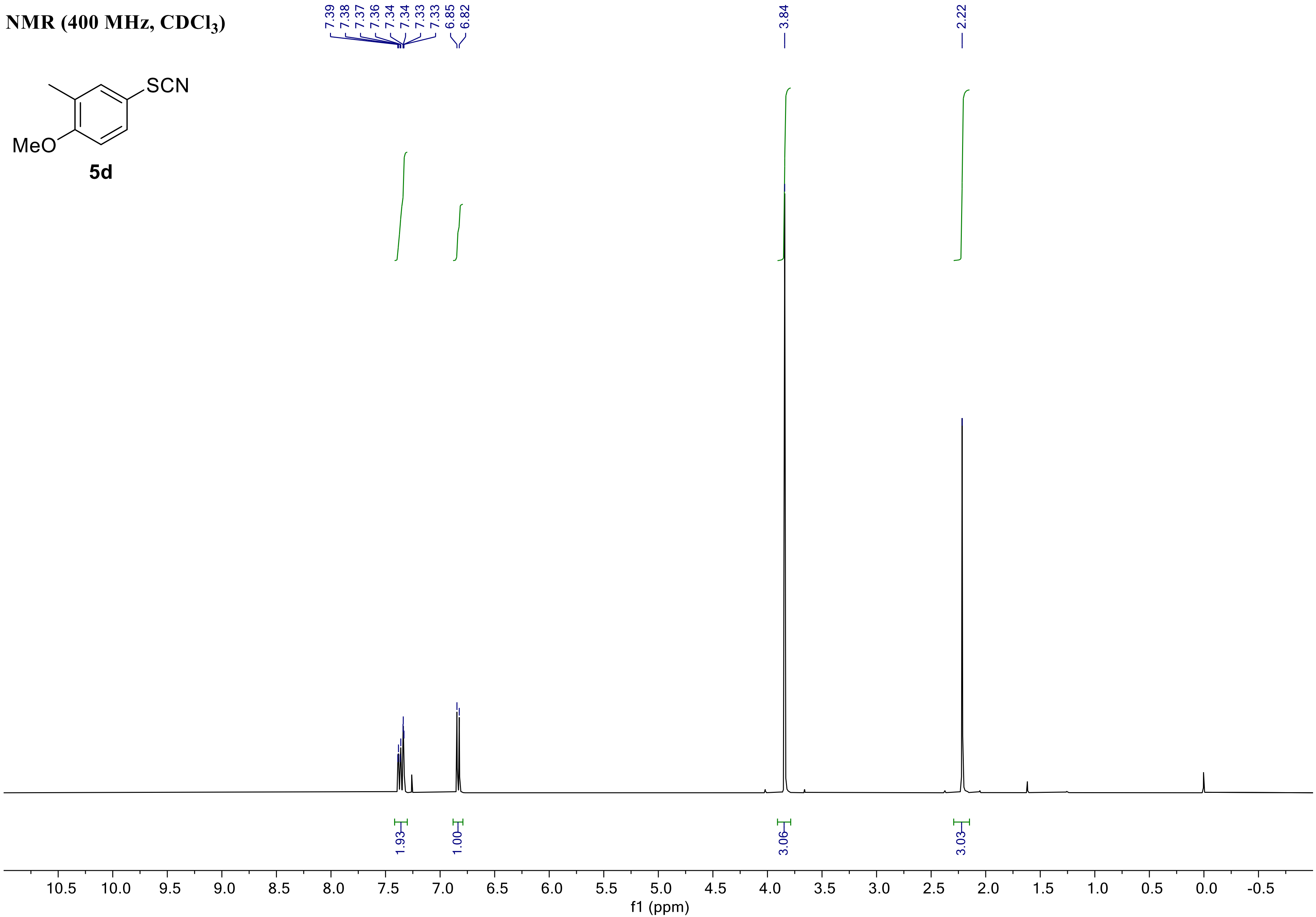

5d

$^{13}C\{^1H\}$ NMR (101 MHz, $CDCl_3$)

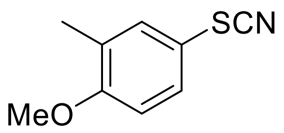

5d

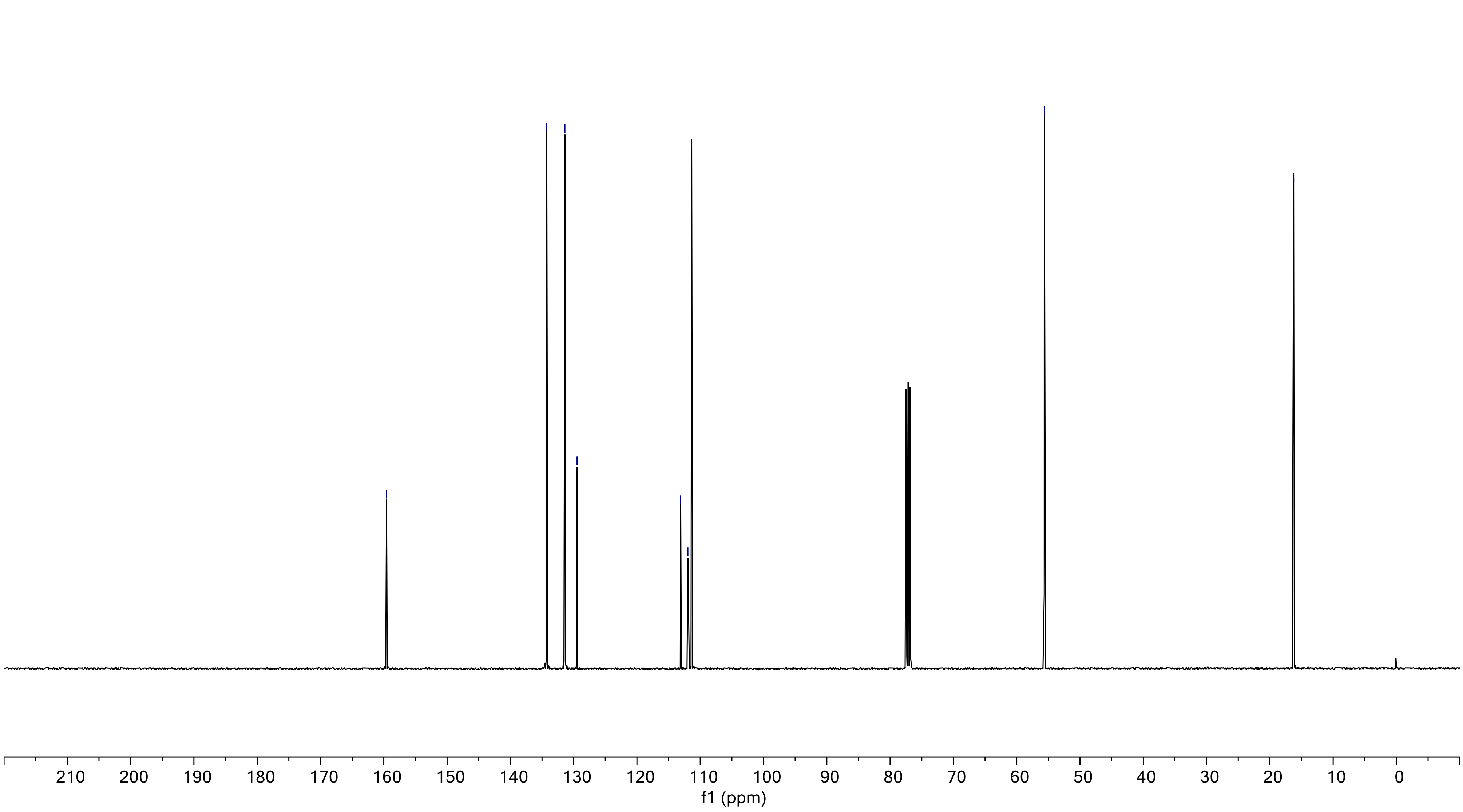

^{1}H NMR (400 MHz, $CDCl_3$)

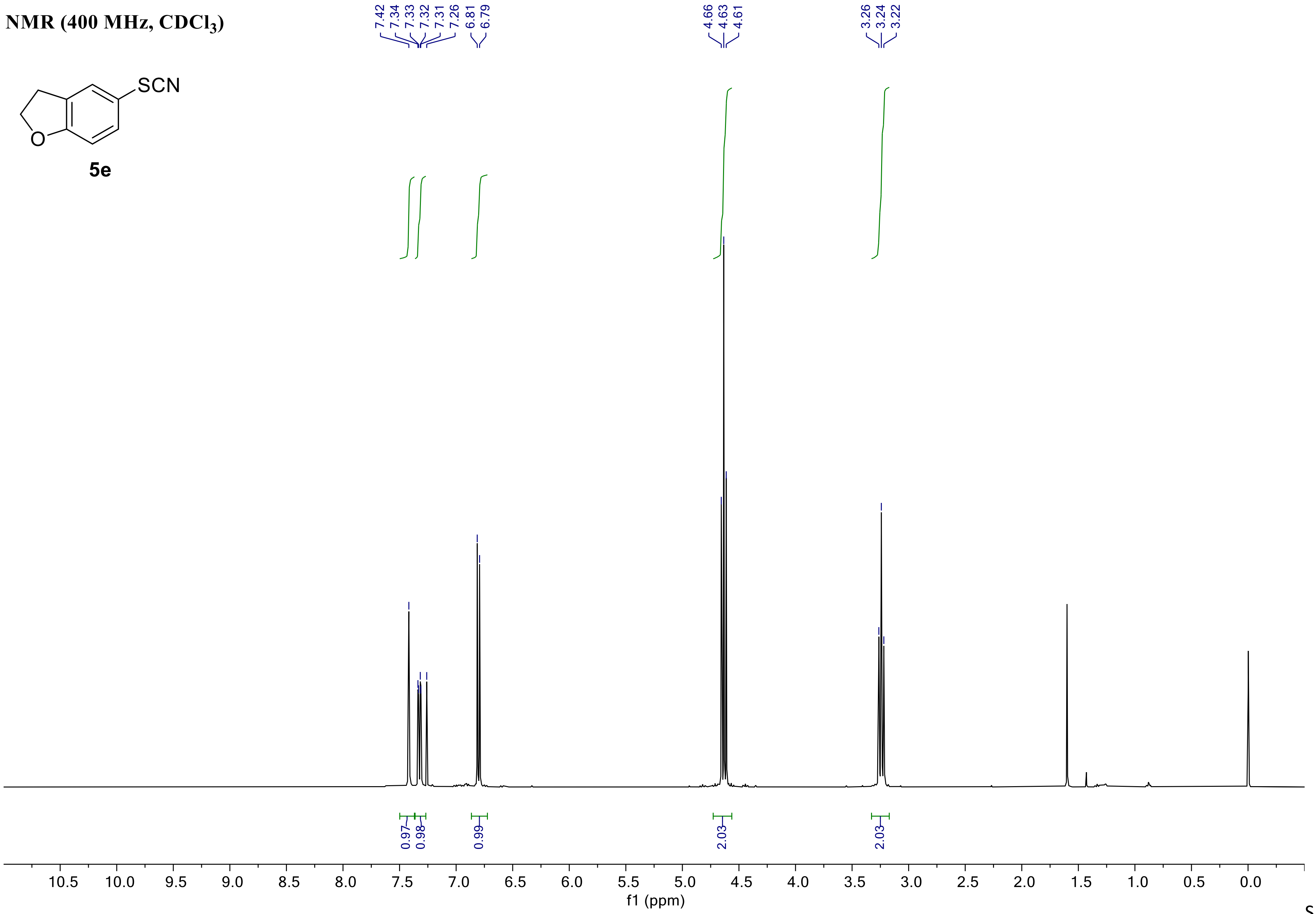

$^{13}C\{^1H\}$ NMR (101 MHz, $CDCl_3$)

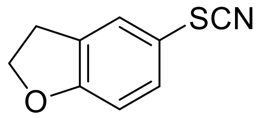

5e

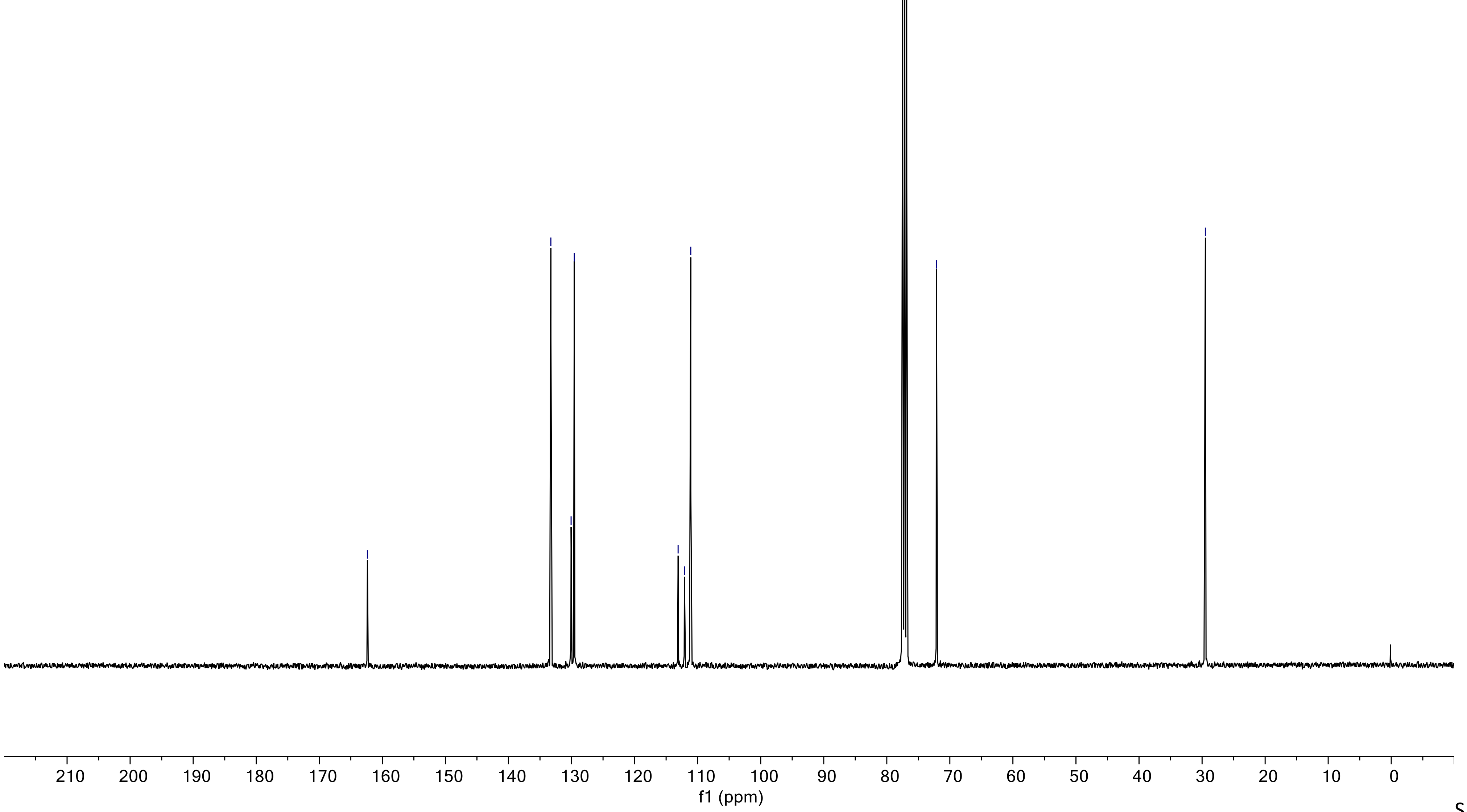

^{1}H NMR (400 MHz, $CDCl_3$)

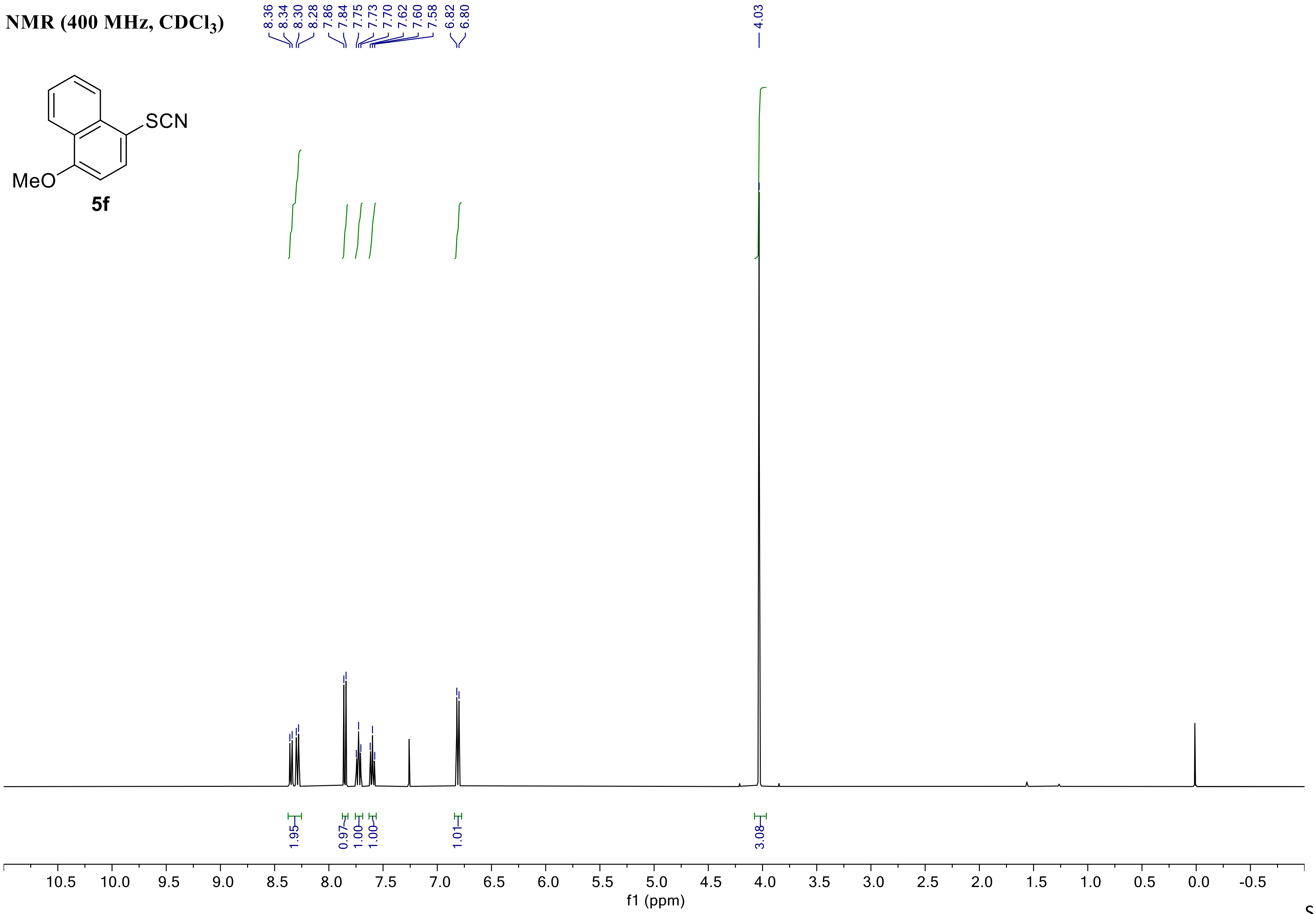

$^{13}C\{^1H\}$ NMR (101 MHz, $CDCl_3$)

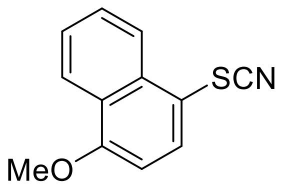

5f

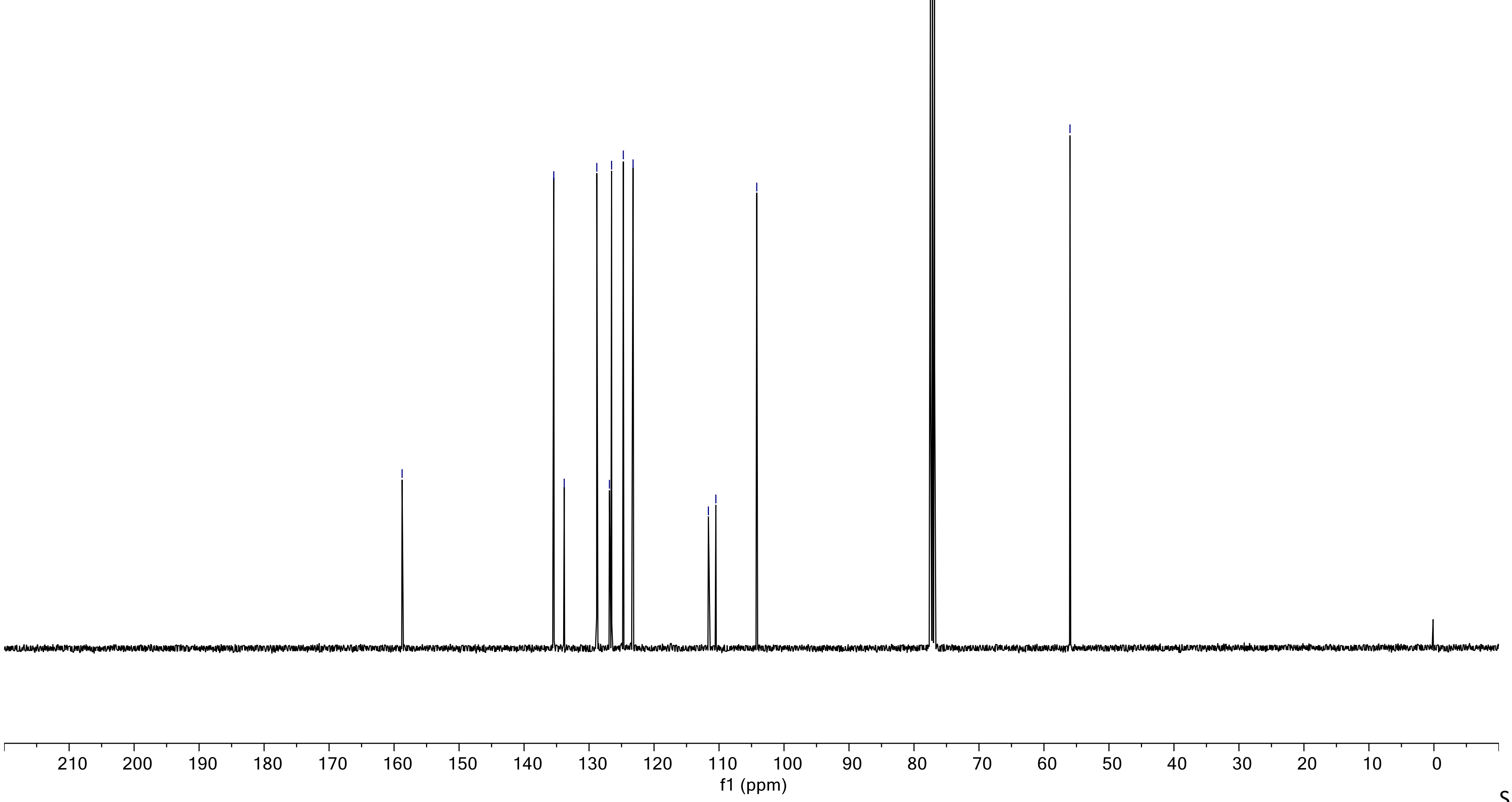

^{1}H NMR (400 MHz, $CDCl_3$)

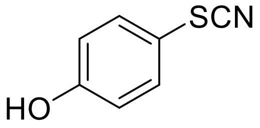

5g

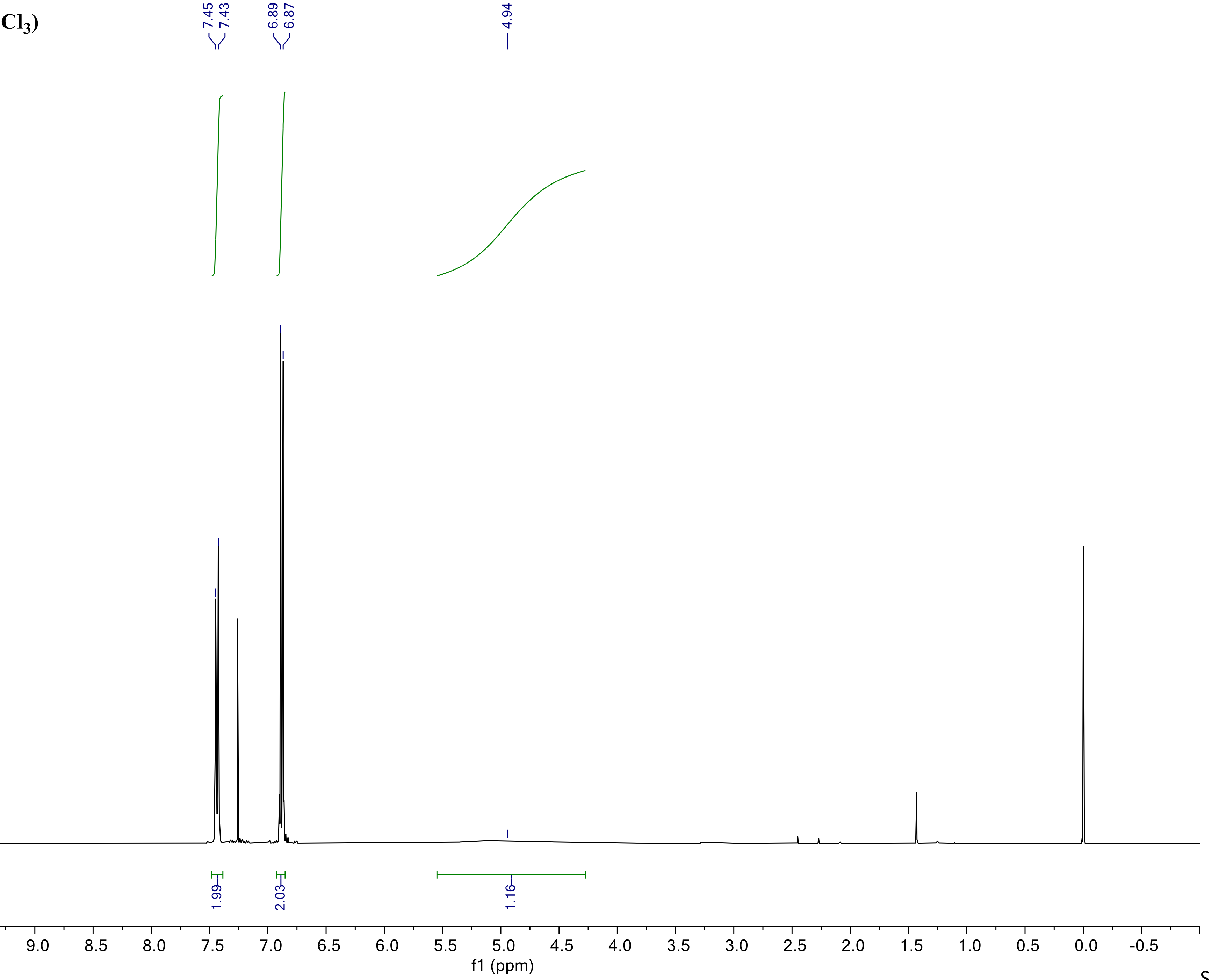

$^{13}C\{^{1}H\}$ NMR (101 MHz, $CDCl_3$)

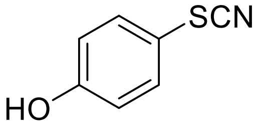

5g

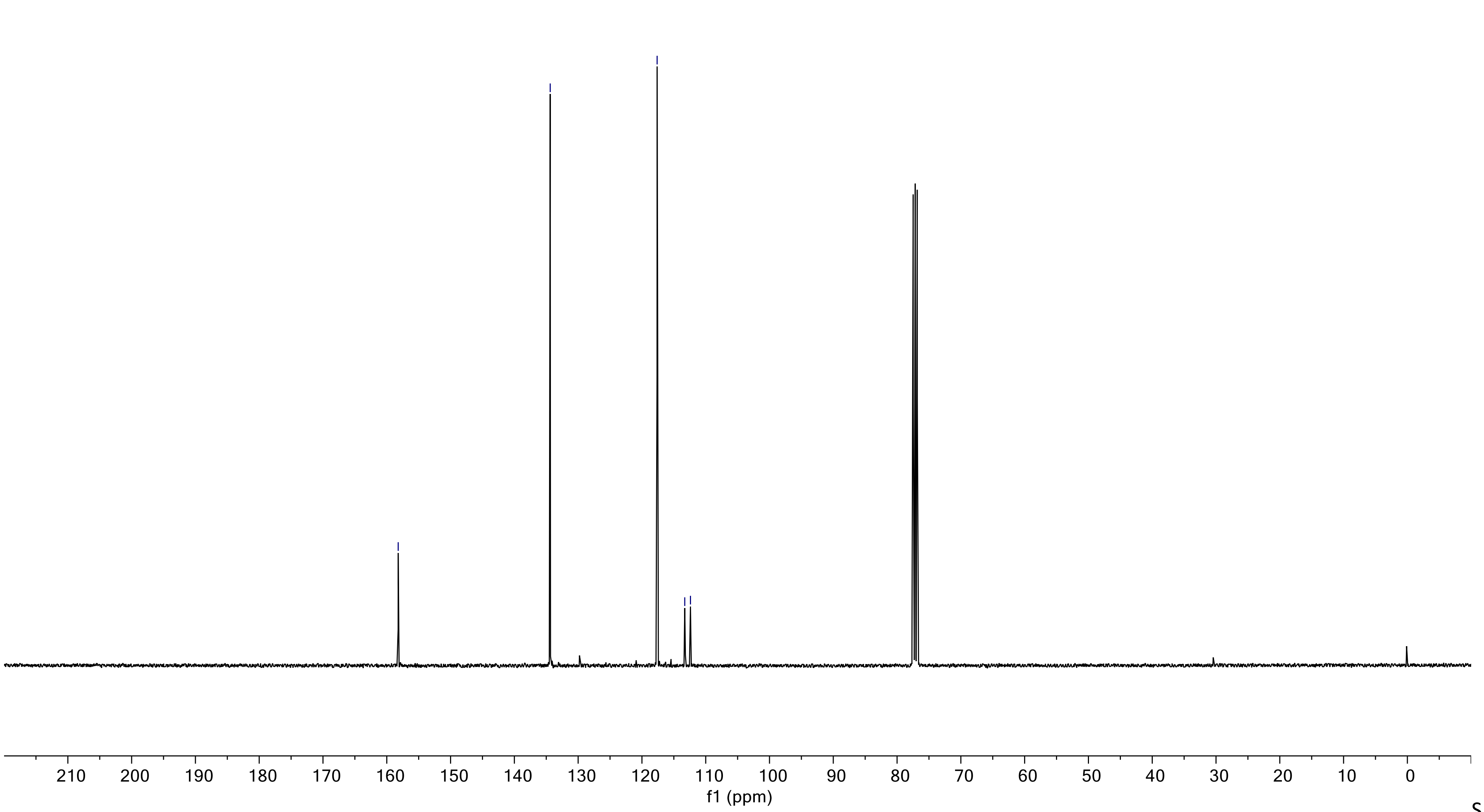

^{1}H NMR (400 MHz, $CDCl_3$)

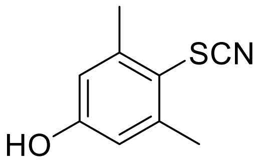

5h

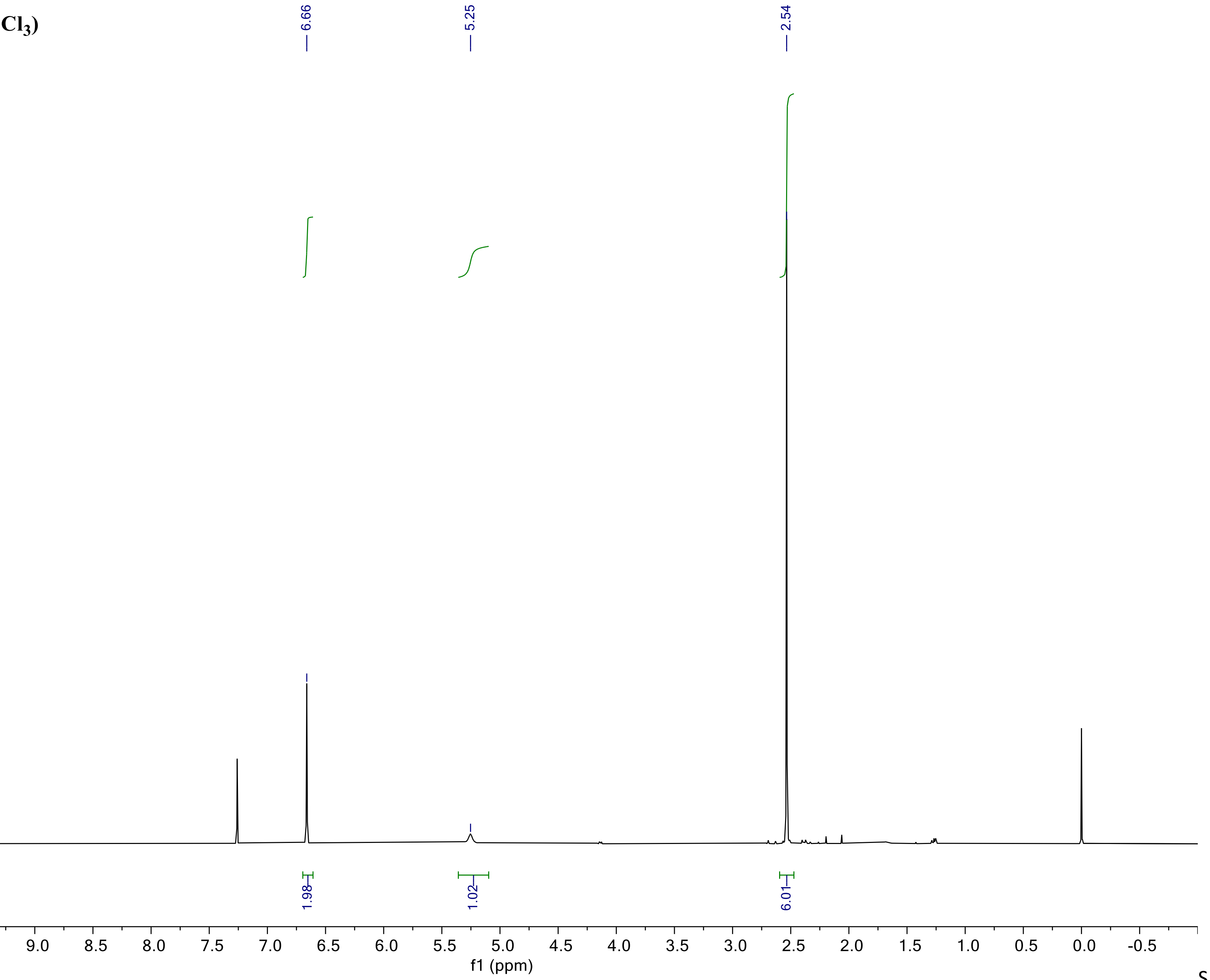

$^{13}C\{^1H\}$ NMR (101 MHz, $CDCl_3$)

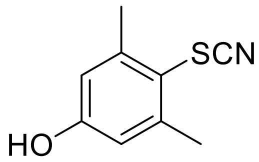

5h

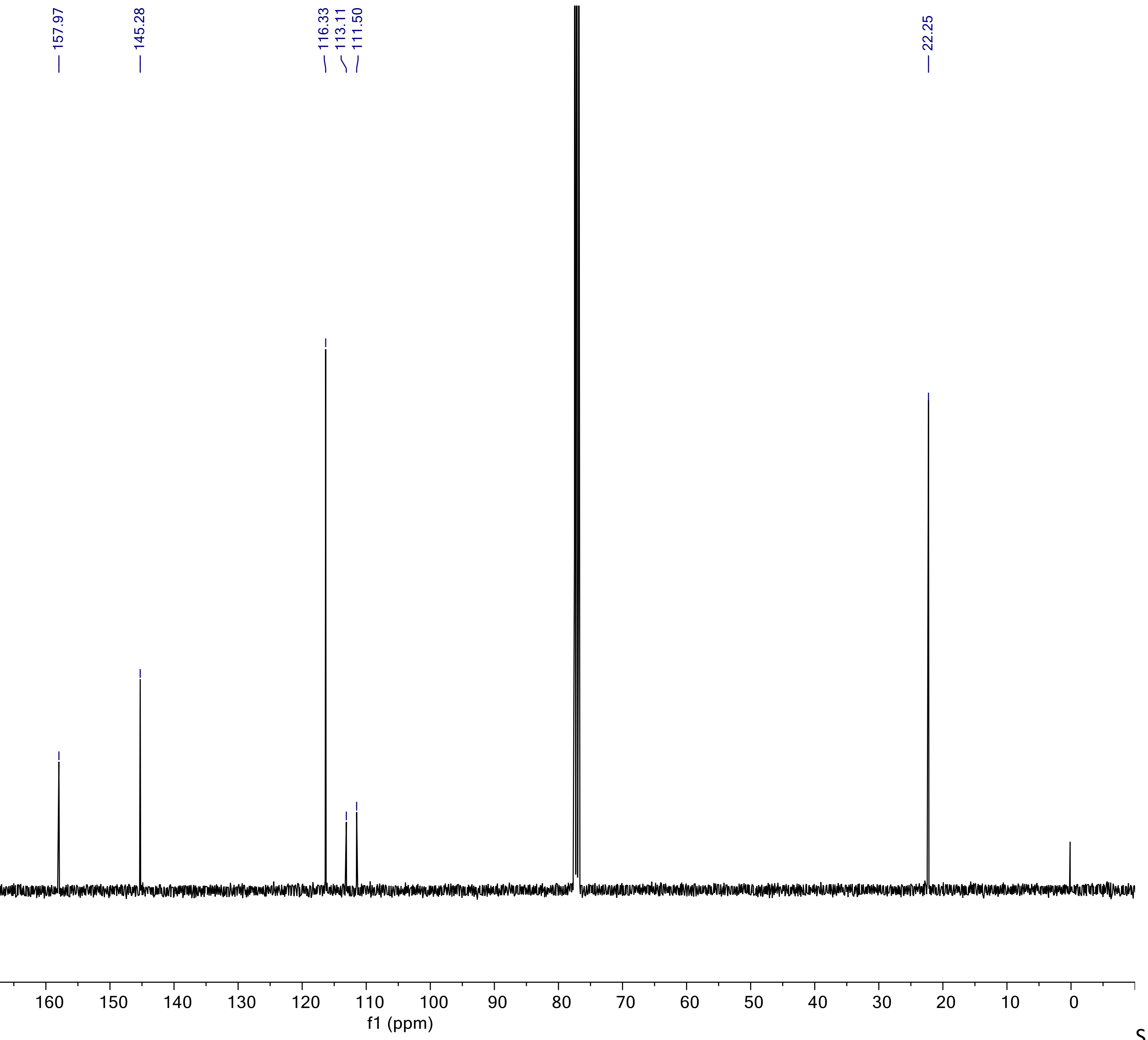

^{1}H NMR (400 MHz, $CDCl_3$)

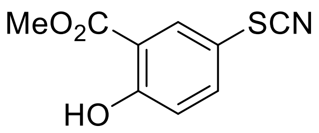

5i

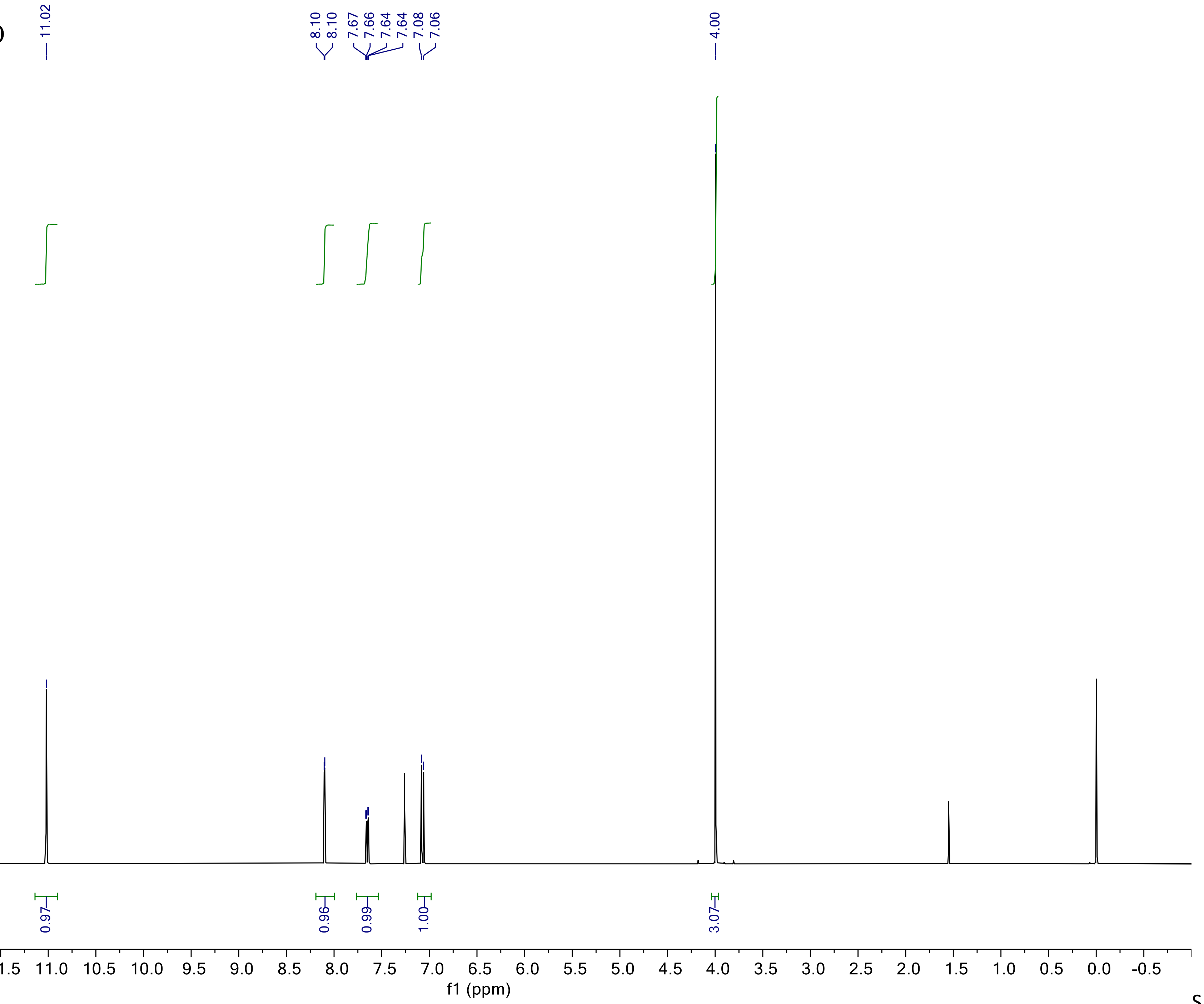

$^{13}C\{^1H\}$ NMR (101 MHz, $CDCl_3$)

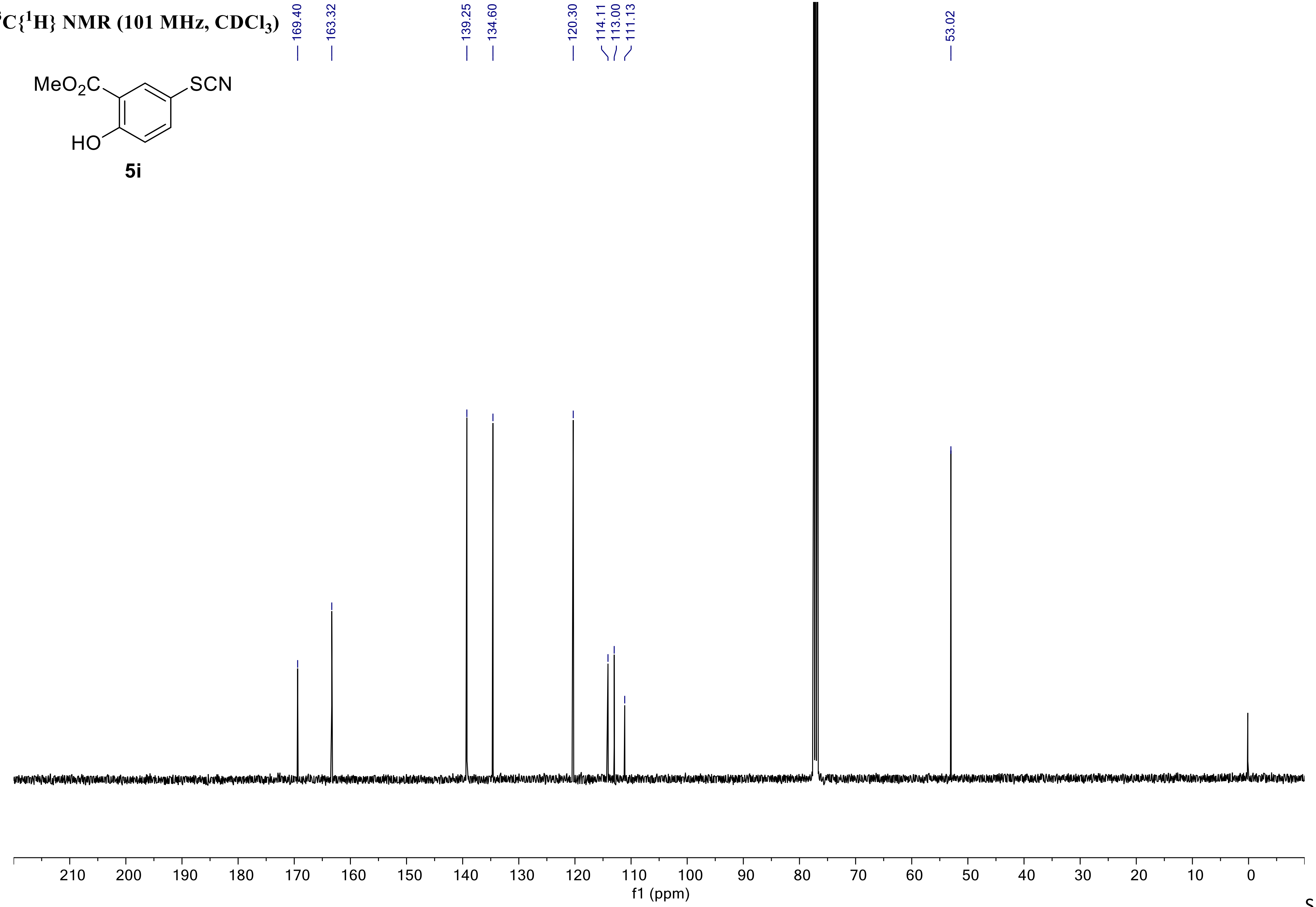

^{1}H NMR (400 MHz, CDCl$_3$)

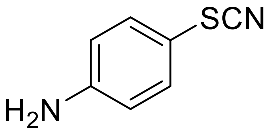

5j

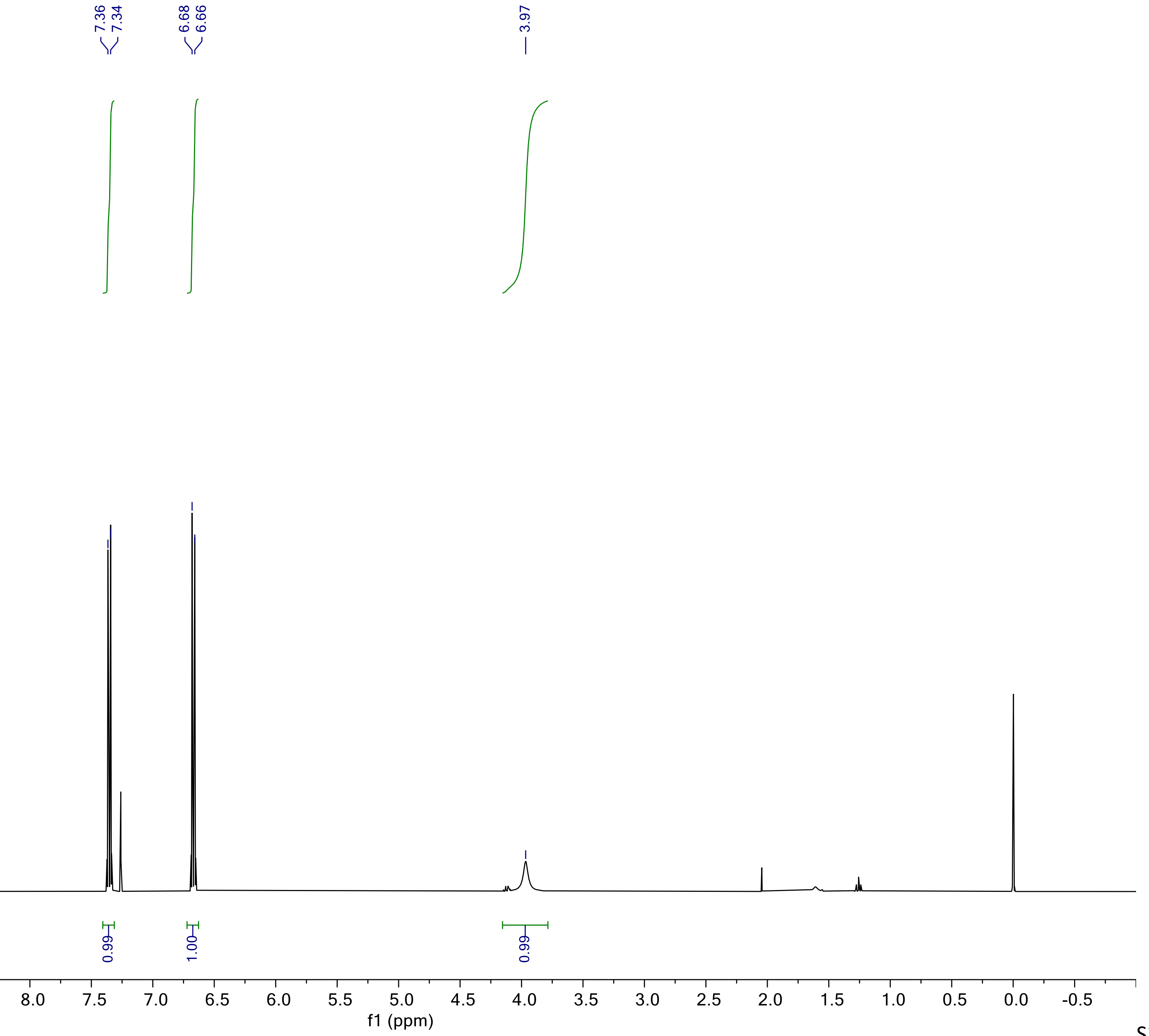

$^{13}C\{^1H\}$ NMR (101 MHz, $CDCl_3$)

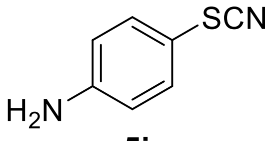

5j

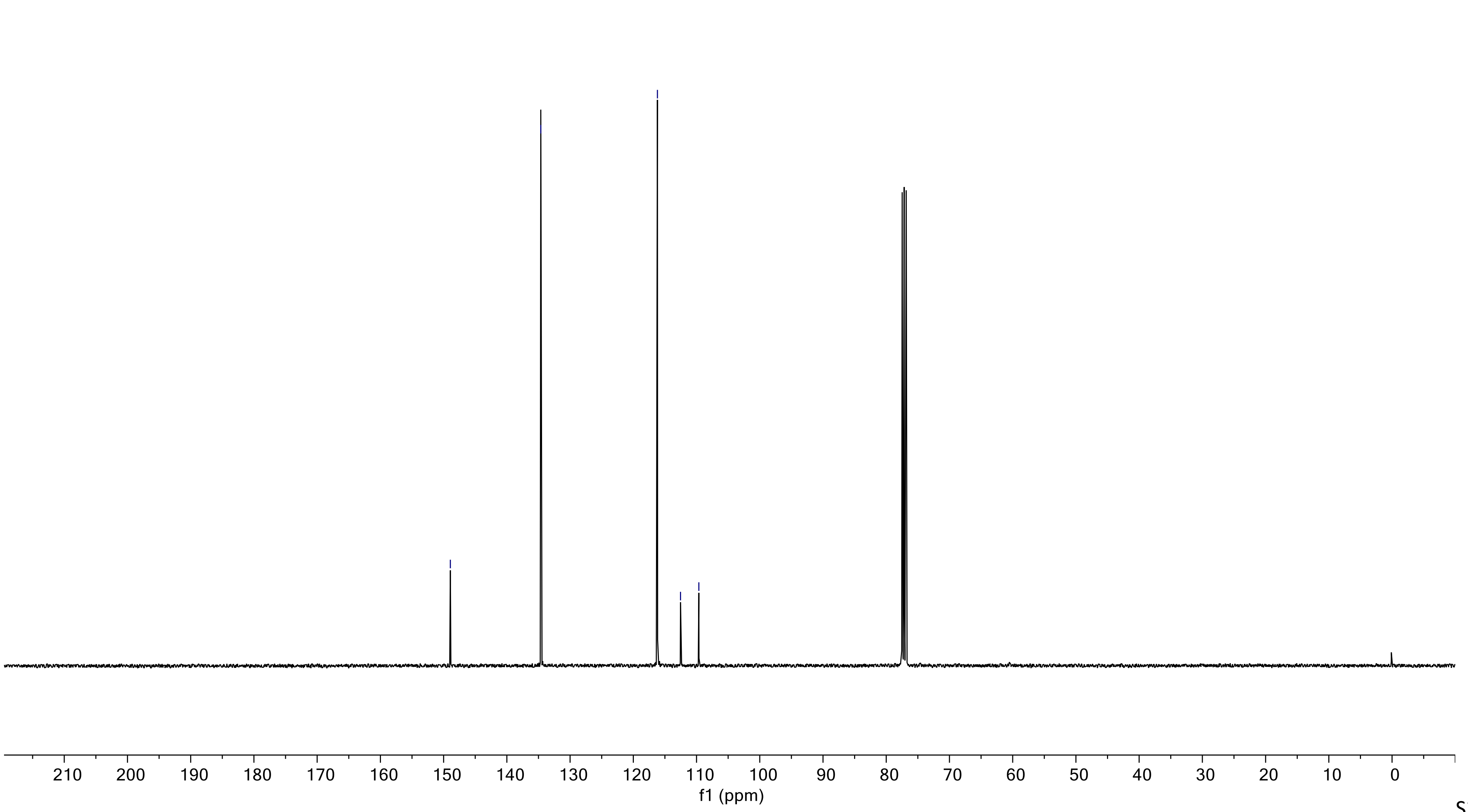

^{1}H NMR (400 MHz, $CDCl_3$)

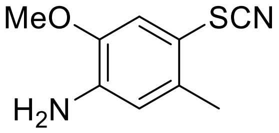

5k

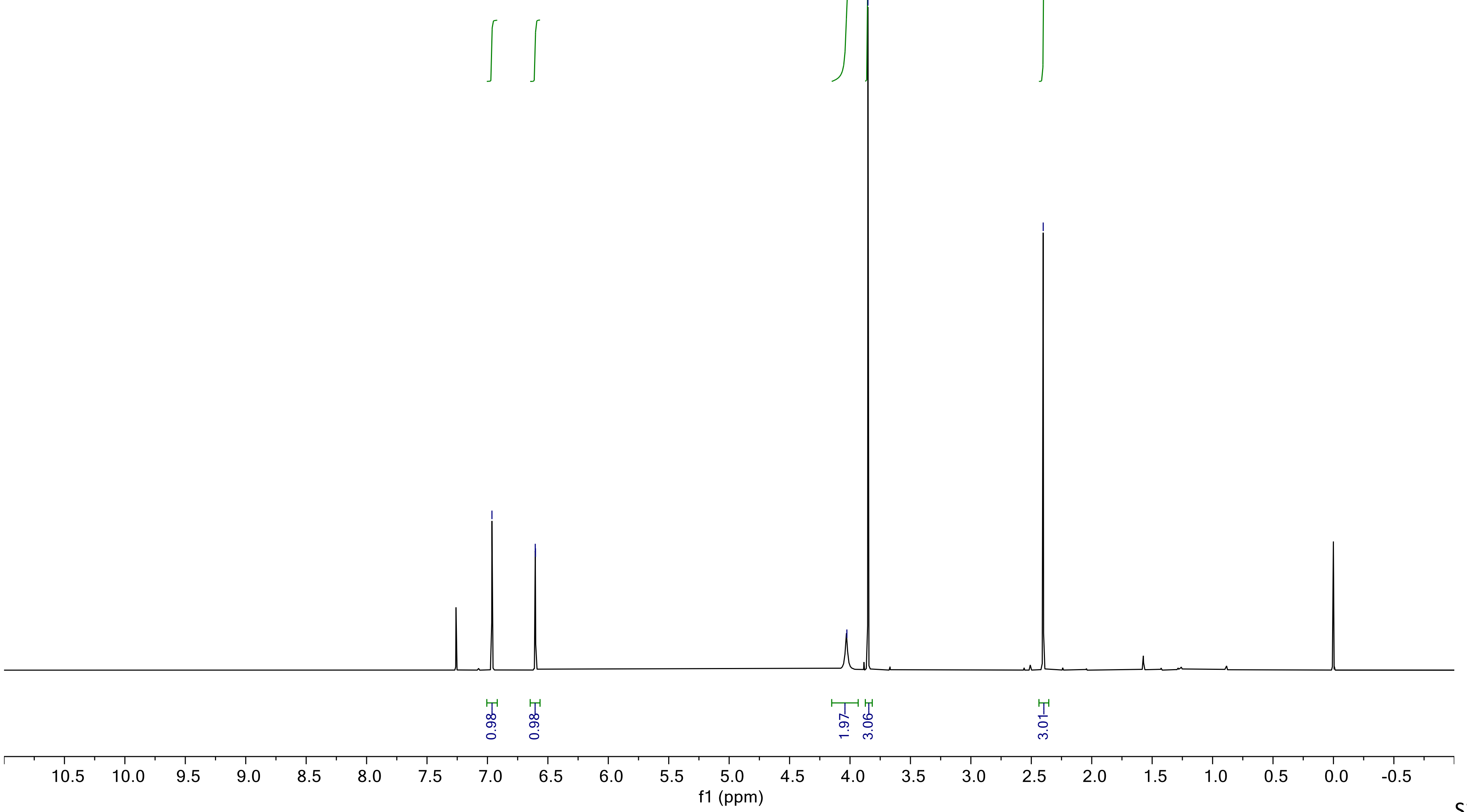

$^{13}C\{^1H\}$ NMR (101 MHz, $CDCl_3$)

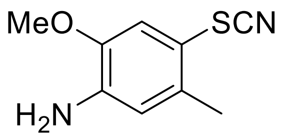

5k

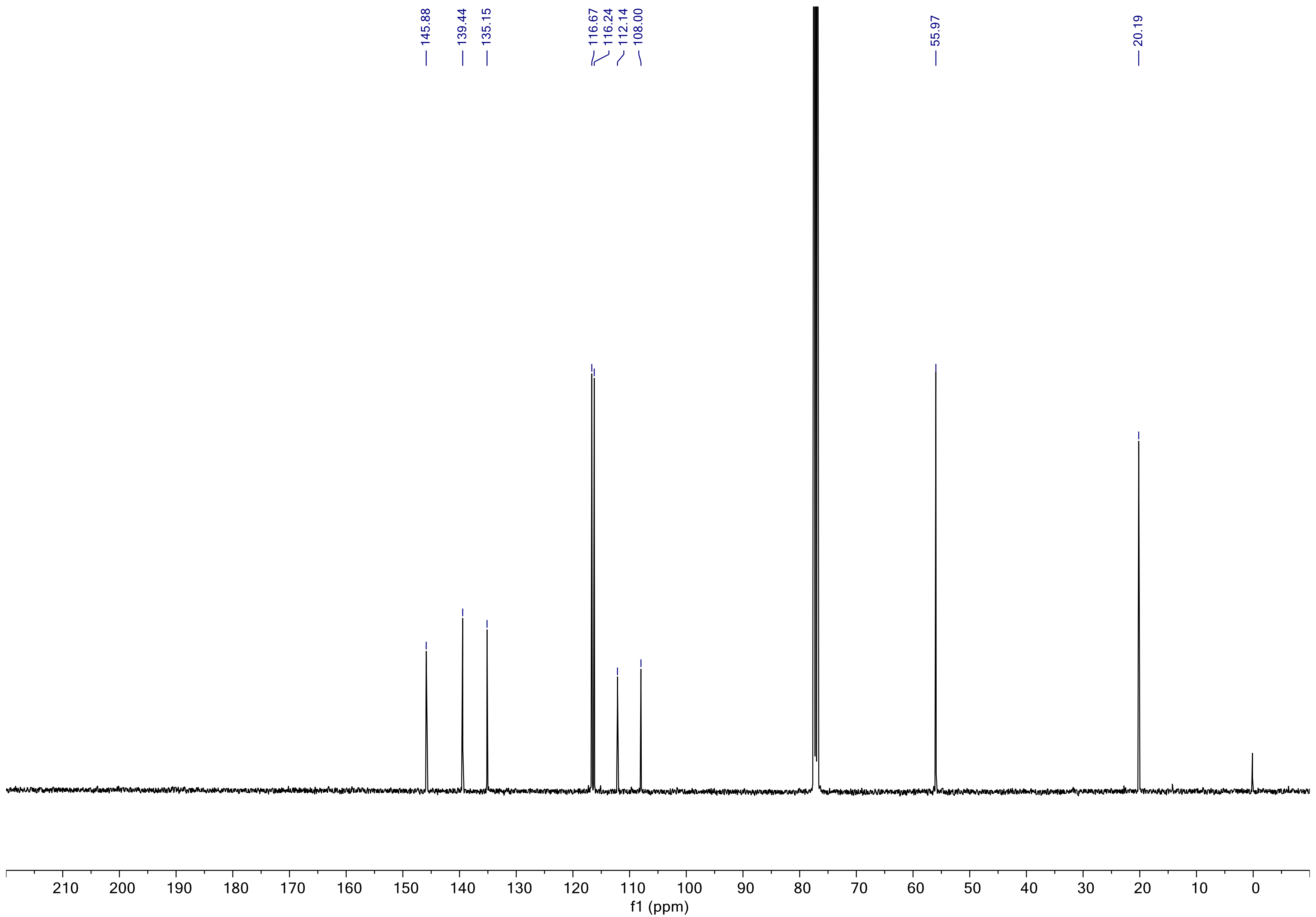

^{1}H NMR (400 MHz, $CDCl_3$)

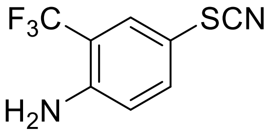

5l

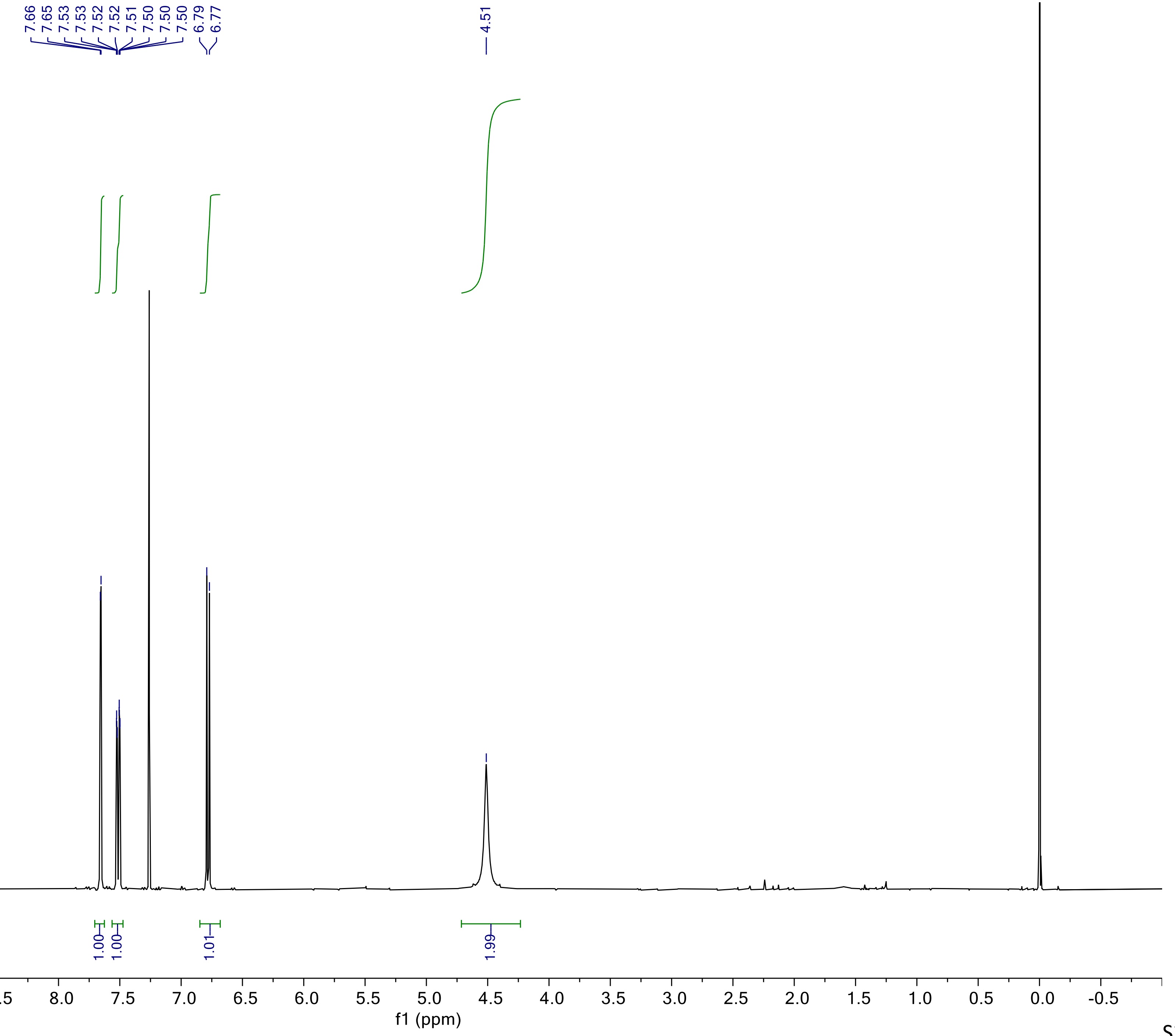

$^{13}C\{^1H\}$ NMR (101 MHz, $CDCl_3$)

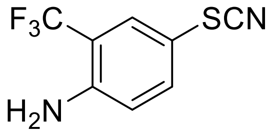

5l

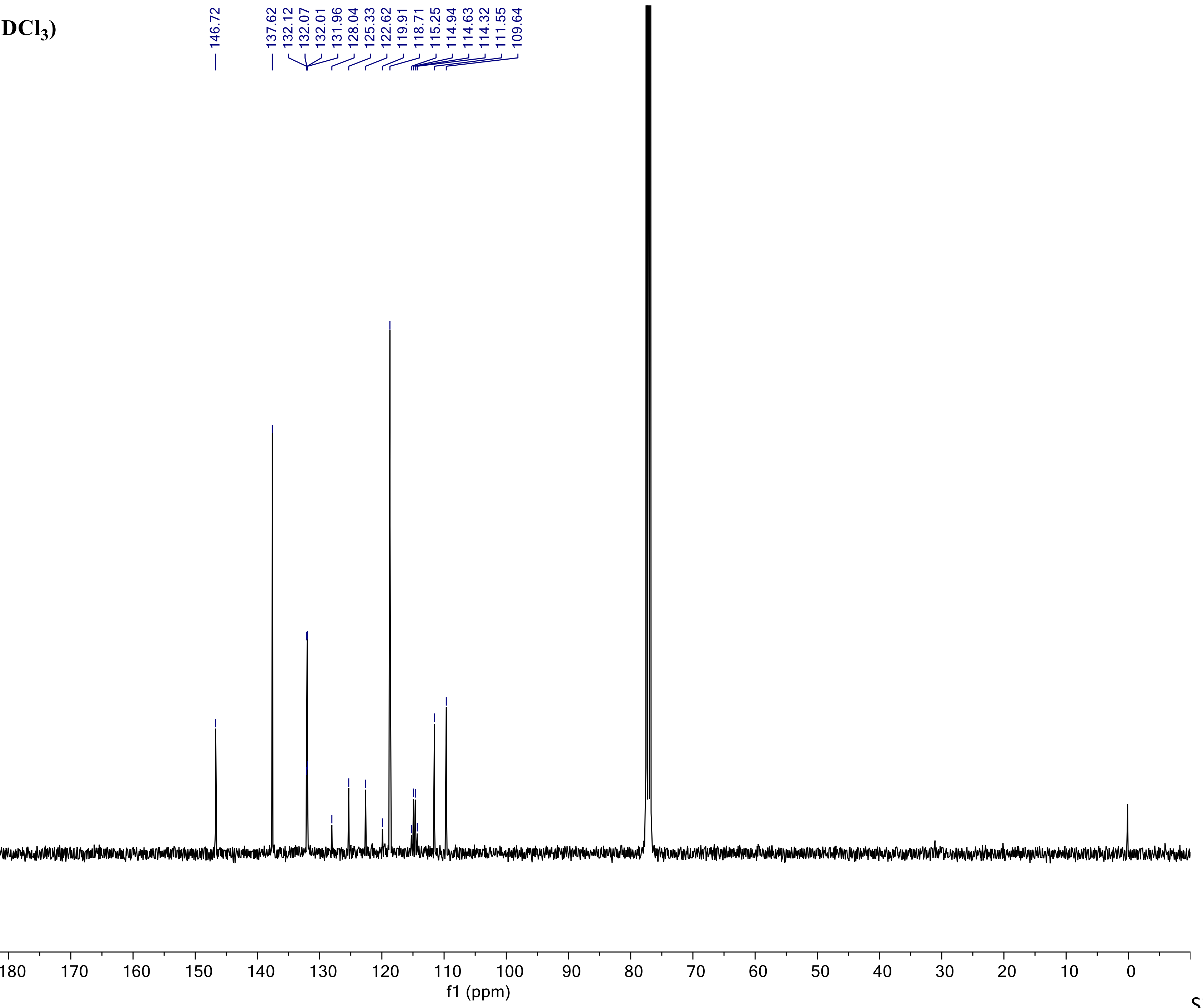

^{1}H NMR (400 MHz, $CDCl_3$)

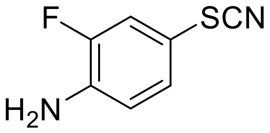

5m

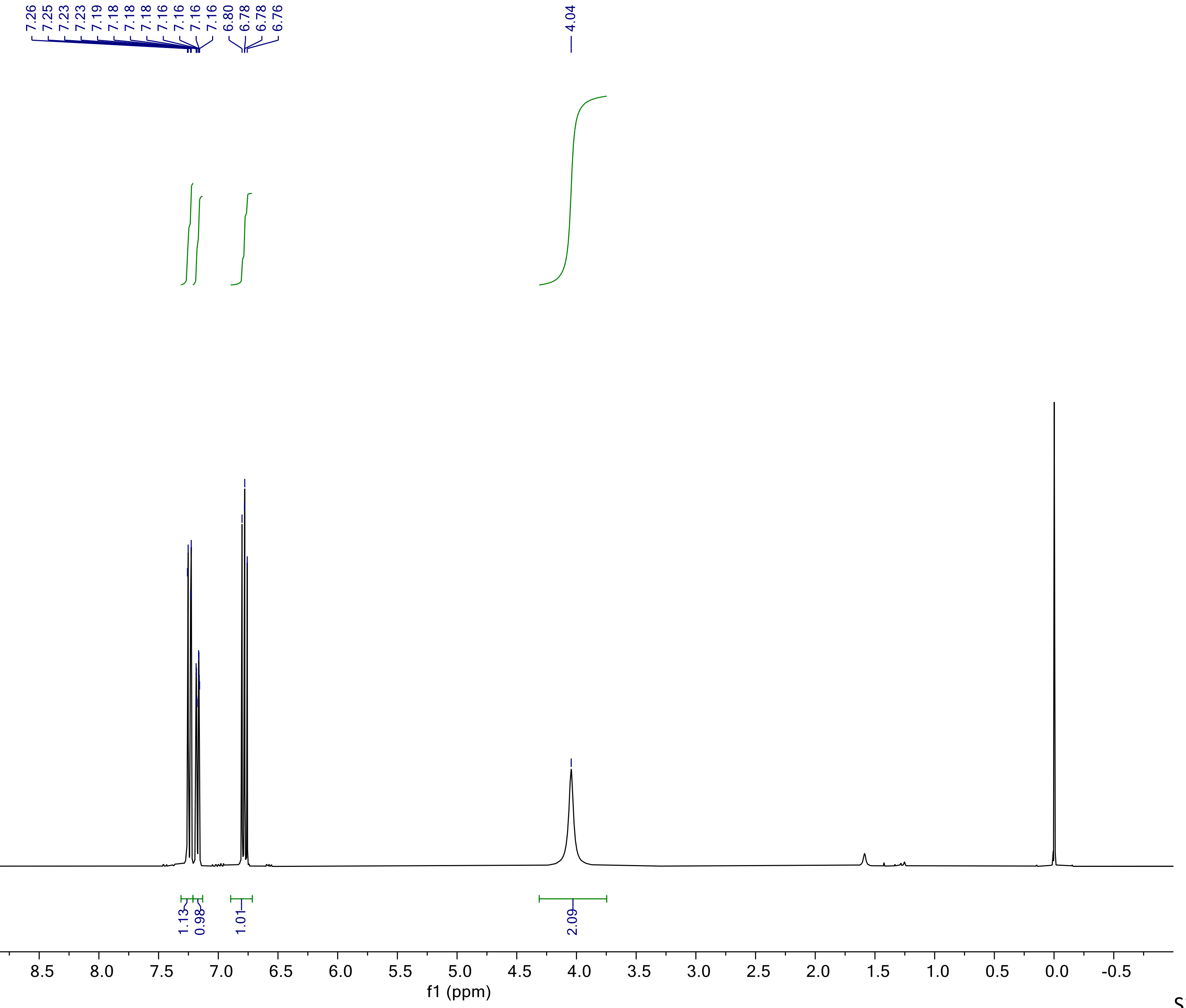

$^{13}C\{^1H\}$ NMR (101 MHz, $CDCl_3$)

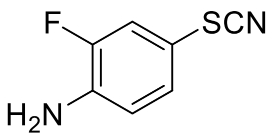

5m

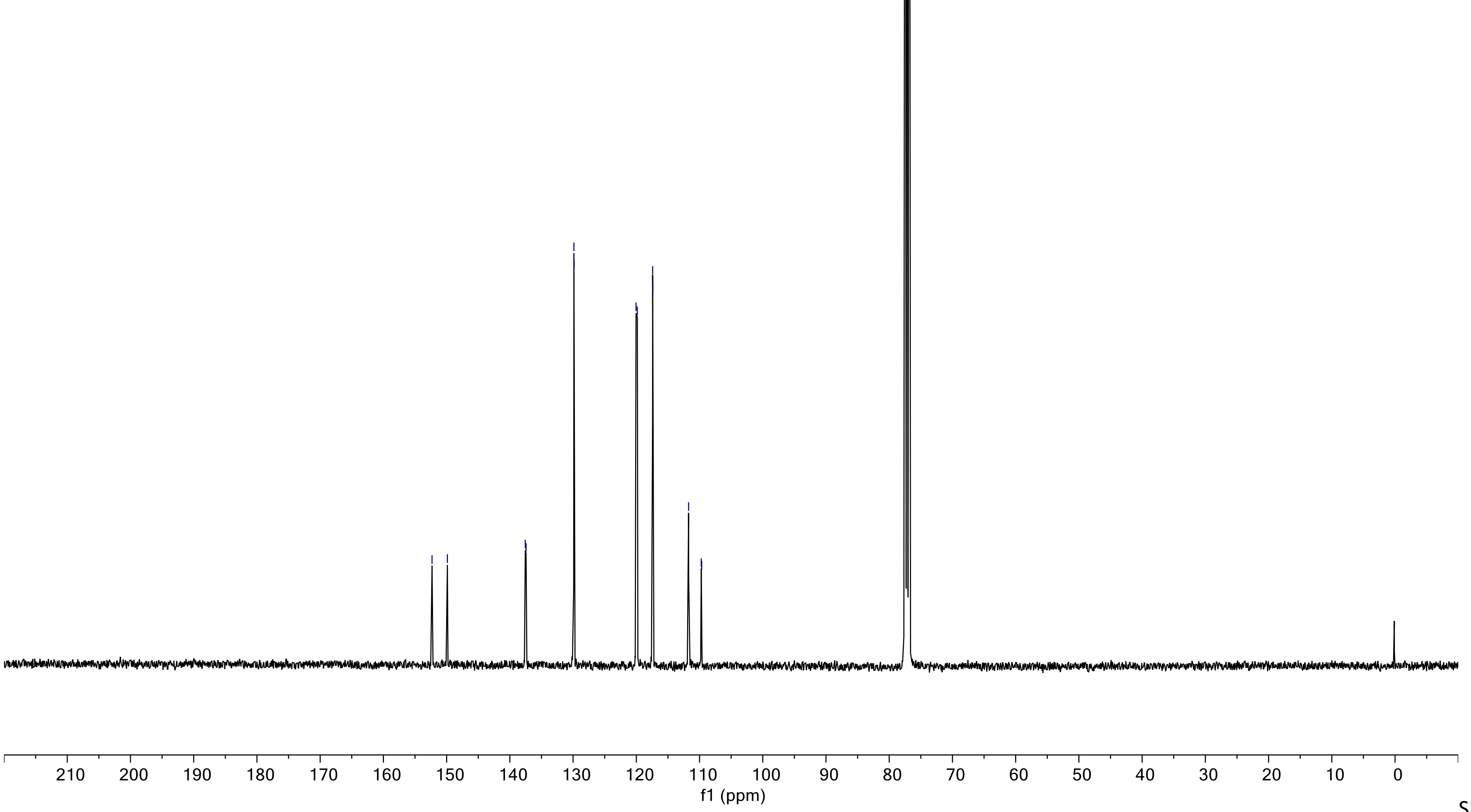

^{1}H NMR (400 MHz, $CDCl_3$)

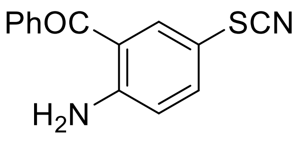

5n

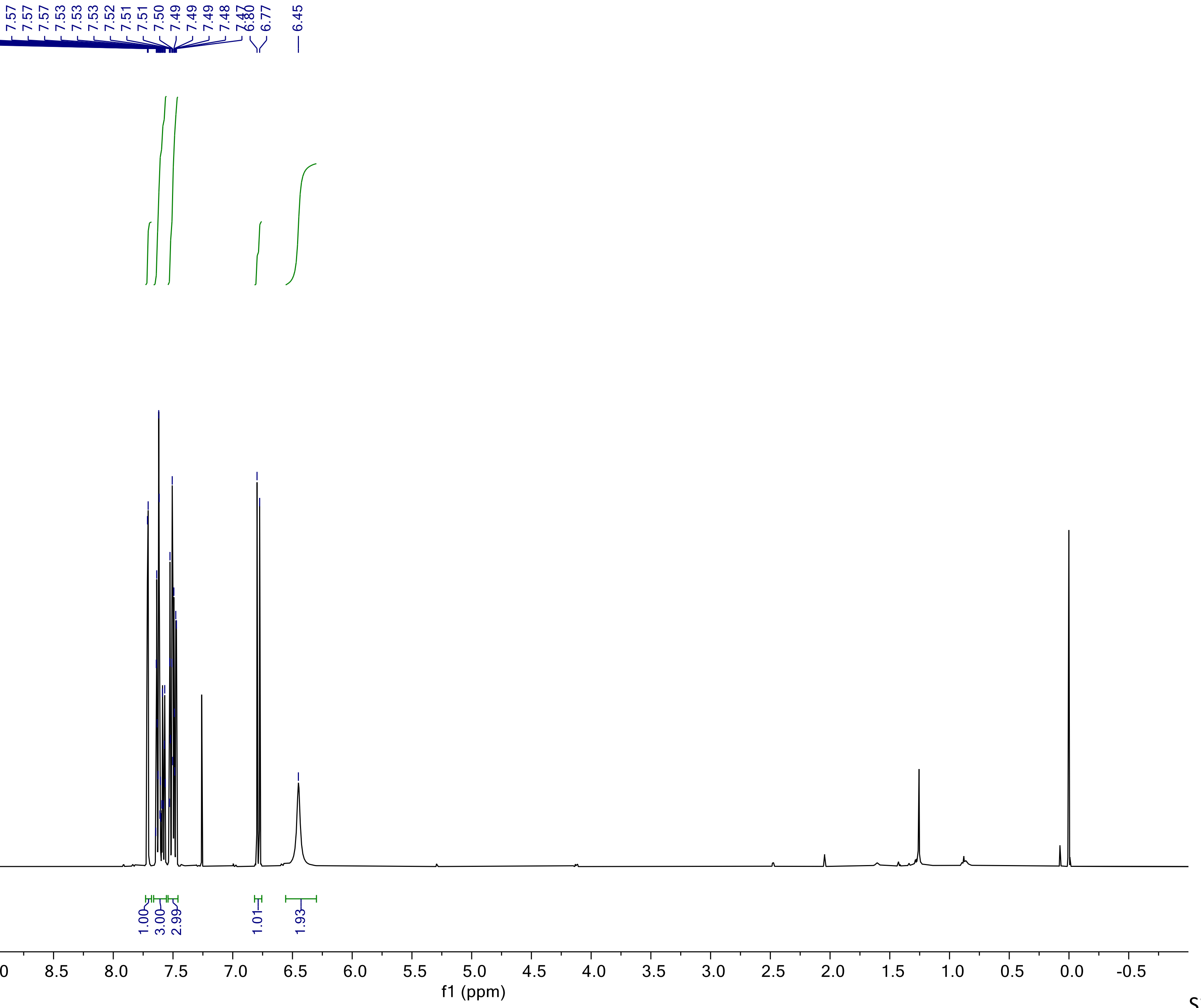

$^{13}C\{^{1}H\}$ NMR (101 MHz, $CDCl_3$)

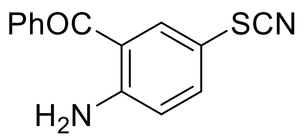

5n

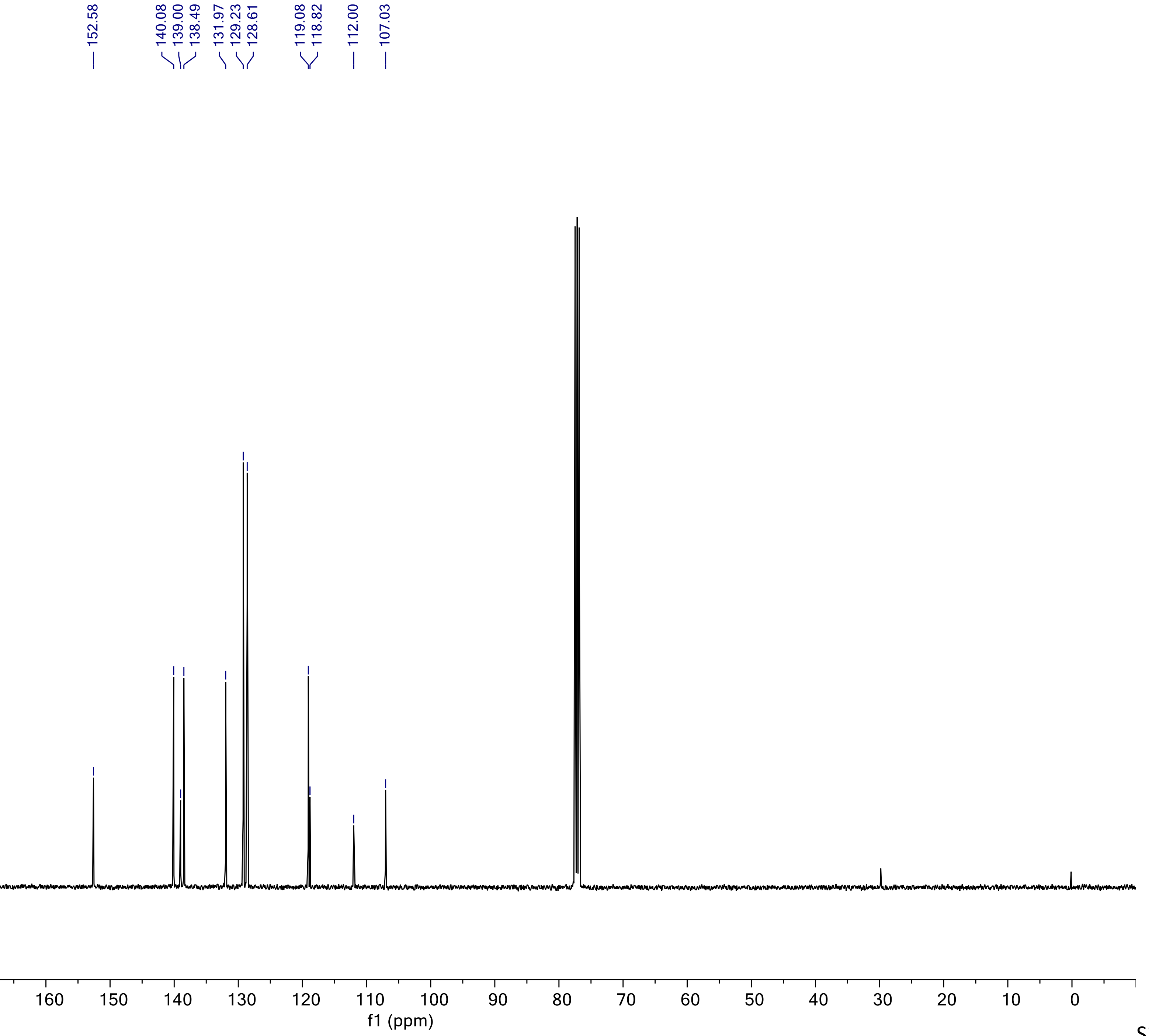

^{1}H NMR (400 MHz, $CDCl_3$)

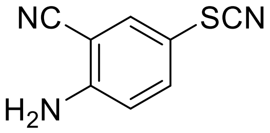

5o

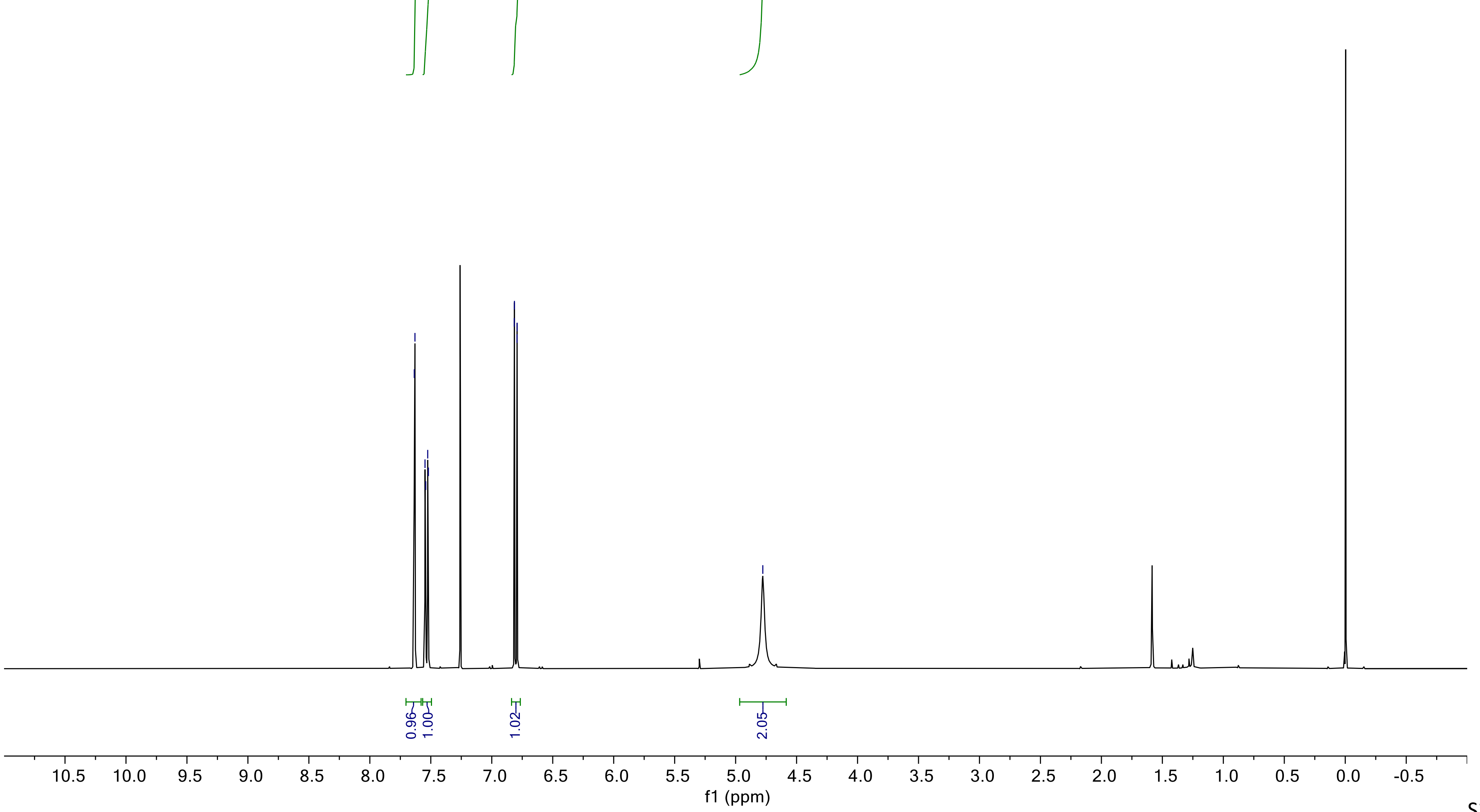

$^{13}C\{^{1}H\}$ NMR (101 MHz, $CDCl_3$)

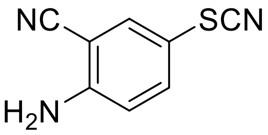

5o

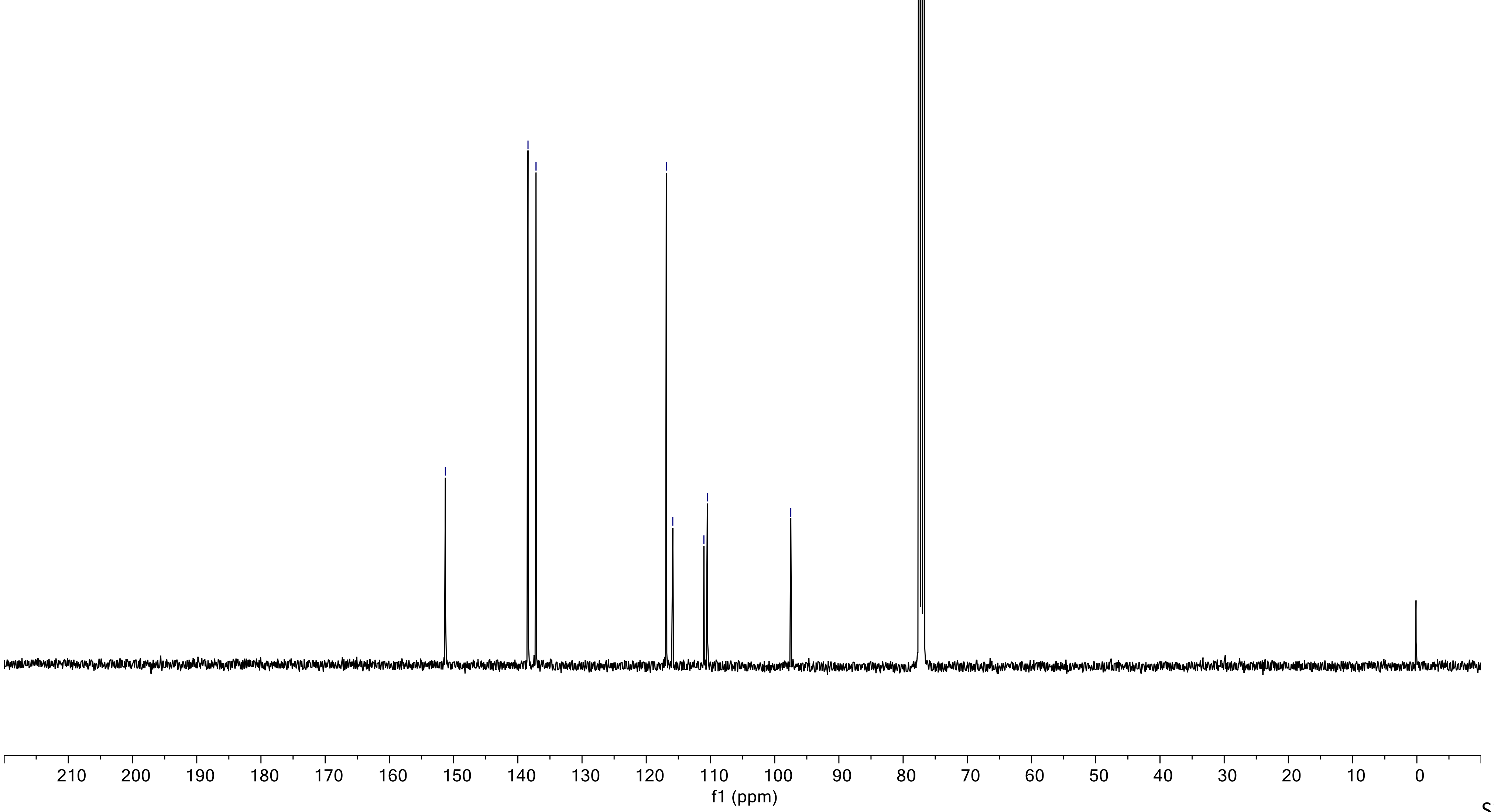

^{1}H NMR (400 MHz, $CDCl_3$)

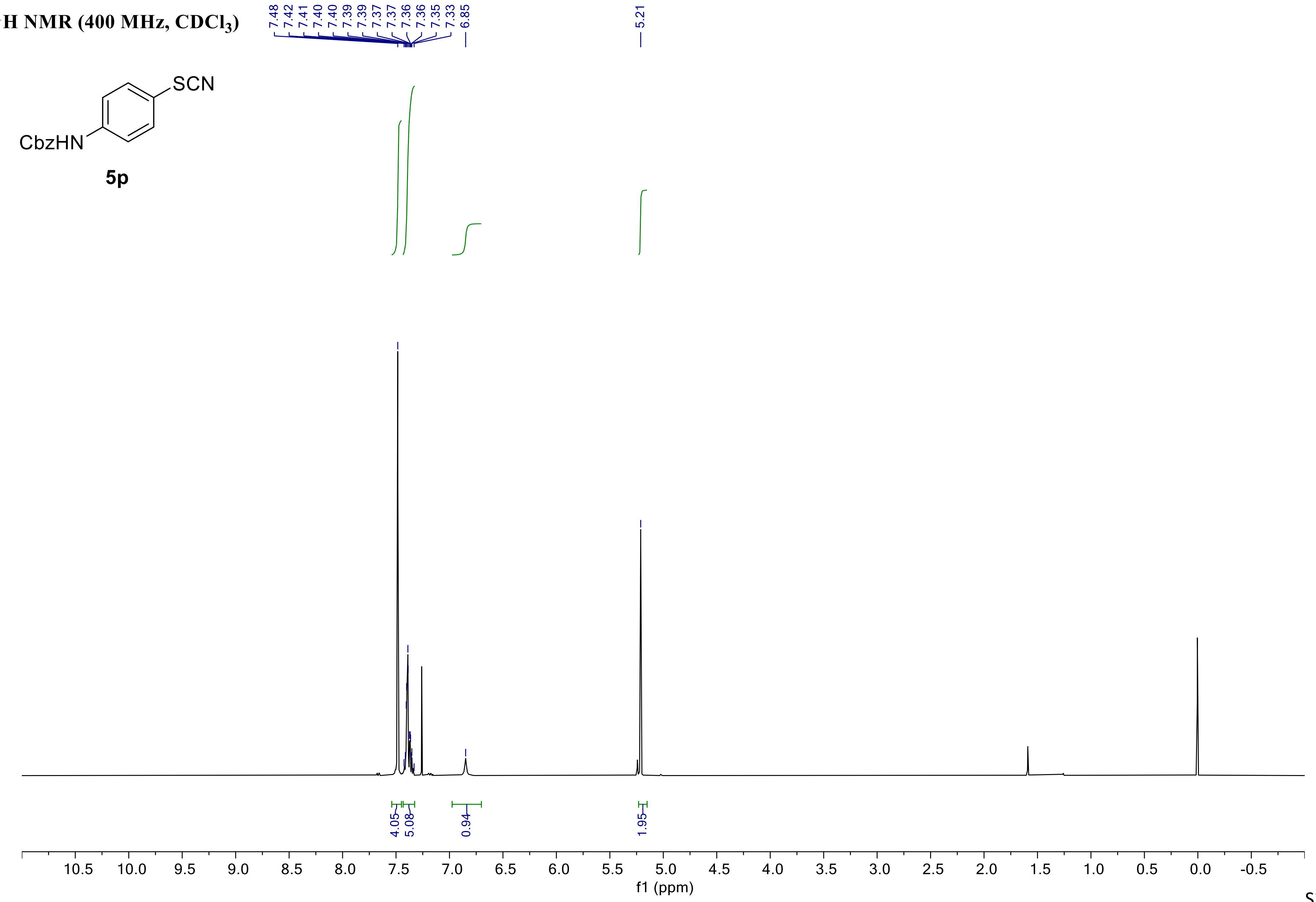

$^{13}C\{^{1}H\}$ NMR (101 MHz, $CDCl_3$)

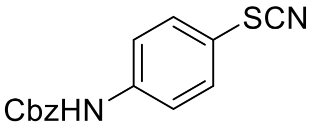

5p

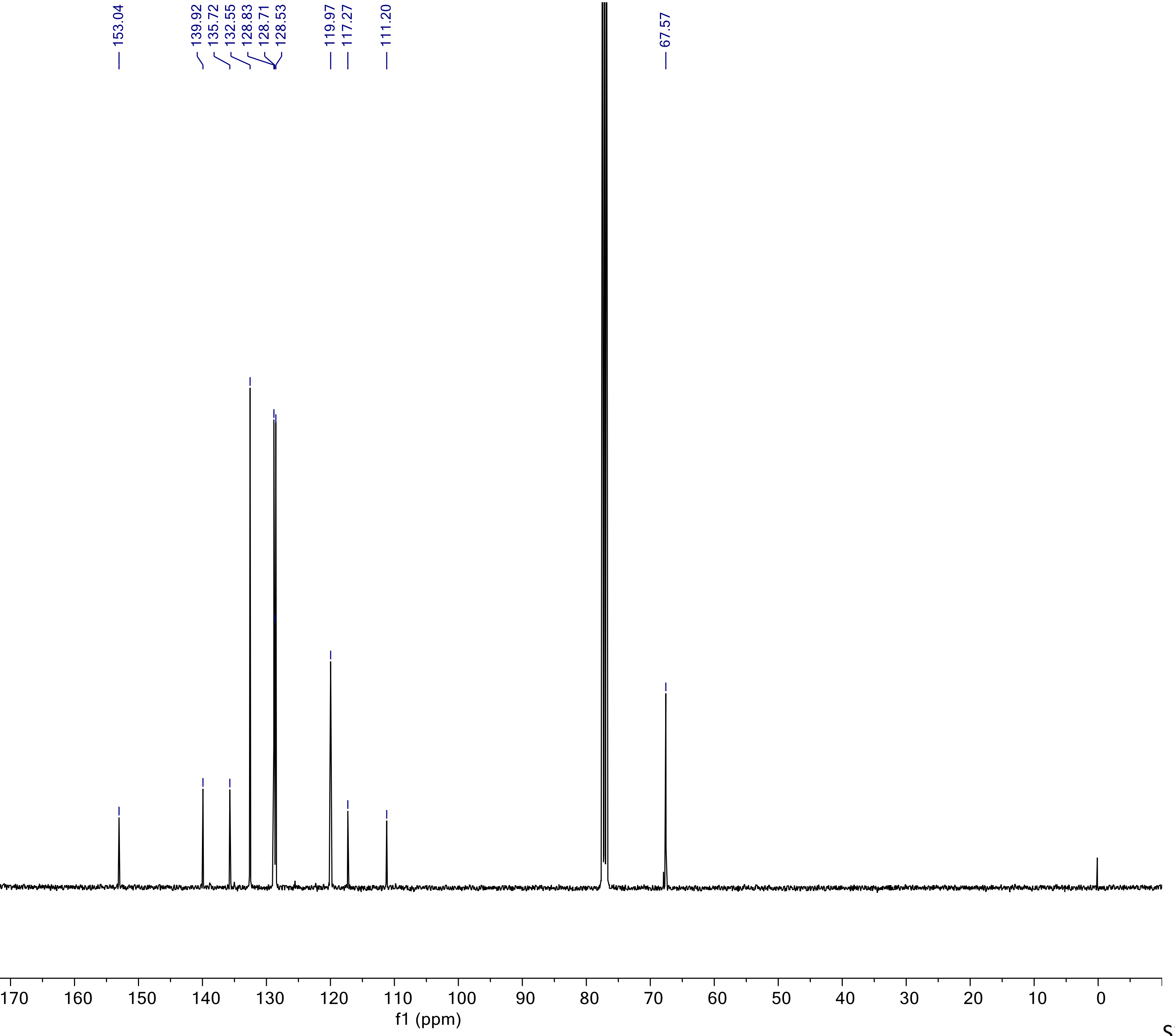

^{1}H NMR (400 MHz, $CDCl_3$)

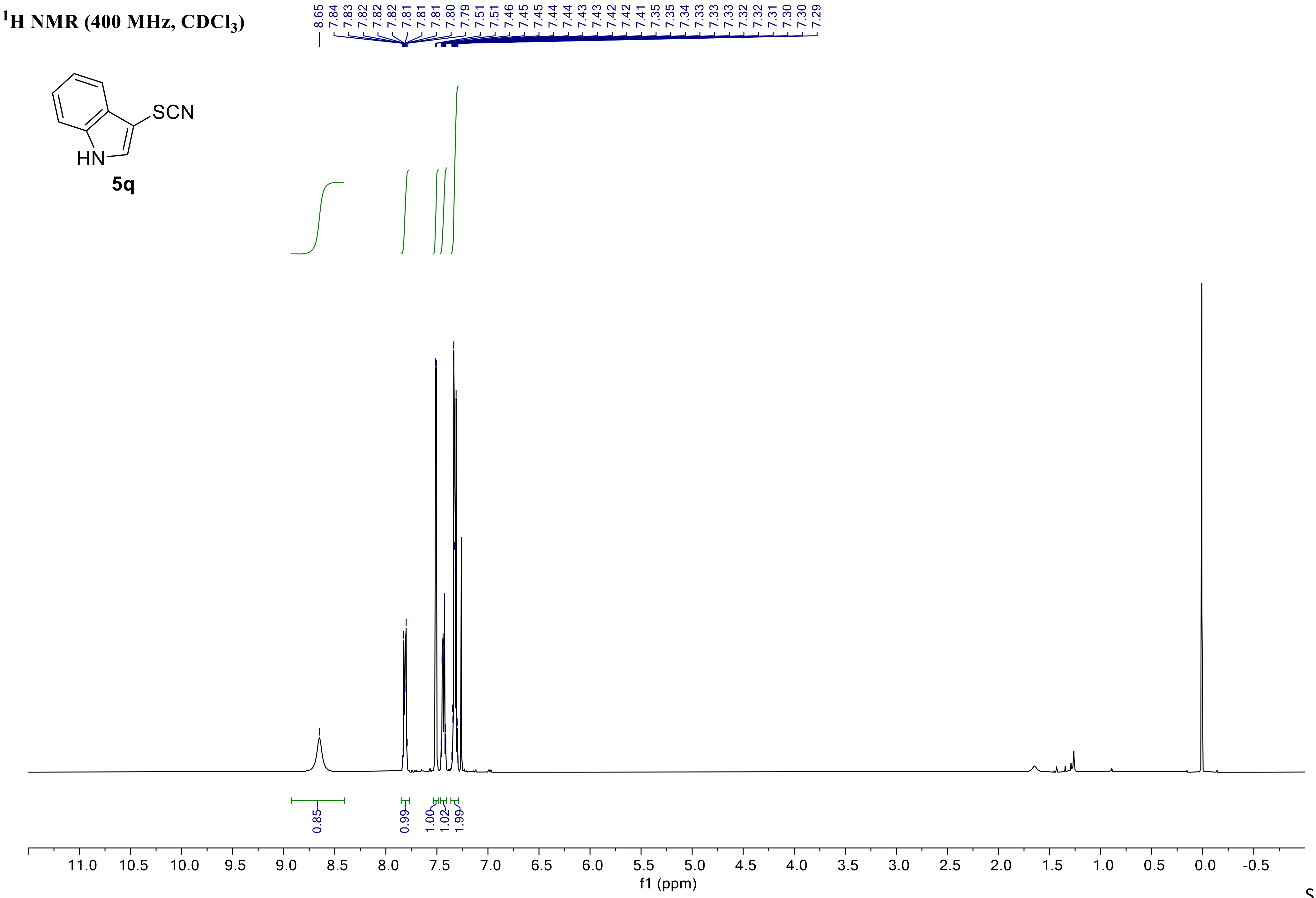

$^{13}C\{^{1}H\}$ NMR (101 MHz, $CDCl_3$)

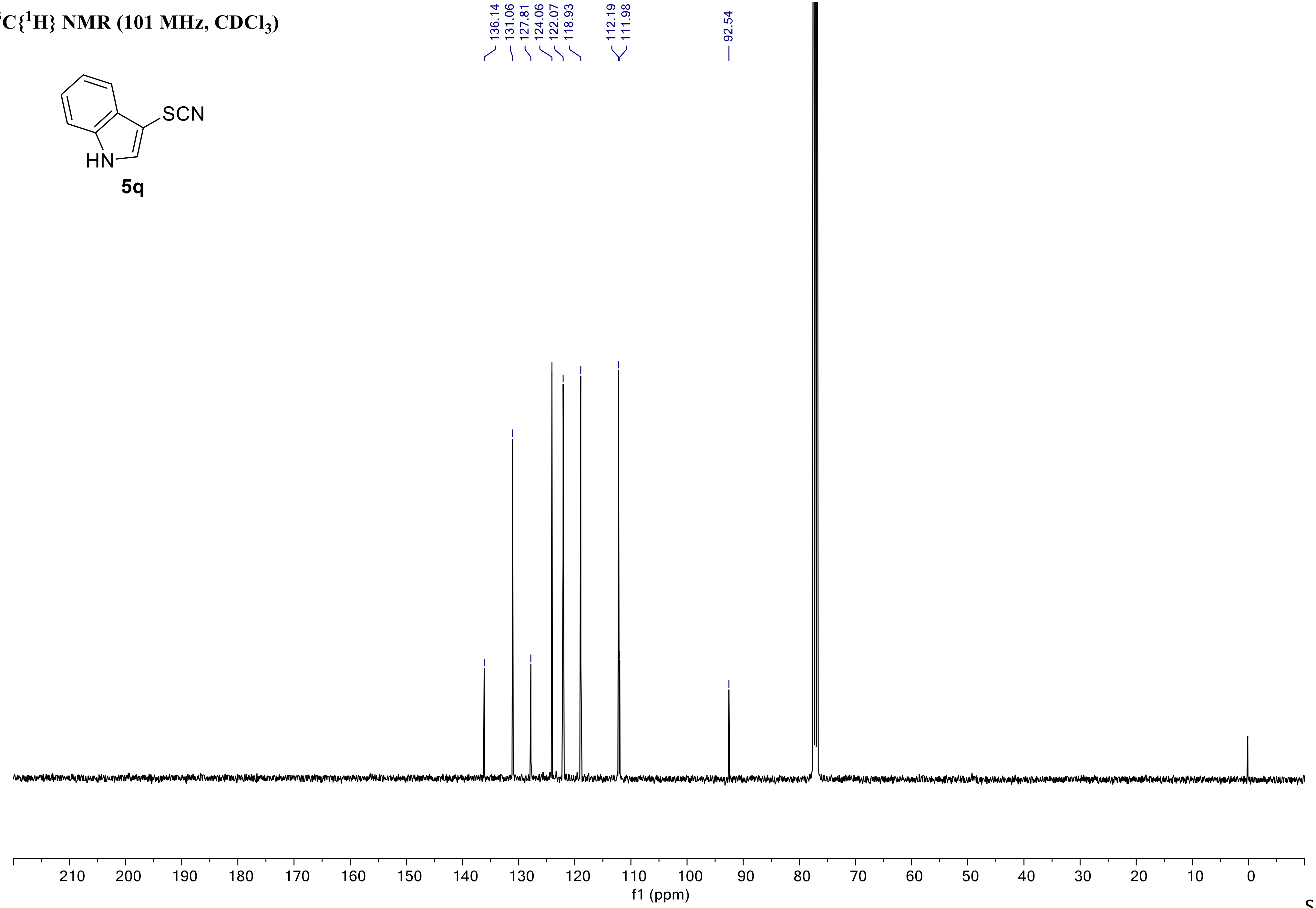

^{1}H NMR (400 MHz, DMSO-d_6)

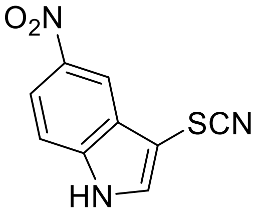

5r

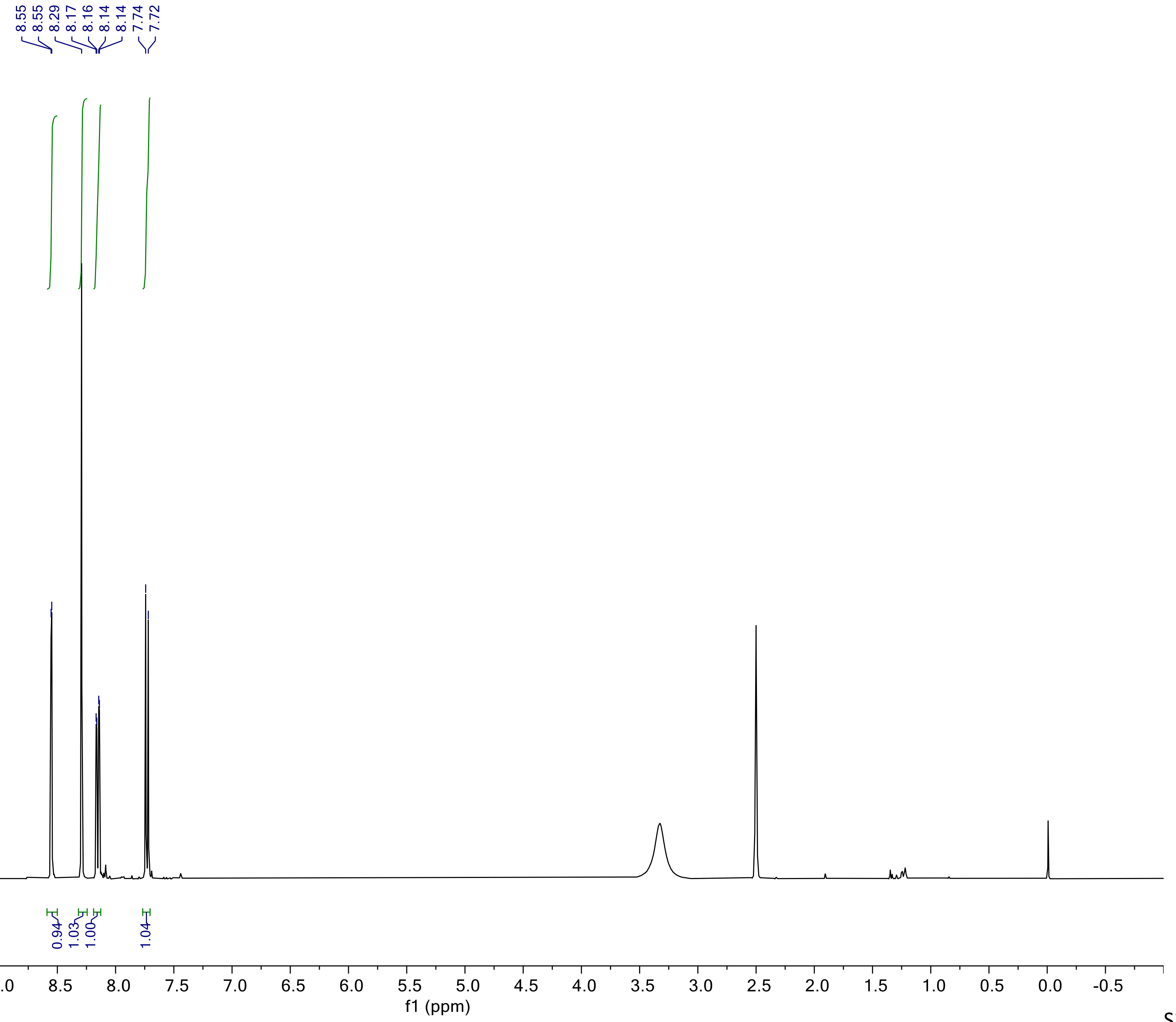

$^{13}C\{^{1}H\}$ NMR (101 MHz, DMSO-d_6)

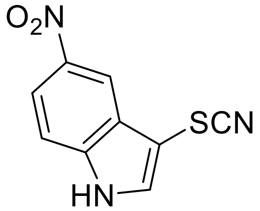

5r

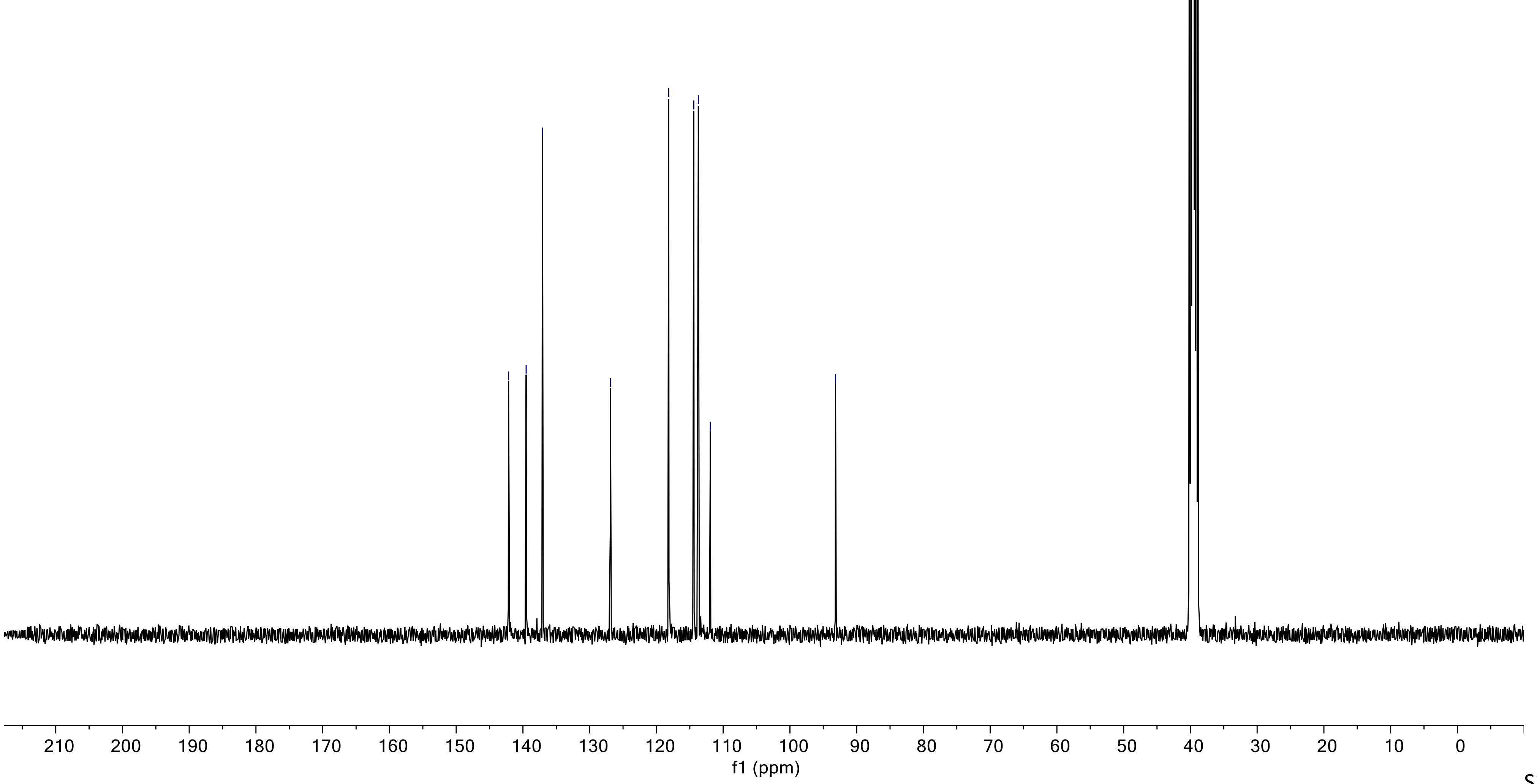

^{1}H NMR (400 MHz, DMSO-d_6)

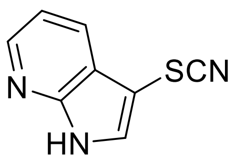

5s

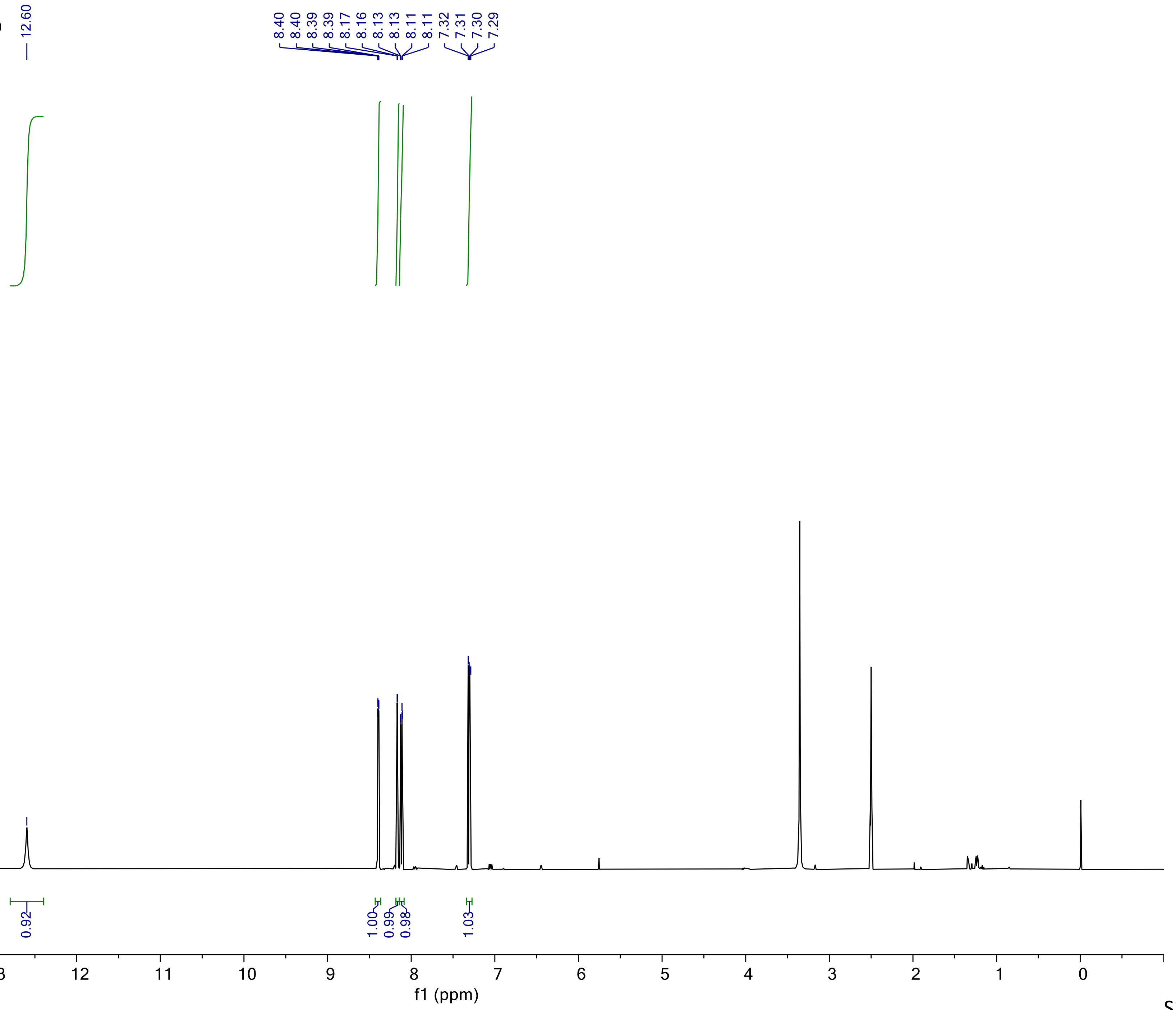

$^{13}C\{^1H\}$ NMR (101 MHz, DMSO-d_6)

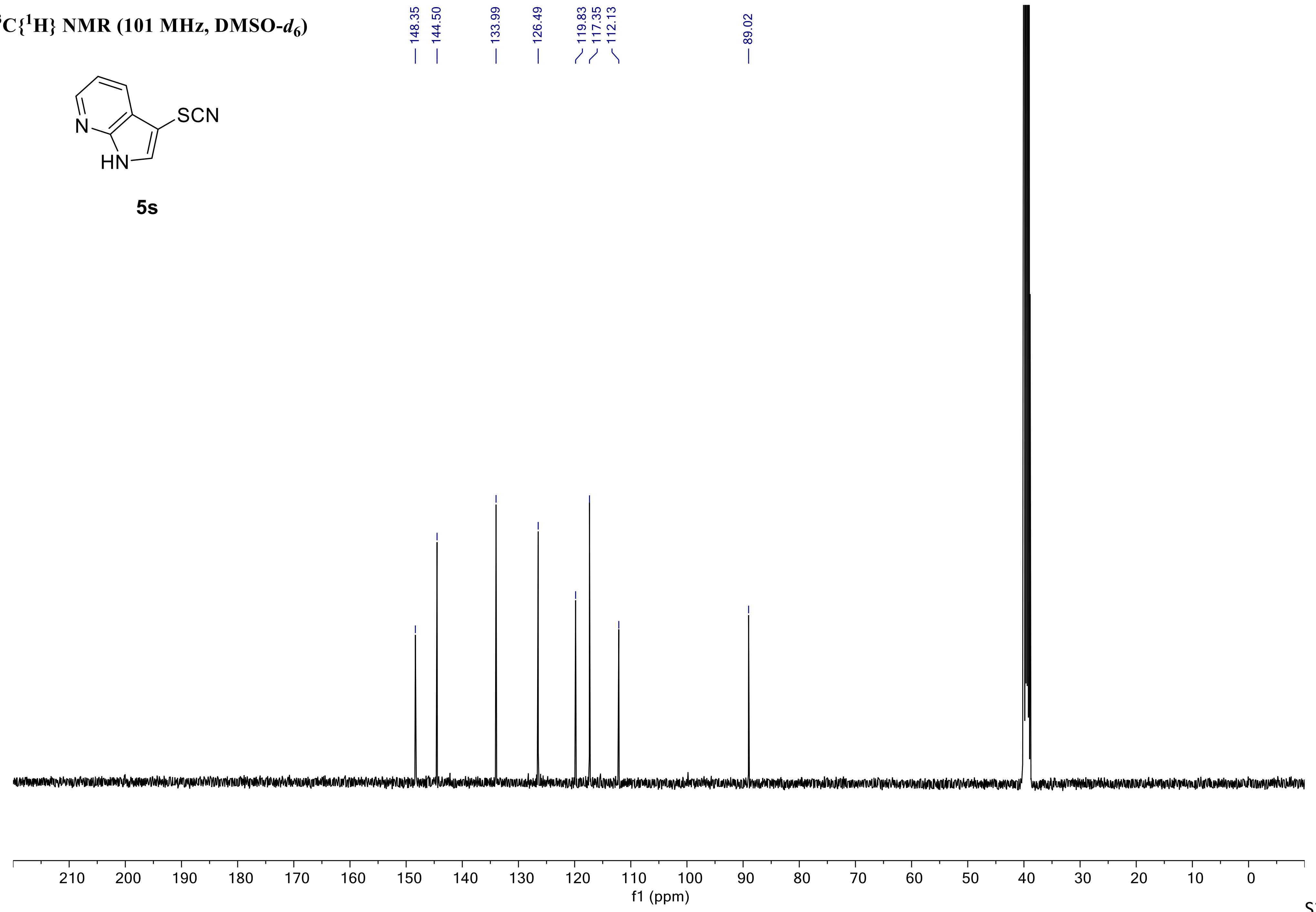

5s

^{1}H NMR (400 MHz, $CDCl_3$)

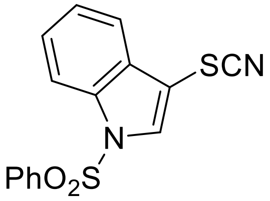

5t

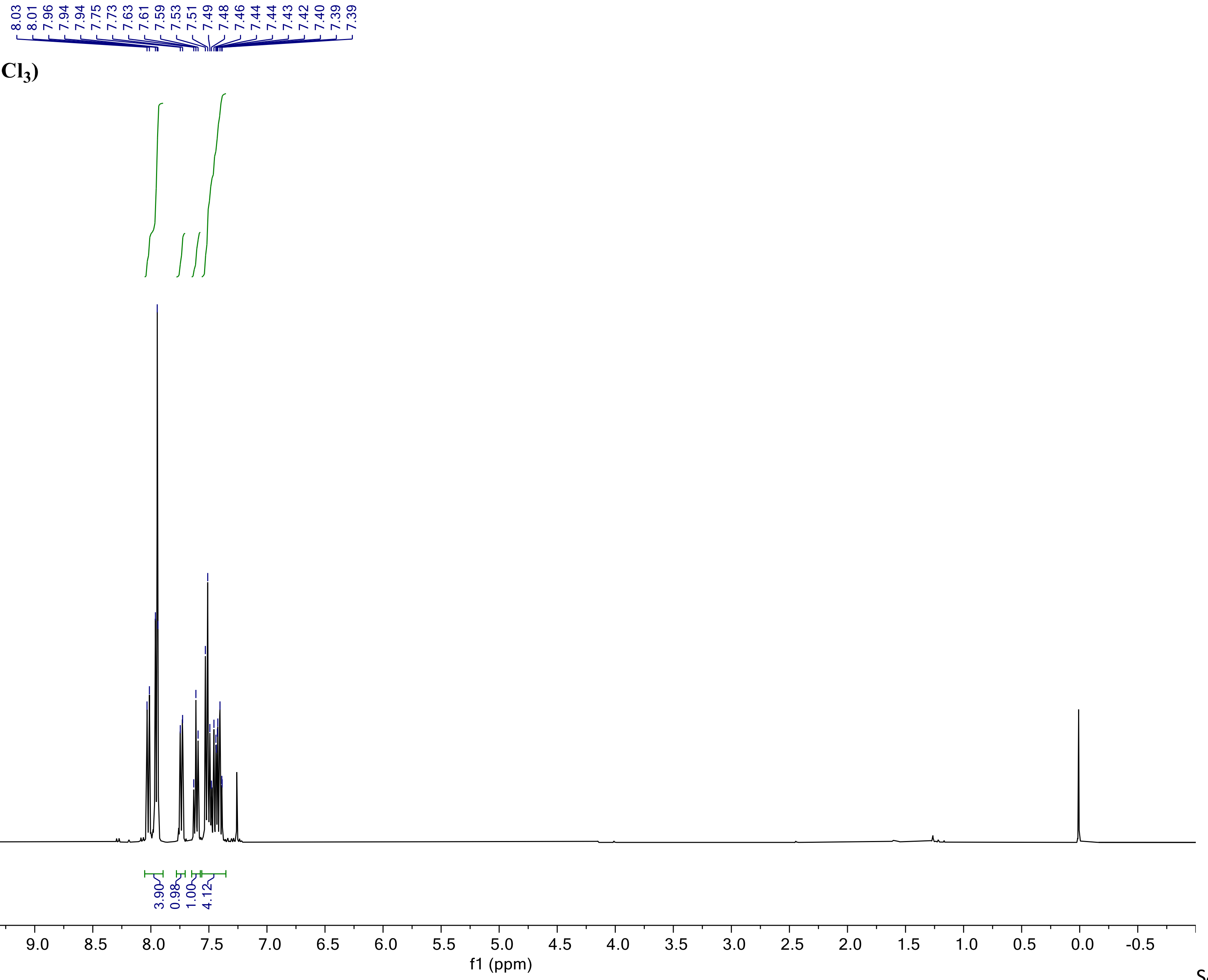

$^{13}C\{^{1}H\}$ NMR (101 MHz, $CDCl_3$)

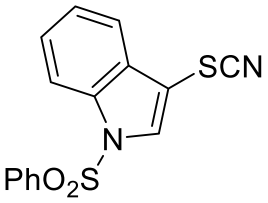

5t

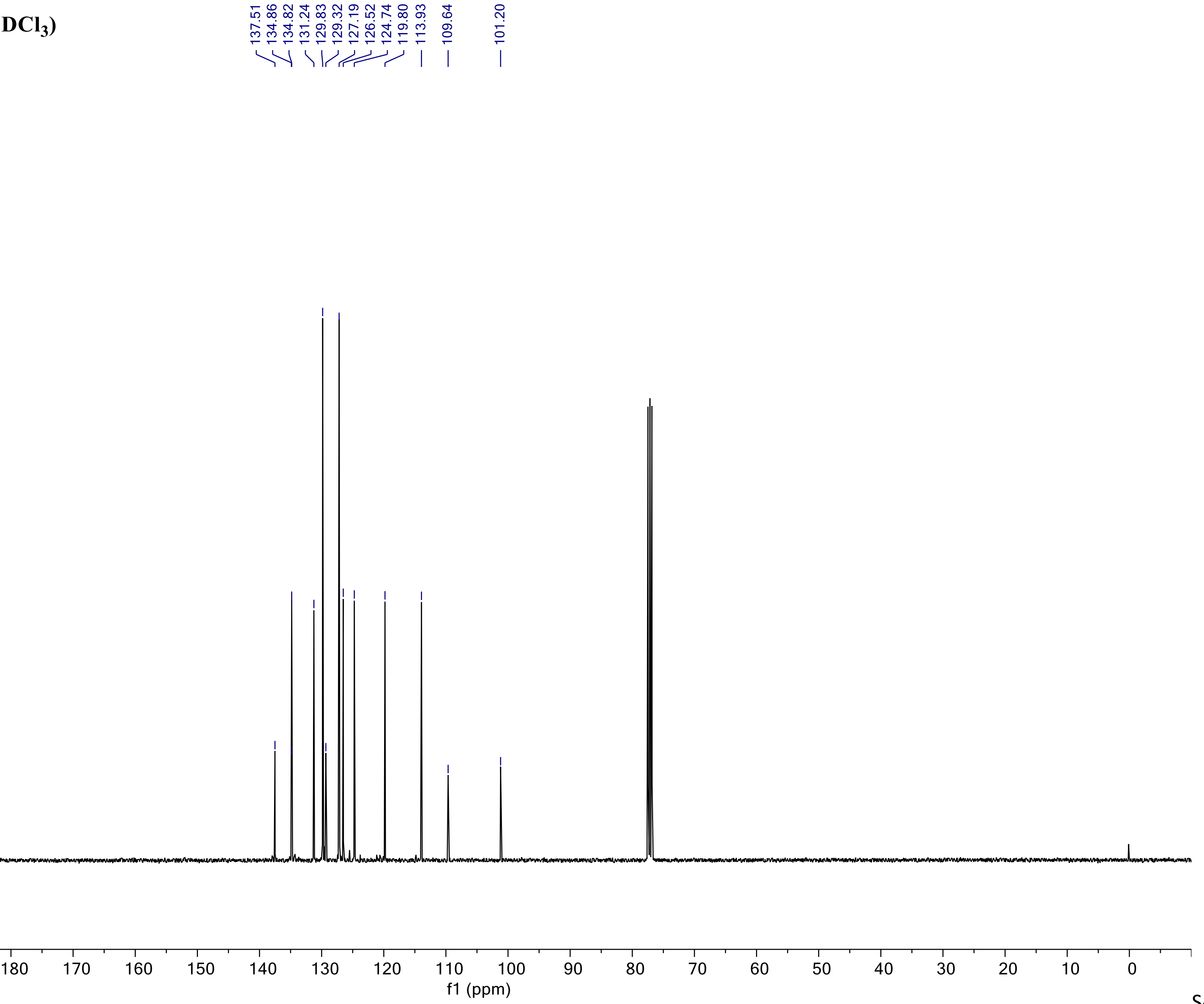

^{1}H NMR (400 MHz, $CDCl_3$)

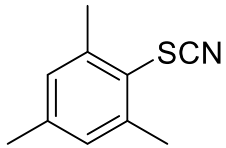

5u

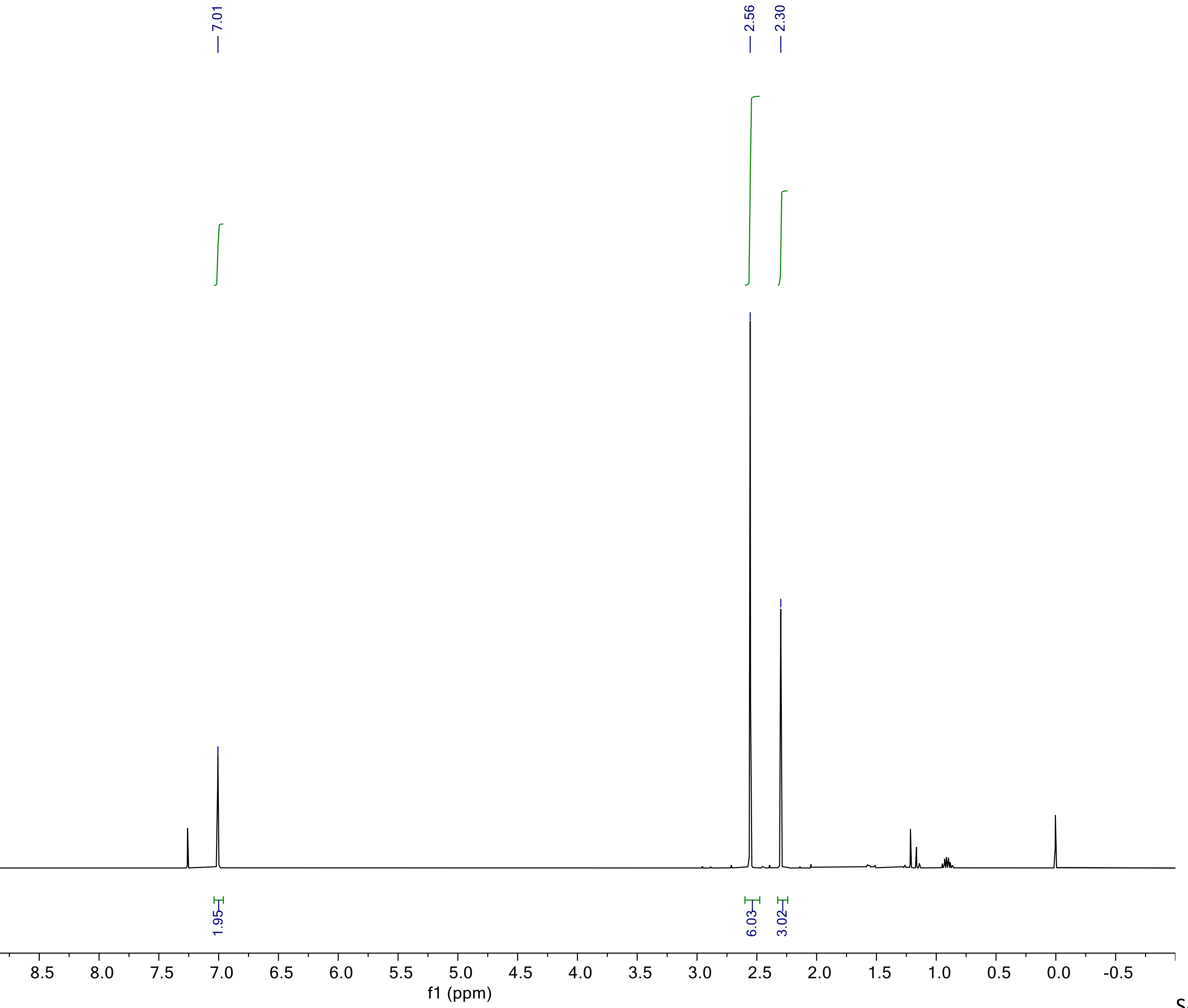

$^{13}C\{^1H\}$ NMR (101 MHz, $CDCl_3$)

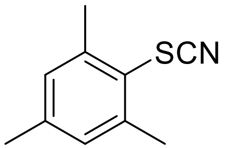

5u

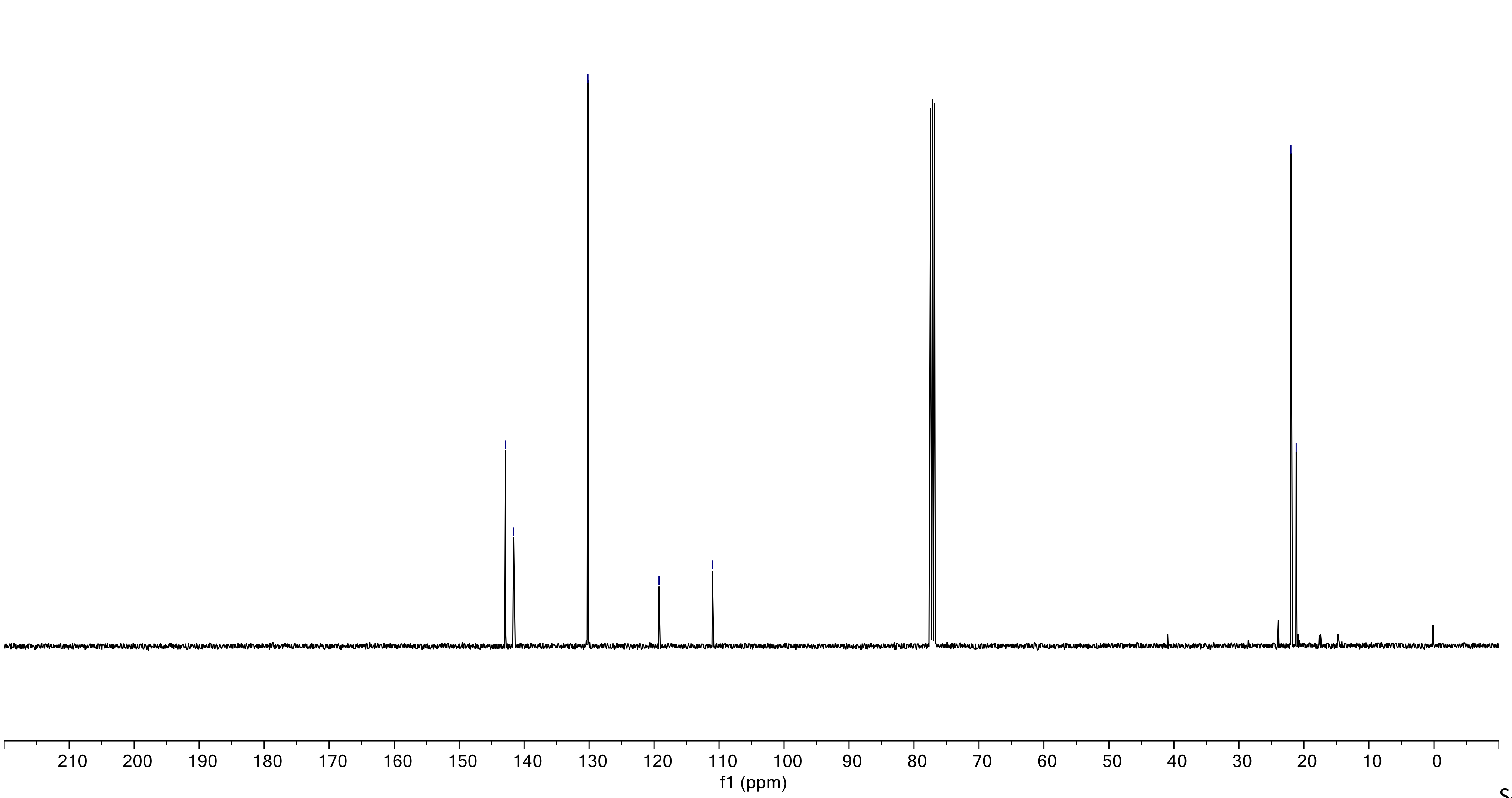

^{1}H NMR (400 MHz, $CDCl_3$)

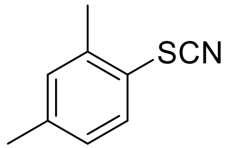

5v

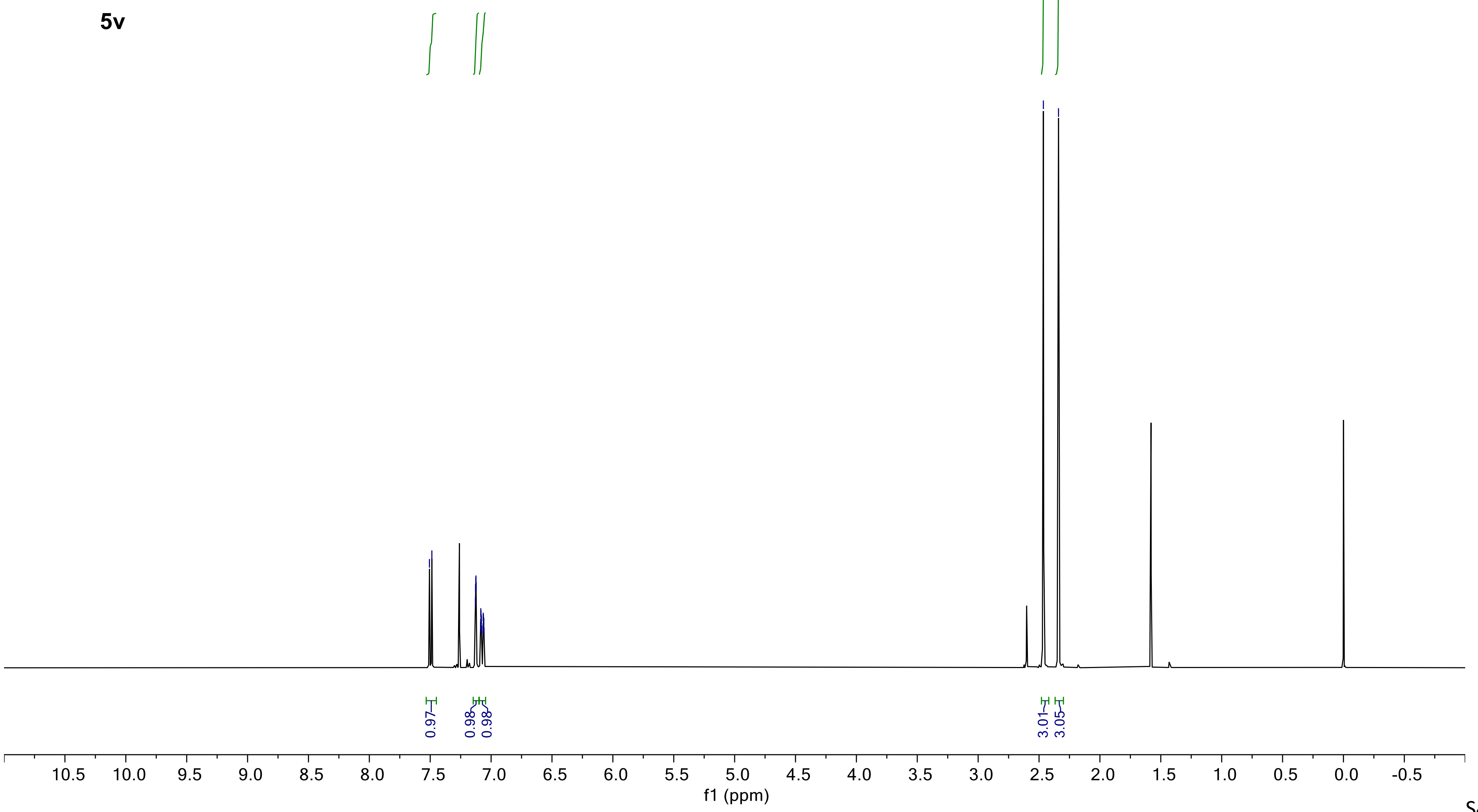

$^{13}C\{^1H\}$ NMR (101 MHz, $CDCl_3$)

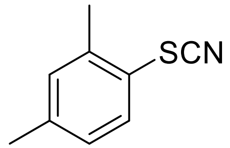

5v

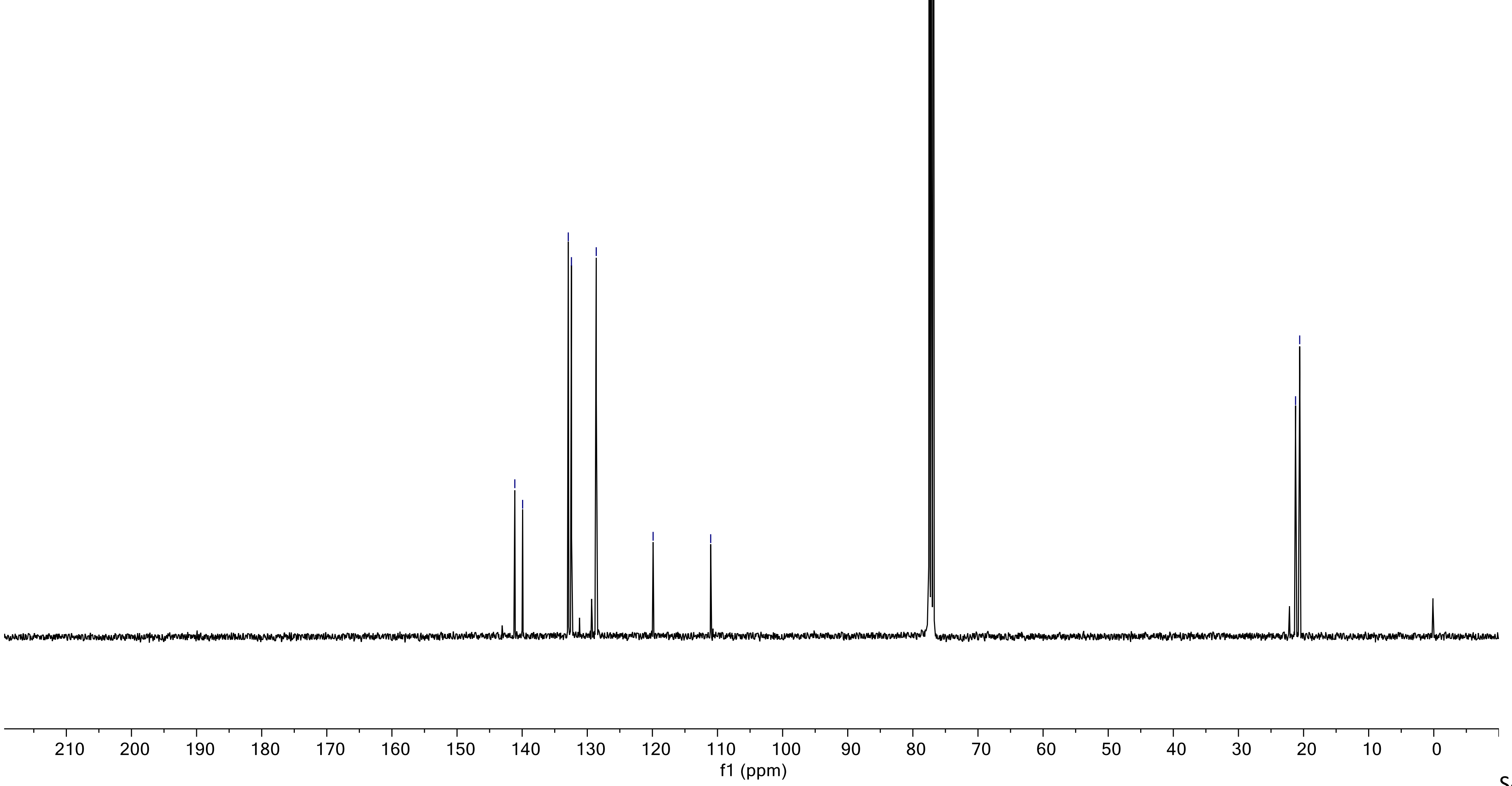

^{1}H NMR (400 MHz, $CDCl_3$)

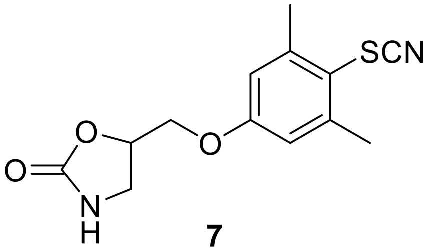

7

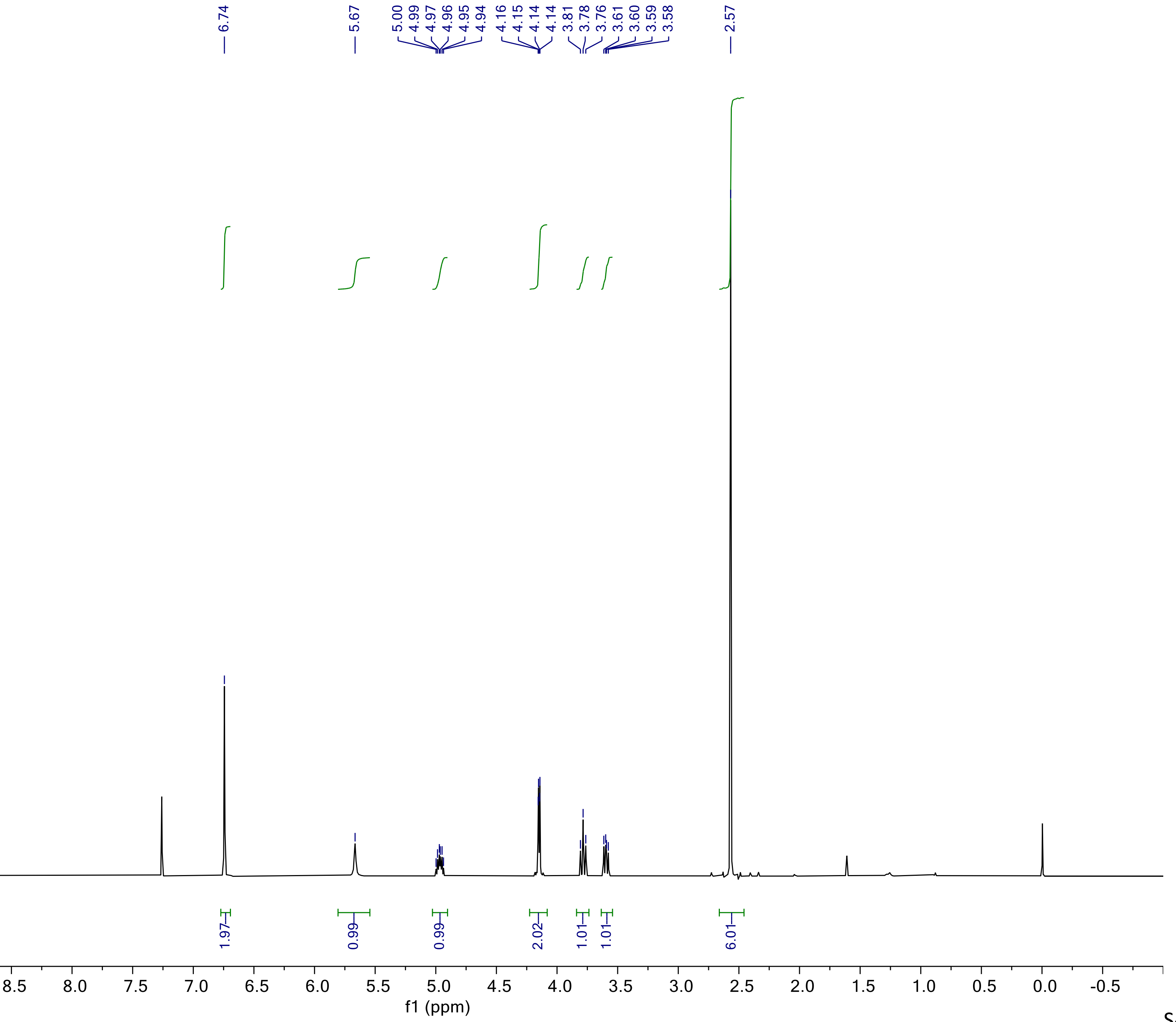

$^{13}C\{^{1}H\}$ NMR (101 MHz, $CDCl_3$)

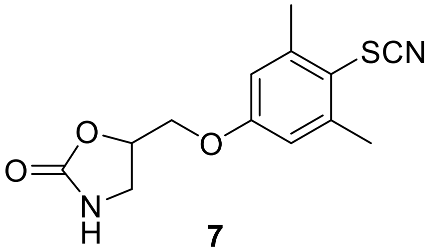

7

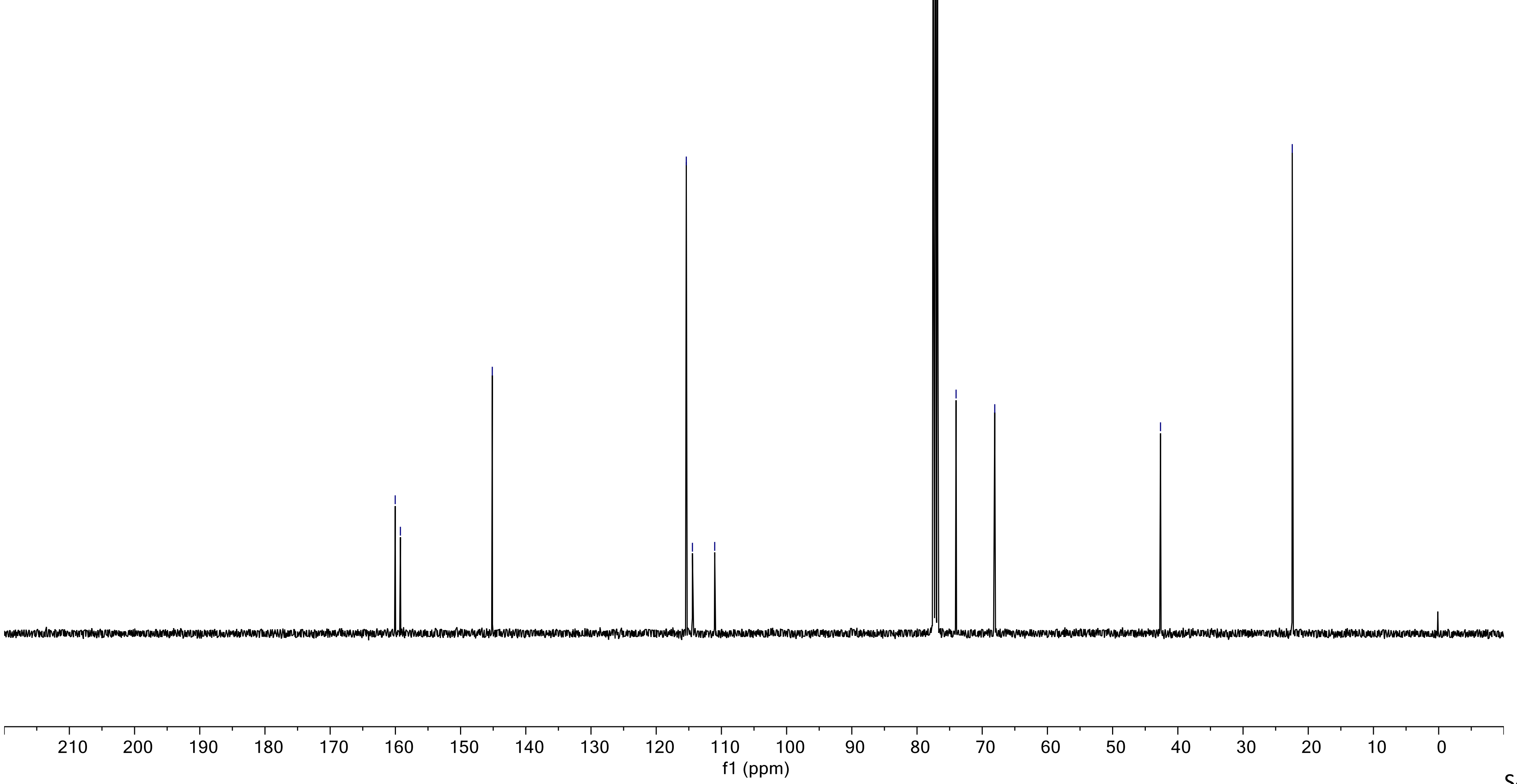

^{1}H NMR (400 MHz, $CDCl_3$)

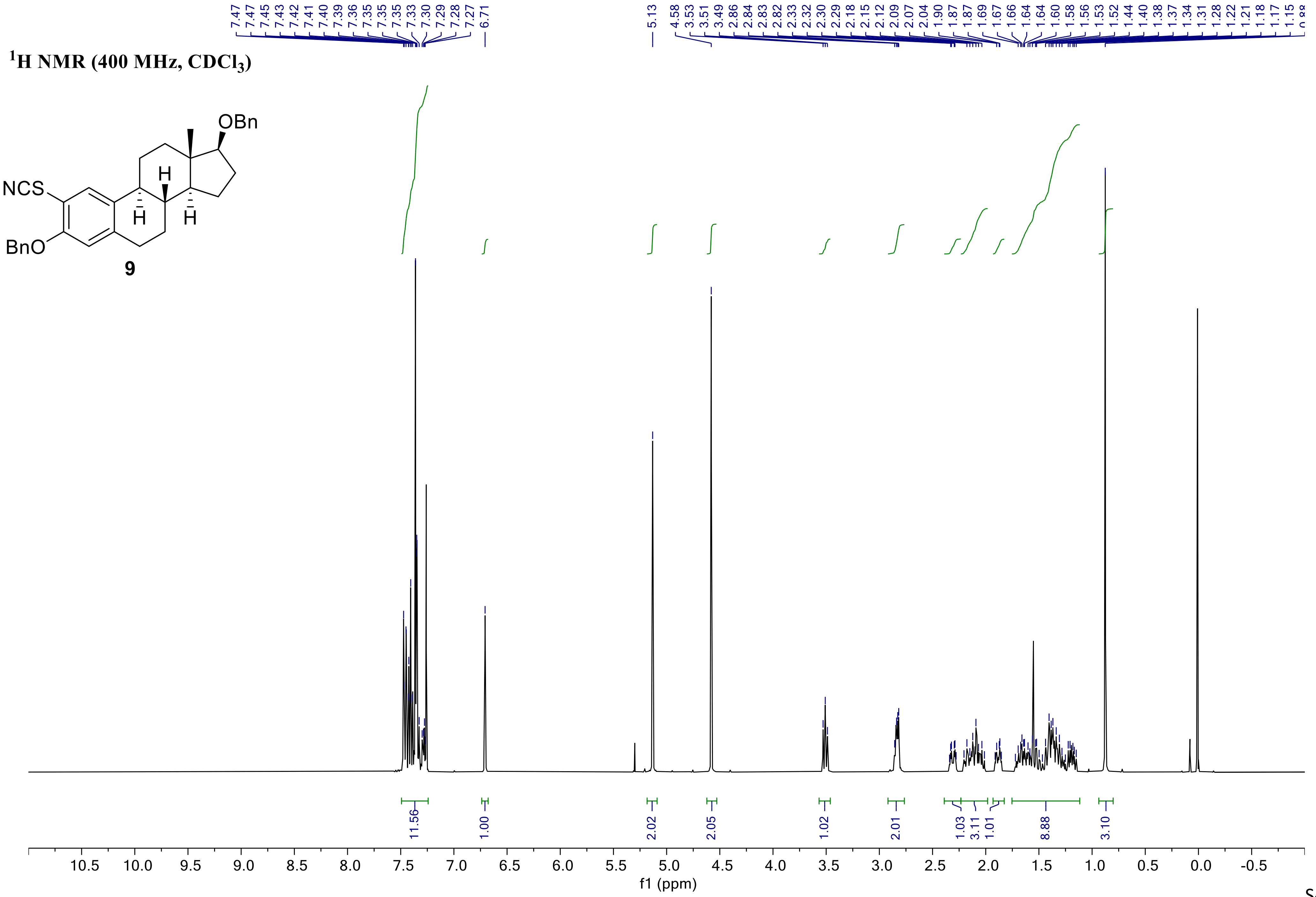

$^{13}C\{^{1}H\}$ NMR (101 MHz, $CDCl_3$)

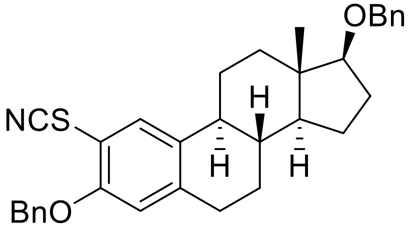

9

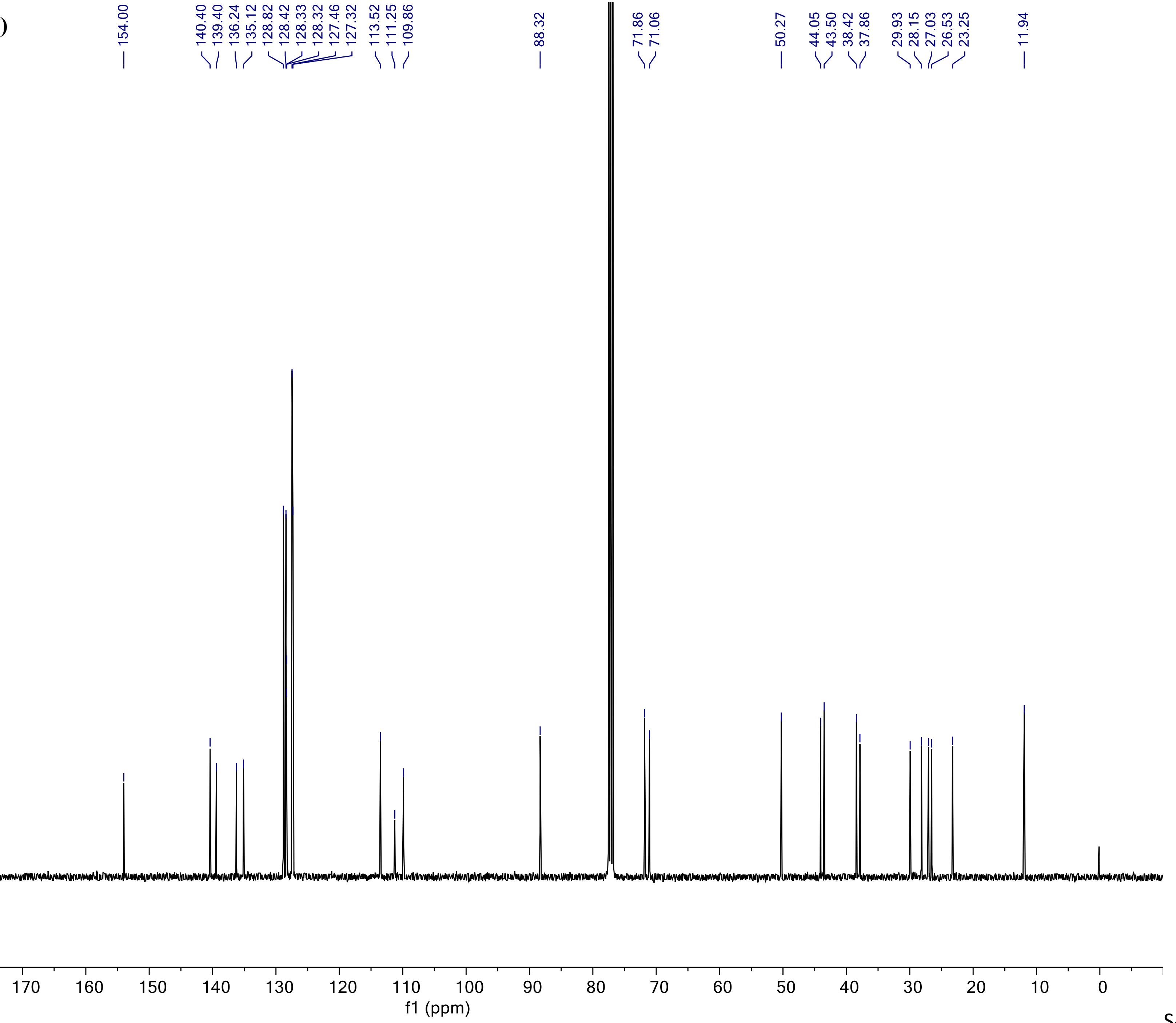

^{1}H NMR (400 MHz, $CDCl_3$)

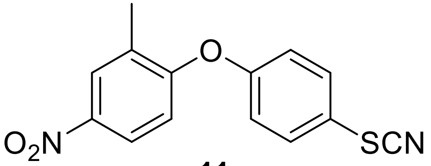

11

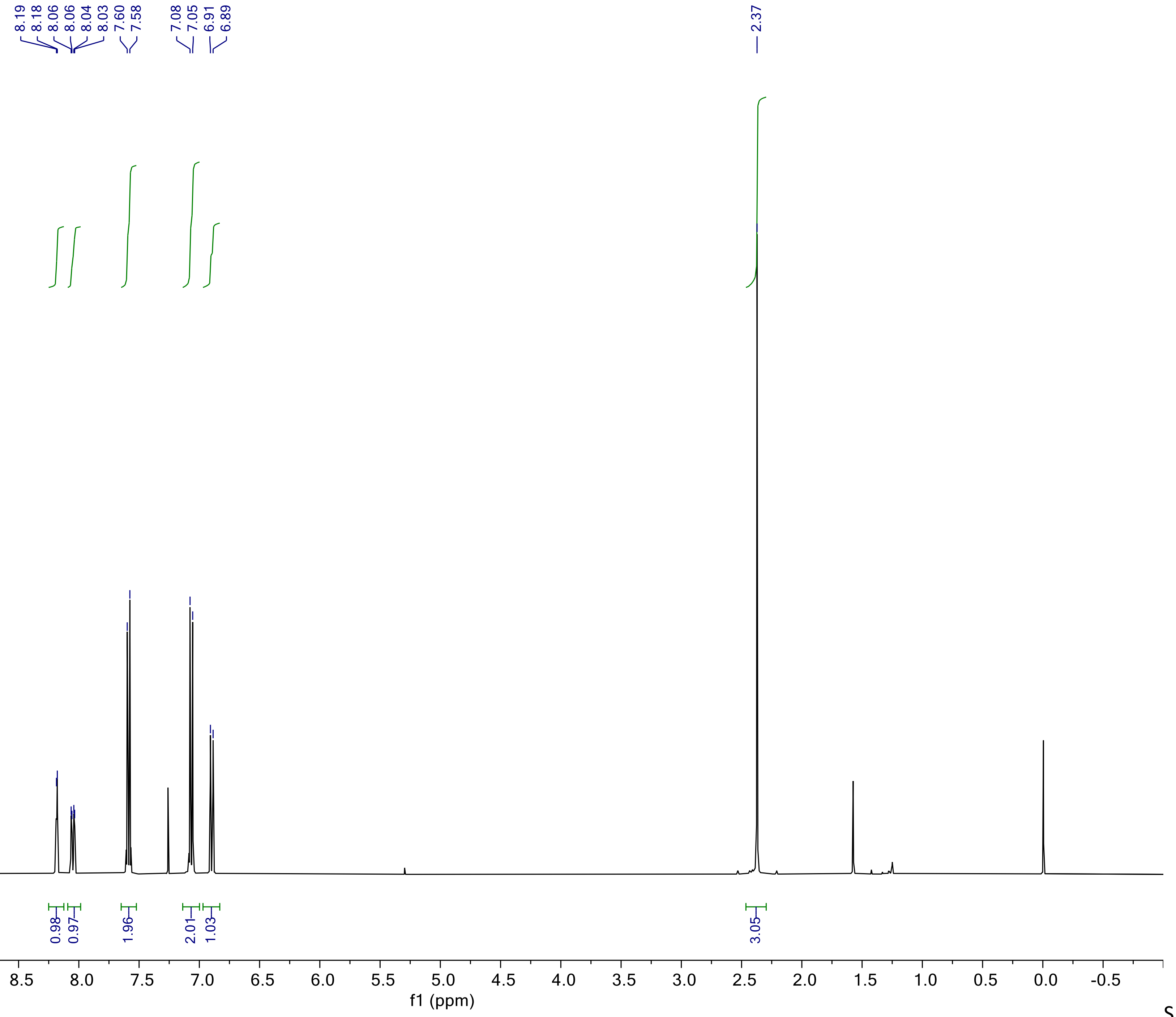

$^{13}C\{^{1}H\}$ NMR (101 MHz, $CDCl_3$)

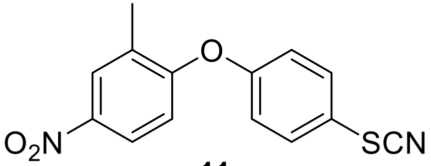

11

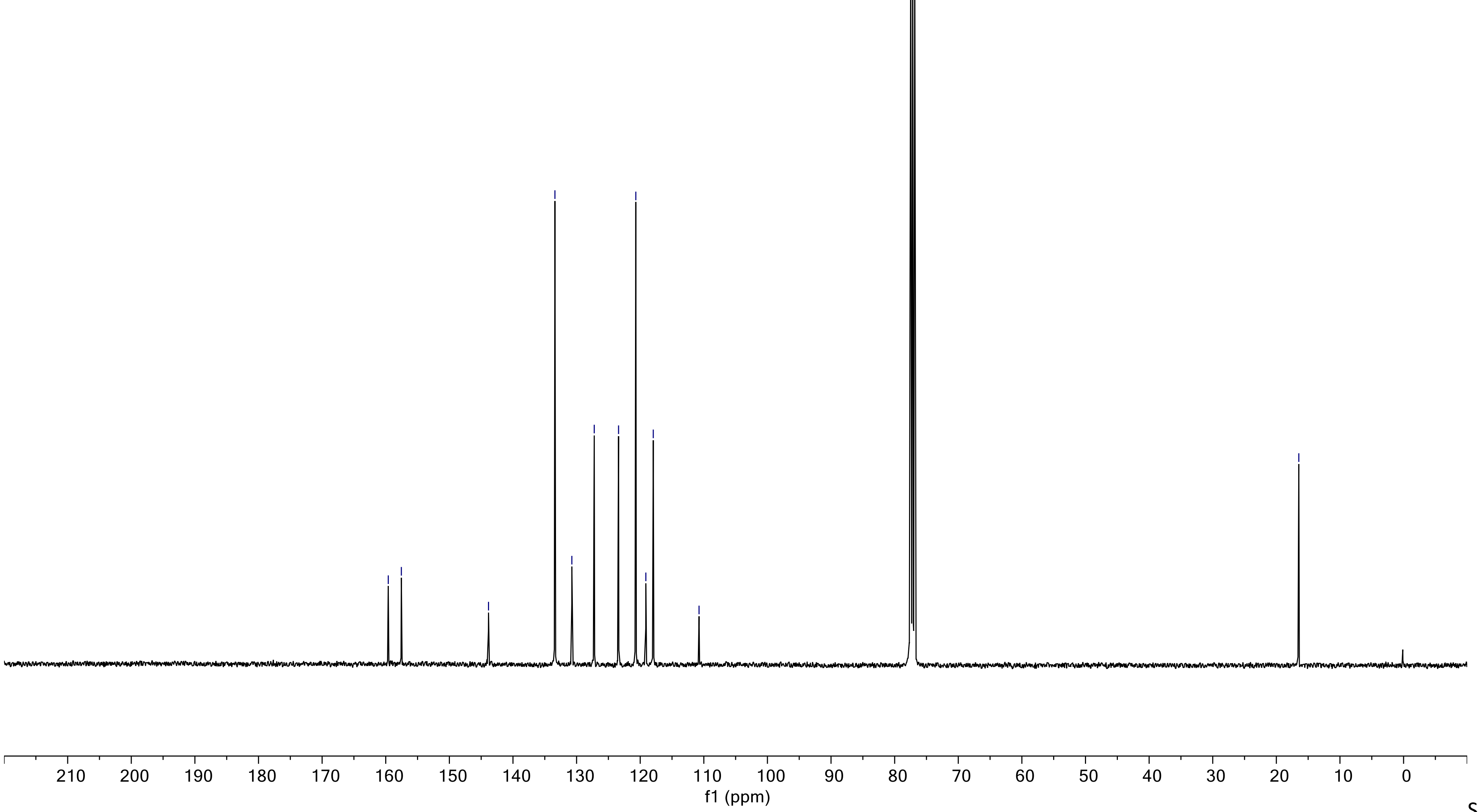

^{1}H NMR (400 MHz, $CDCl_3$)

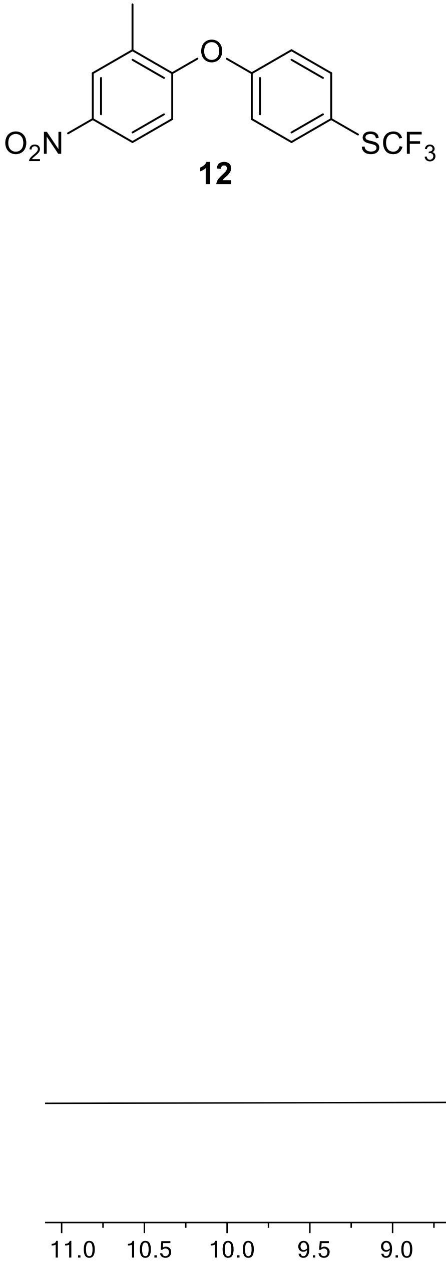

12

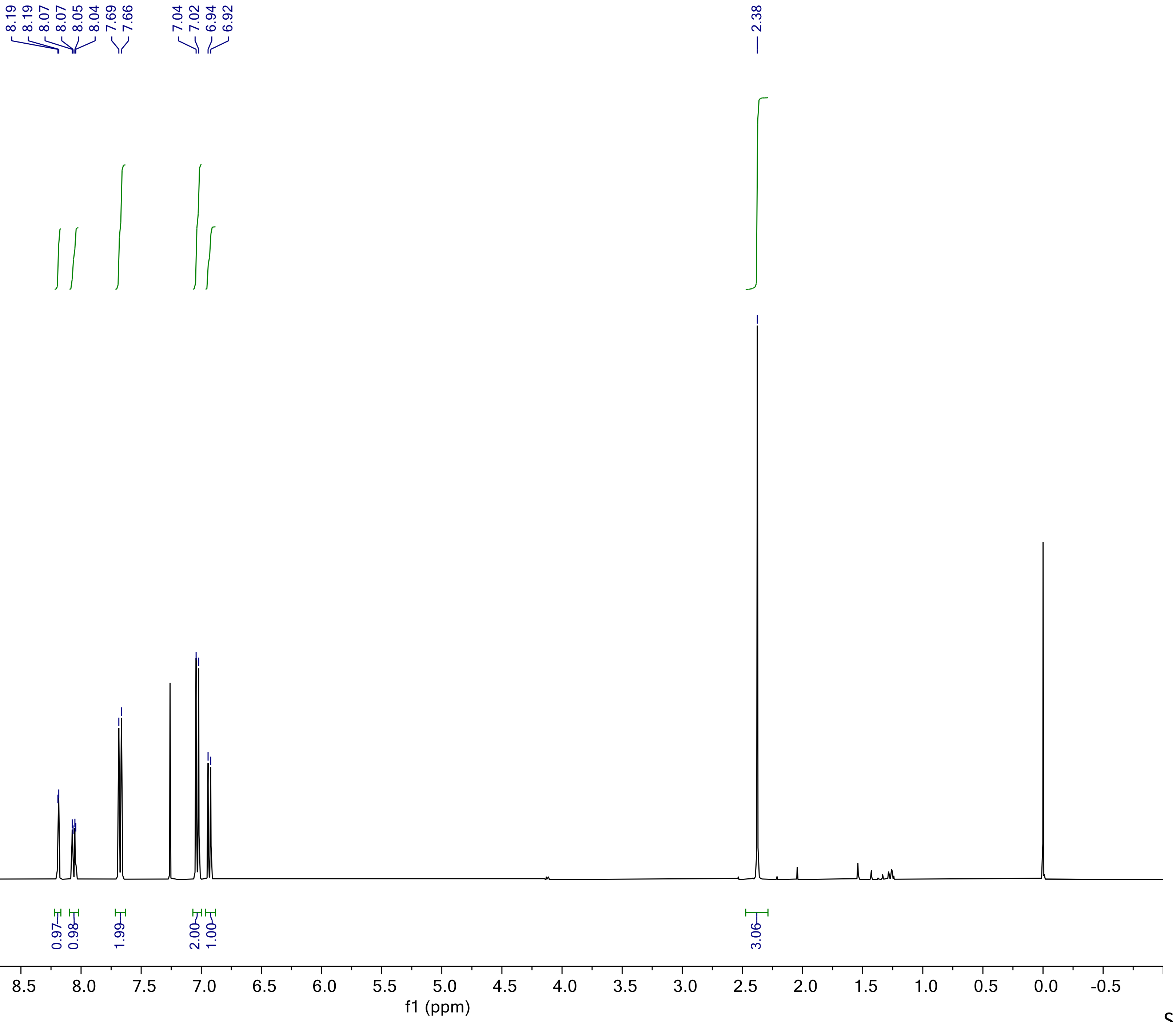

$^{13}C\{^{1}H\}$ NMR (101 MHz, $CDCl_3$)

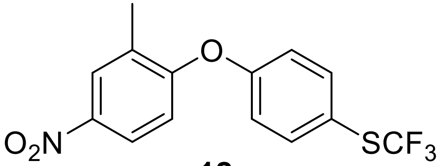

12

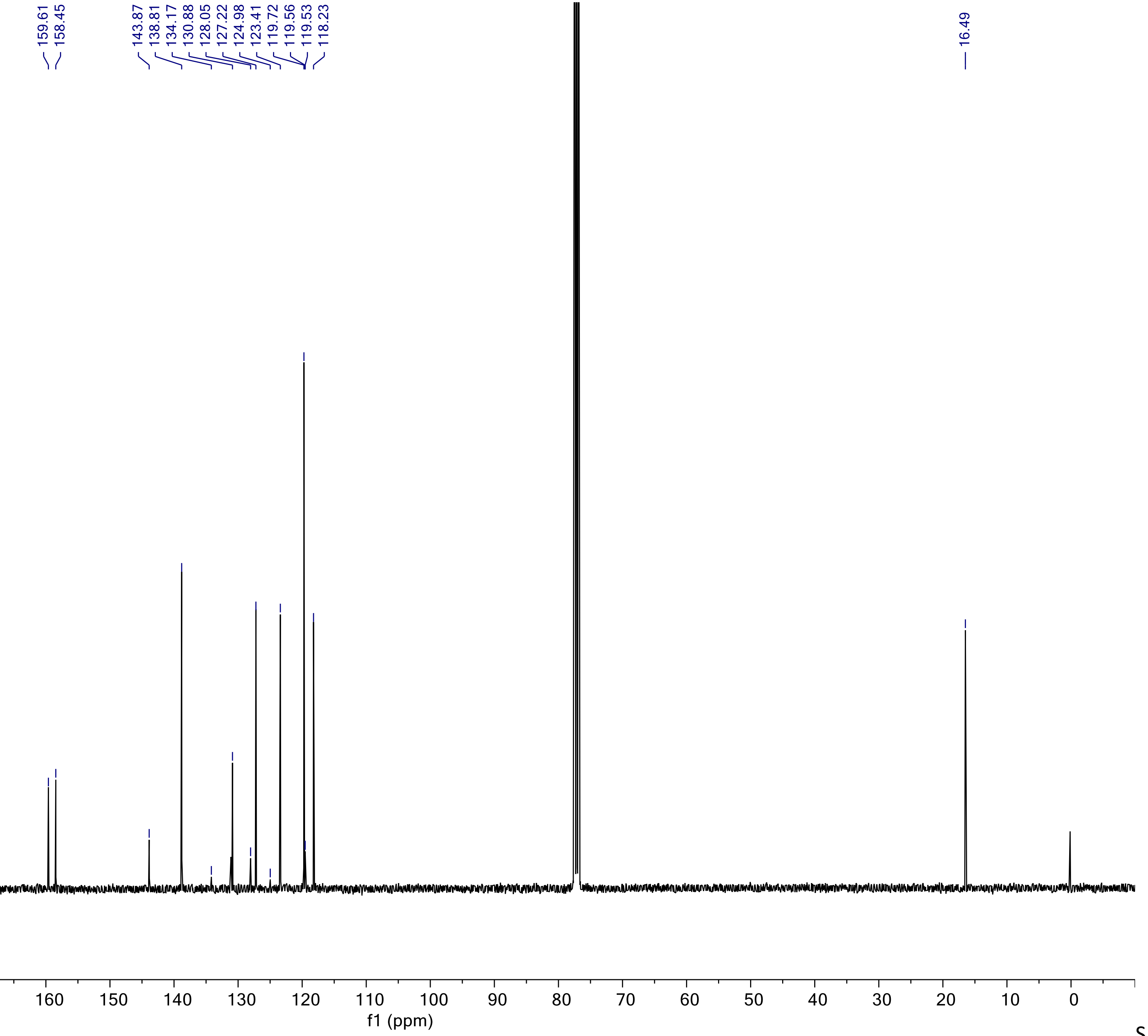

^{1}H NMR (400 MHz, $CDCl_3$)

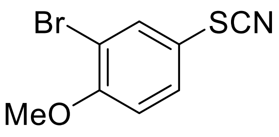

14

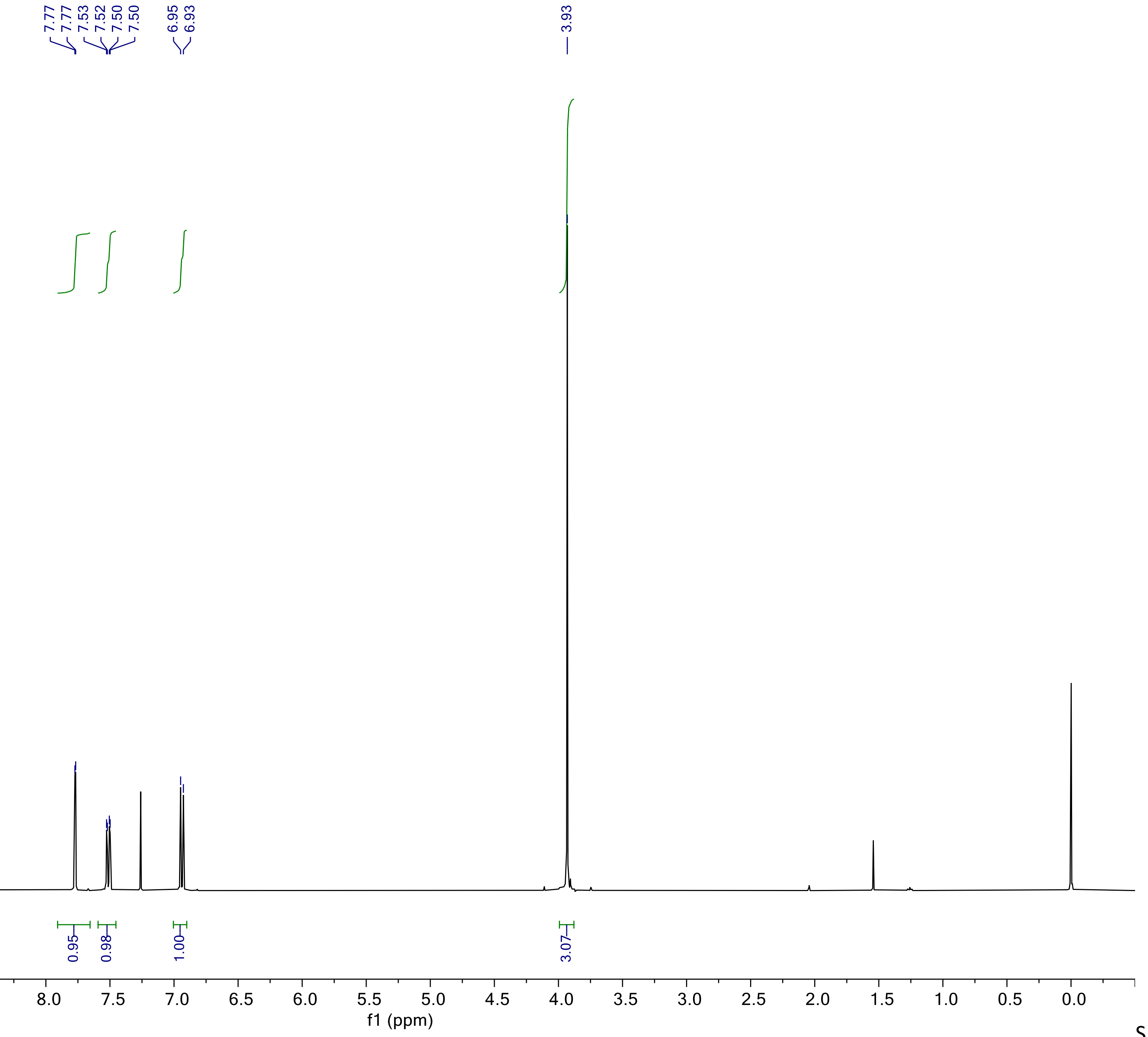

$^{13}C\{^{1}H\}$ NMR (101 MHz, $CDCl_3$)

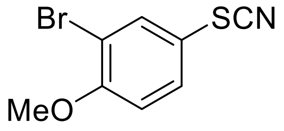

14

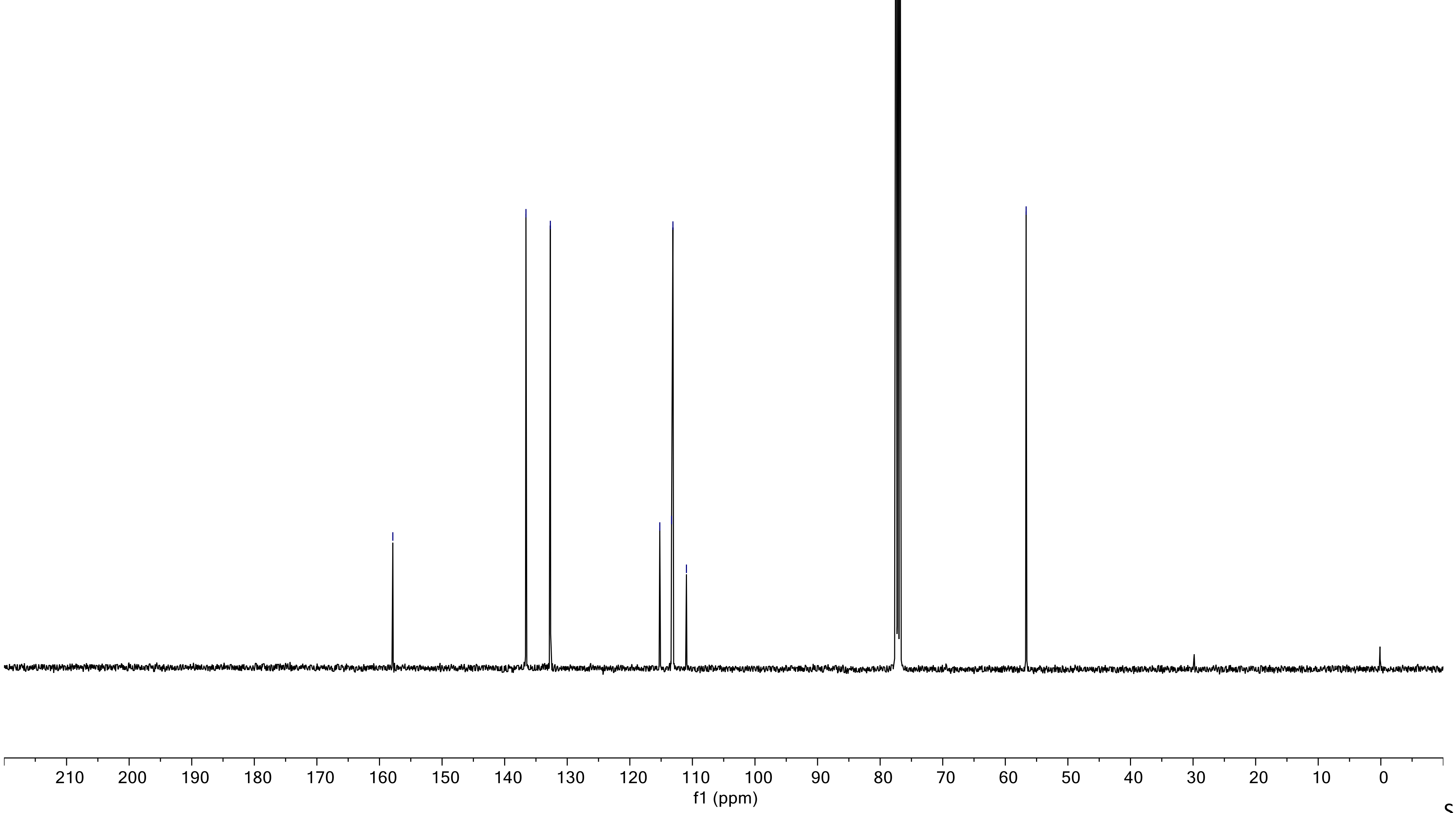

^{1}H NMR (400 MHz, $CDCl_3$)

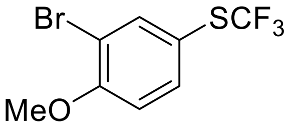

15

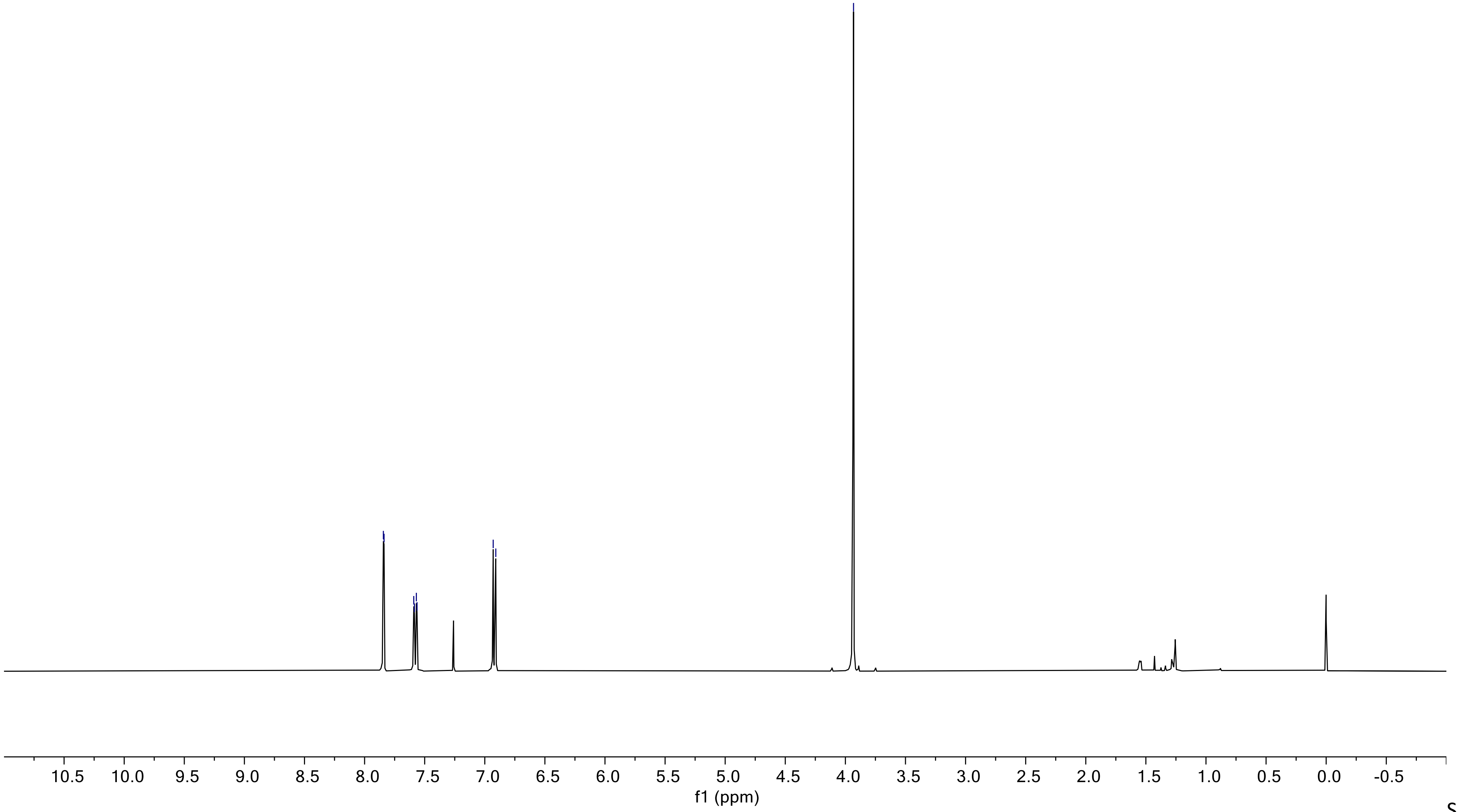

$^{13}C\{^1H\}$ NMR (101 MHz, $CDCl_3$)

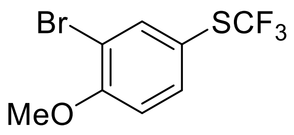

15

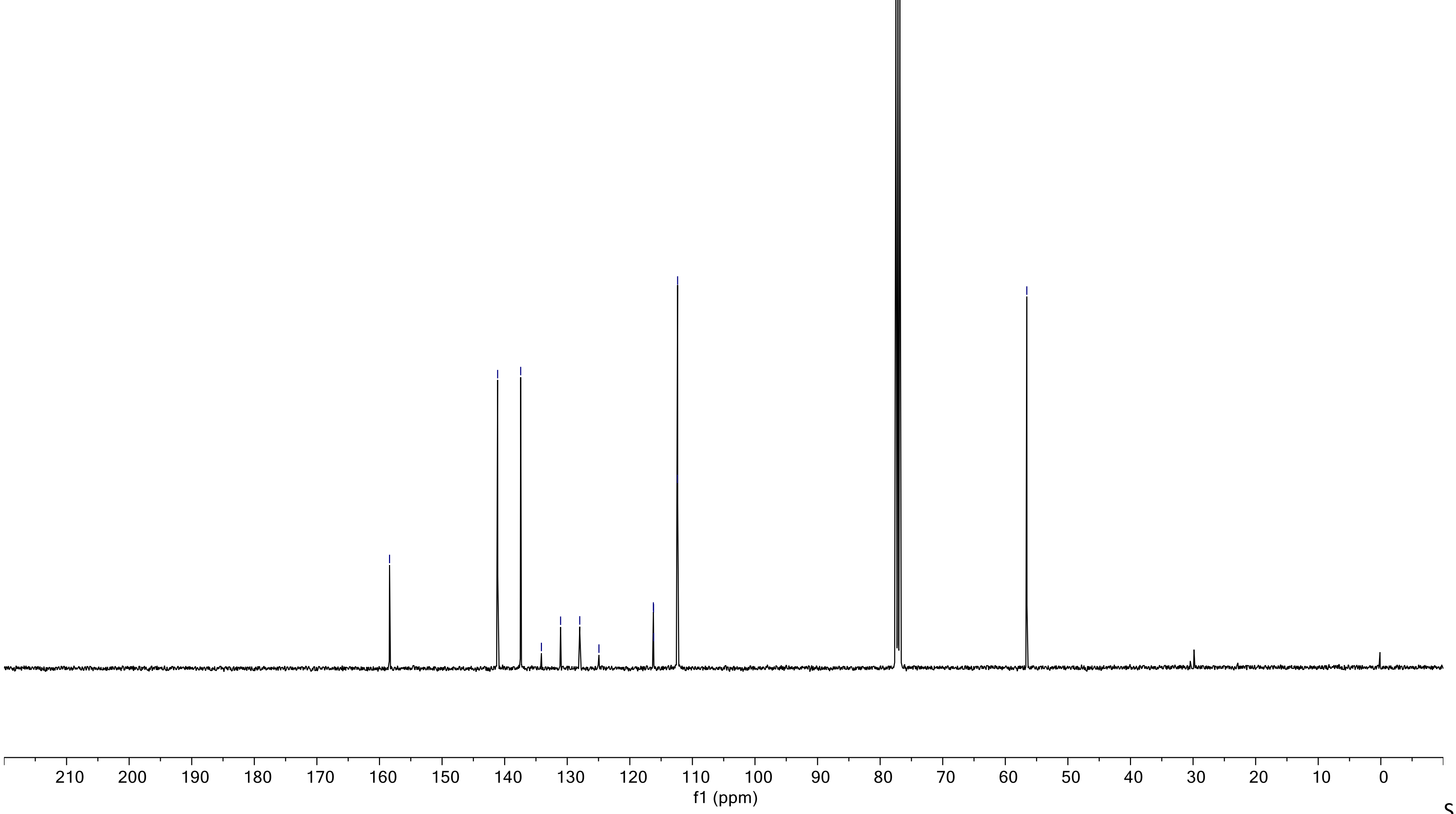

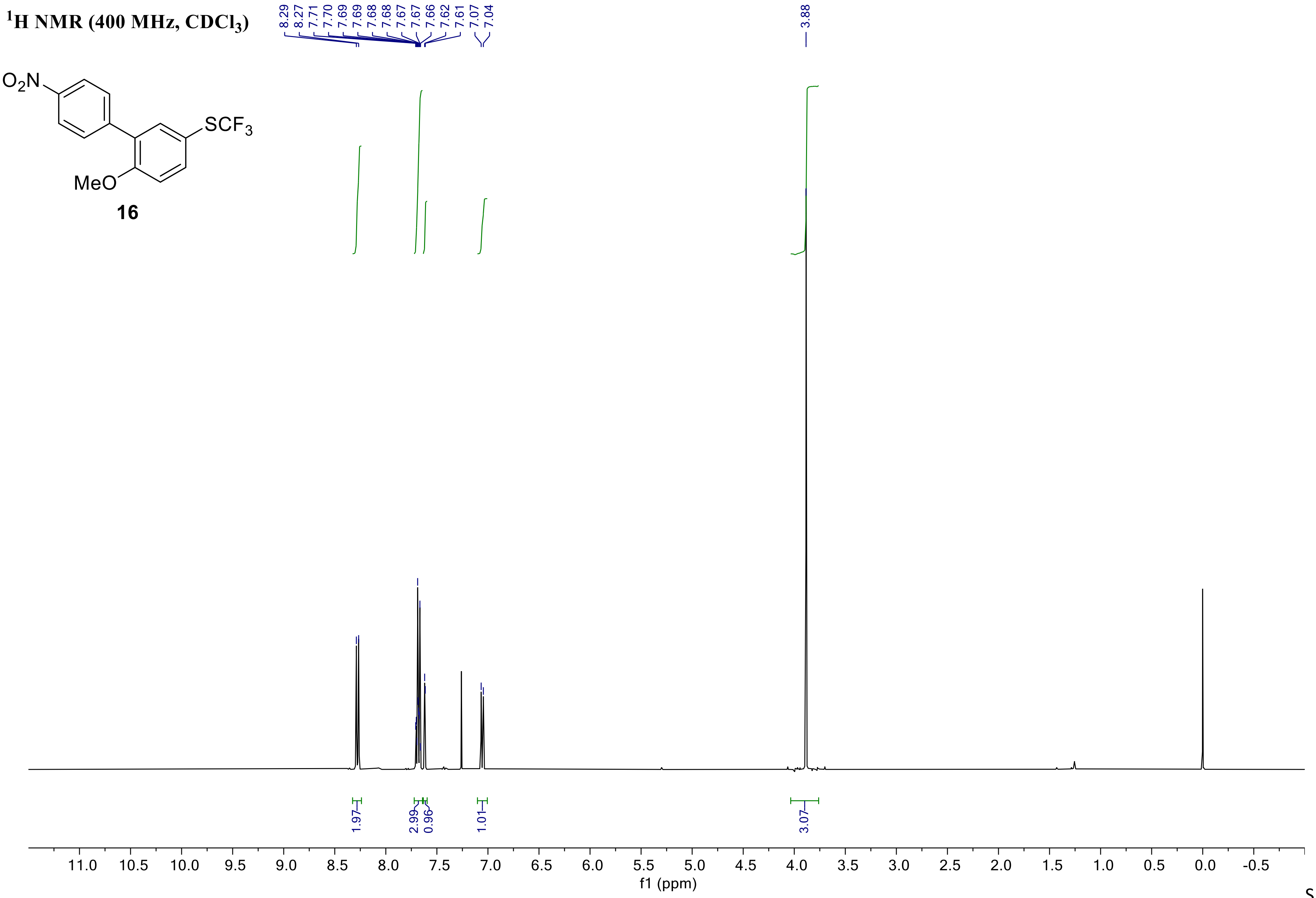

^{1}H NMR (400 MHz, CDCl$_3$)
8.29
8.27
7.71
7.70
7.69
7.69
7.68
7.68
7.67
7.67
7.66
7.62
7.61
7.07
7.04
3.88
O2N
SCF3
MeO
16
1.97
2.99
0.96
1.01
3.07
11.0
10.5
10.0
9.5
9.0
8.5
8.0
7.5
7.0
6.5
6.0
5.5
5.0
4.5
4.0
3.5
3.0
2.5
2.0
1.5
1.0
0.5
0.0
-0.5
f1 (ppm)

$^{13}C\{^1H\}$ NMR (101 MHz, $CDCl_3$)

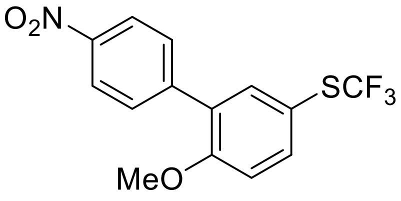

16

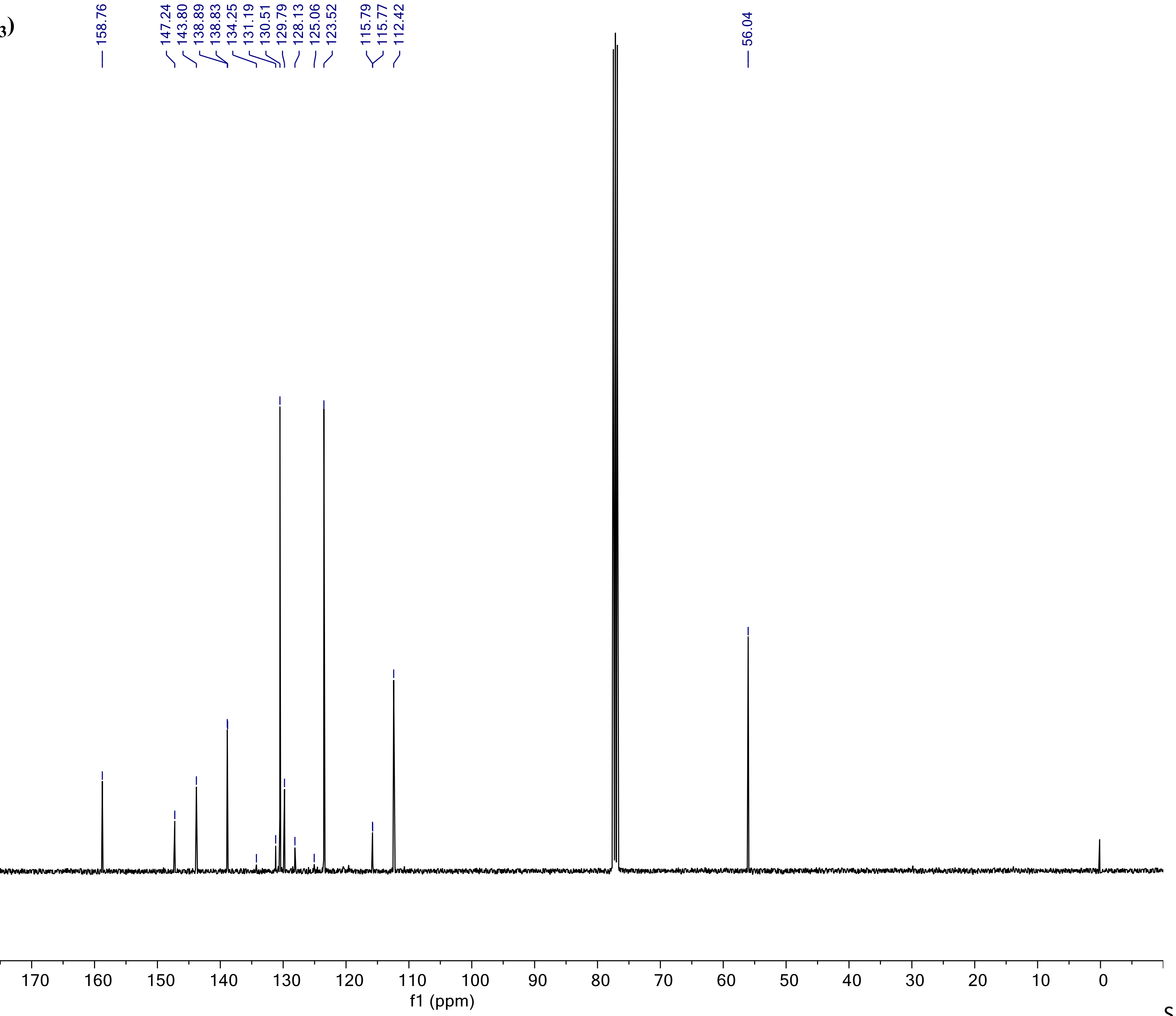

^{1}H NMR (400 MHz, $CDCl_3$)

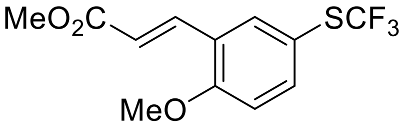

17

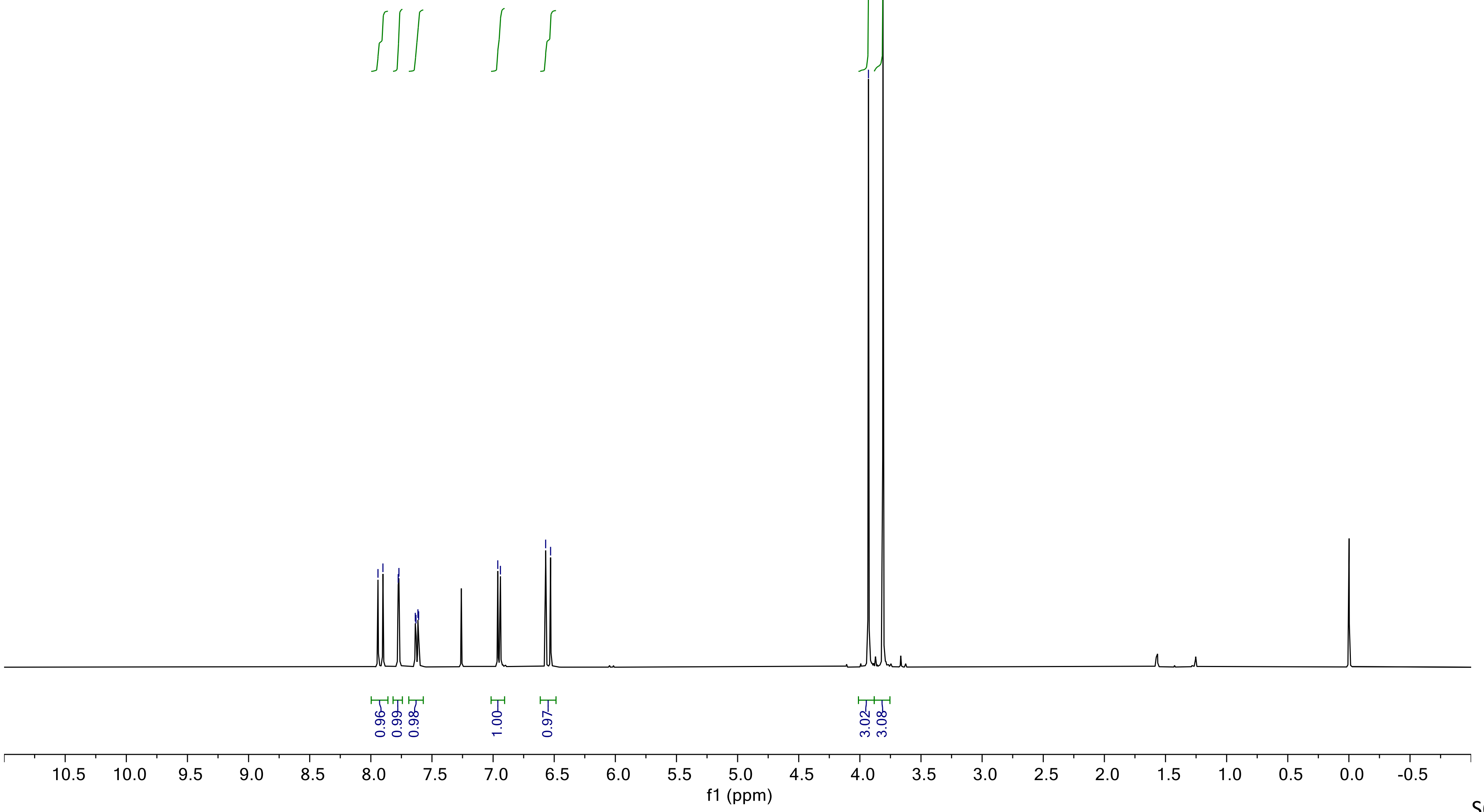

$^{13}C\{^1H\}$ NMR (101 MHz, $CDCl_3$)

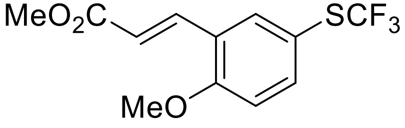

17

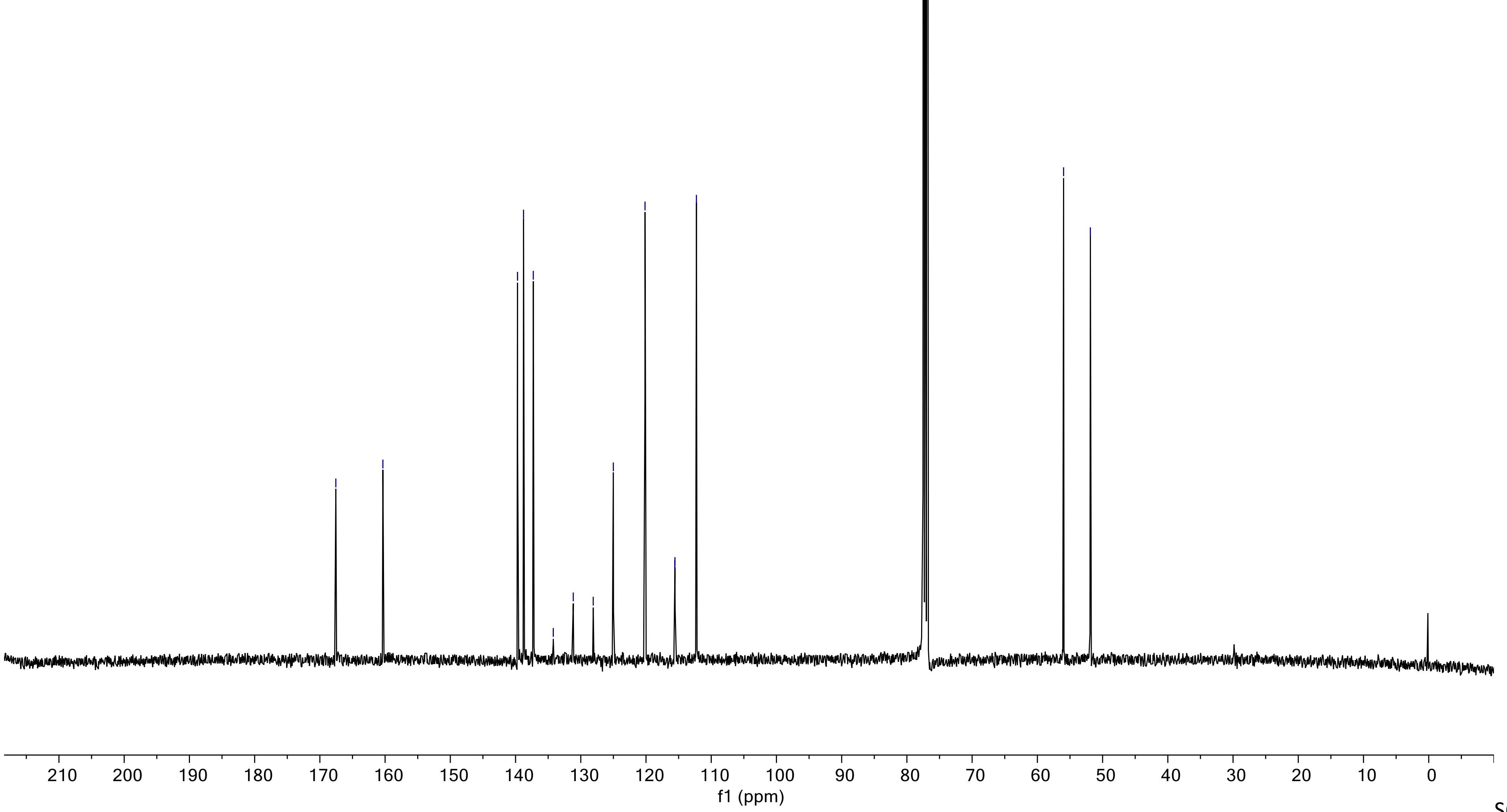

^{1}H NMR (400 MHz, $CDCl_3$)

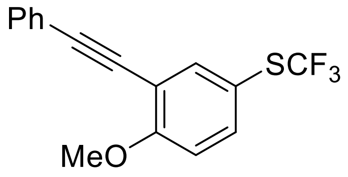

18

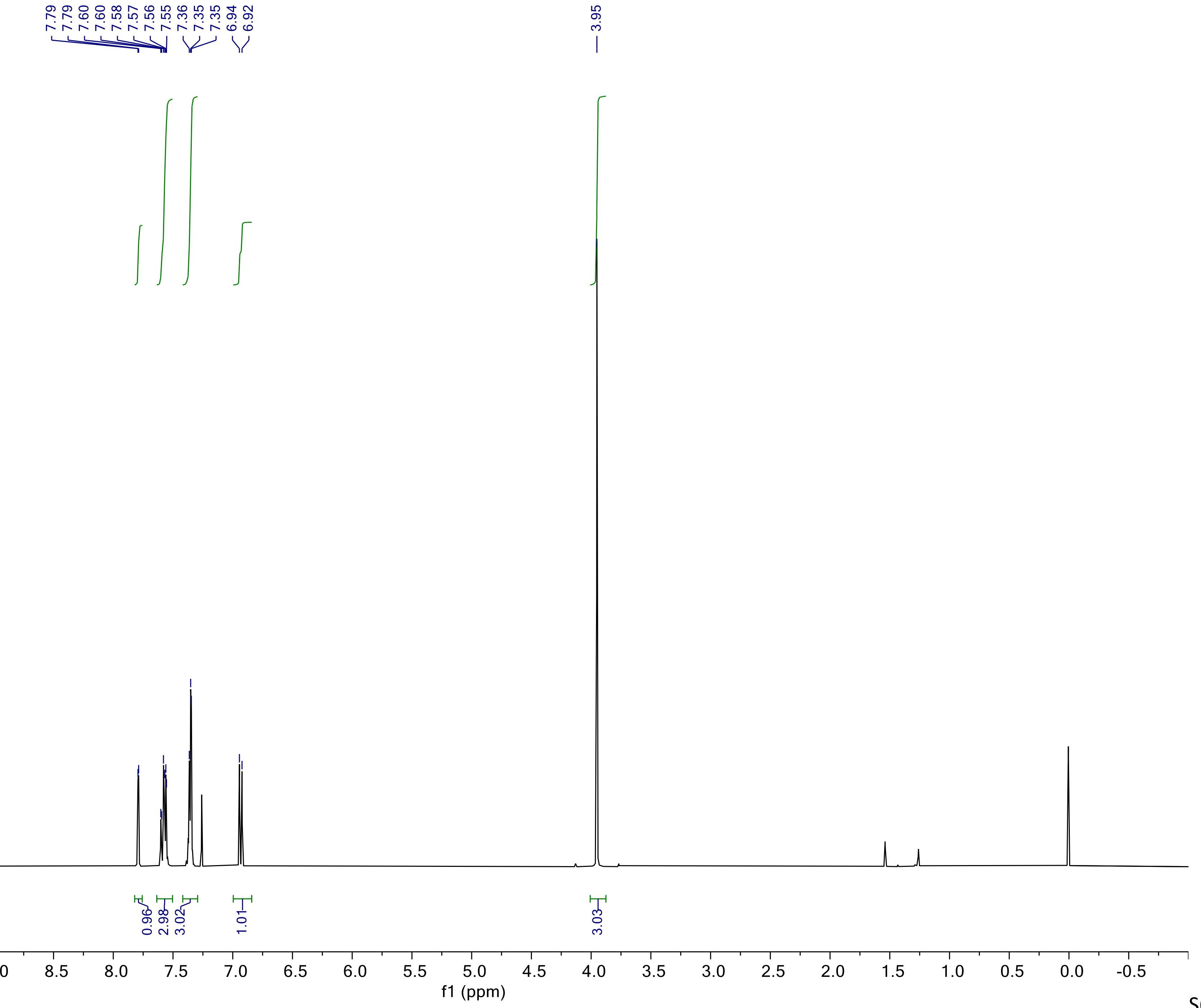

$^{13}C\{^{1}H\}$ NMR (101 MHz, $CDCl_3$)

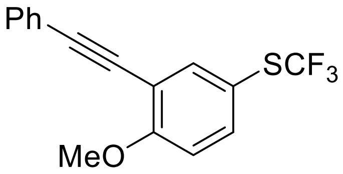

18

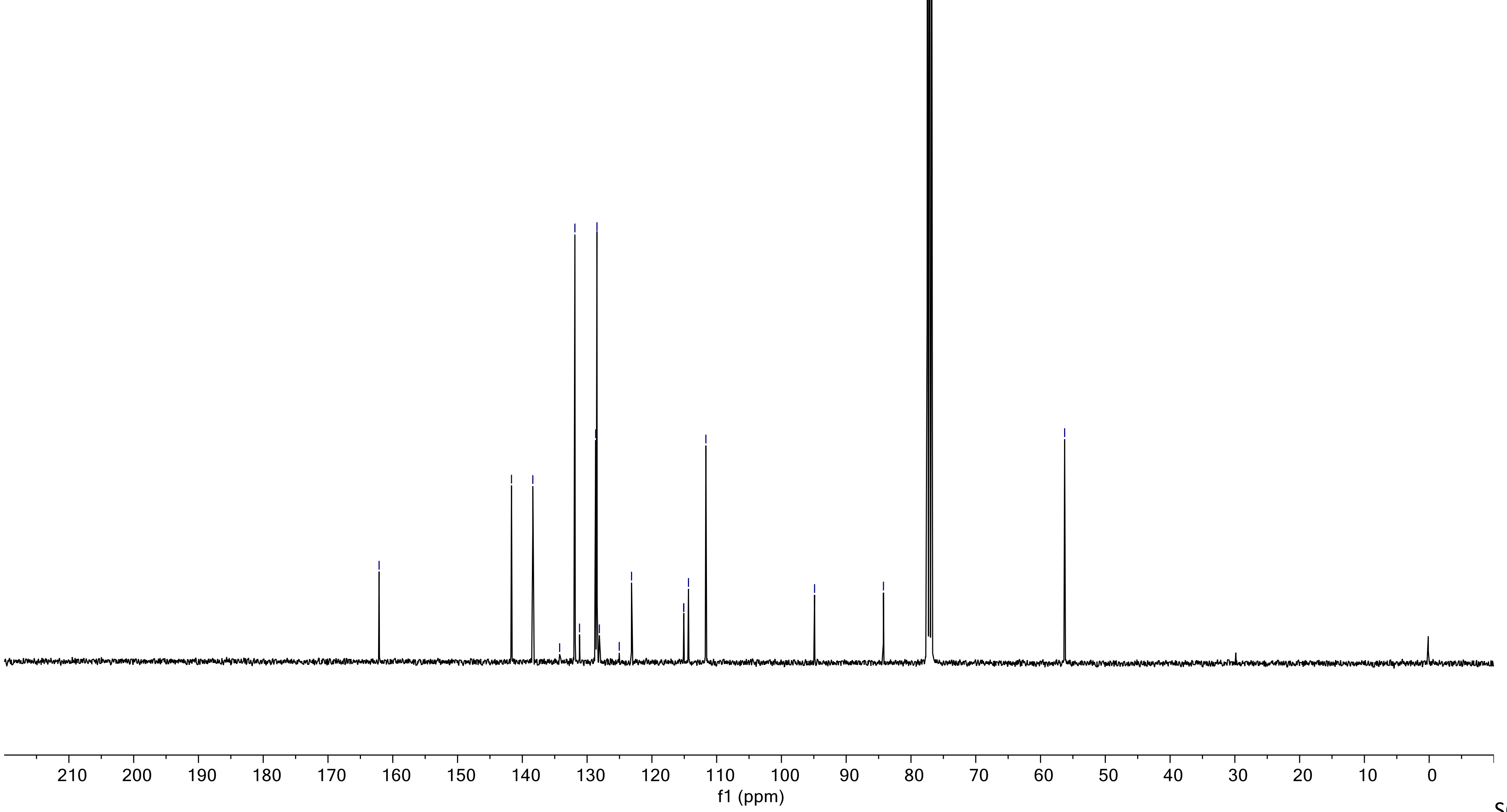

Supplement: Supplementary file 1 — jo3c00454_si_001.pdf [file jo3c00454_si_001.pdf]
